# Study of phenoxy radical couplings using the enzymatic secretome of *Botrytis cinerea*

**DOI:** 10.3389/fchem.2024.1390066

**Published:** 2024-05-28

**Authors:** Robin Huber, Laurence Marcourt, Fabien Félix, Sébastien Tardy, Emilie Michellod, Leonardo Scapozza, Jean-Luc Wolfender, Katia Gindro, Emerson Ferreira Queiroz

**Affiliations:** ^1^ School of Pharmaceutical Sciences, Centre Médical Universitaire (CMU), University of Geneva, Geneva, Switzerland; ^2^ Institute of Pharmaceutical Sciences of Western Switzerland, Centre Médical Universitaire (CMU), University of Geneva, Geneva, Switzerland; ^3^ Mycology Group, Research Department Plant Protection, Nyon, Switzerland

**Keywords:** enzymatic secretome, *Botrytis cinerea*, phenoxy radical, coupling, laccase, phenol, stilbene, phenylpropanoids

## Abstract

Phenoxy radical coupling reactions are widely used in nature for the synthesis of complex molecules such as lignin. Their use in the laboratory has great potential for the production of high value compounds from the polyphenol family. While the enzymes responsible for the generation of the radicals are well known, the behavior of the latter is still enigmatic and difficult to control in a reaction flask. Previous work in our laboratory using the enzymatic secretome of *B. cinerea* containing laccases has shown that incubation of stilbenes leads to dimers, while incubation of phenylpropanoids leads to dimers as well as larger coupling products. Building on these previous studies, this paper investigates the role of different structural features in phenoxy radical couplings. We first demonstrate that the presence of an exocyclic conjugated double bond plays a role in the generation of efficient reactions. In addition, we show that the formation of phenylpropanoid trimers and tetramers can proceed via a decarboxylation reaction that regenerates this reactive moiety. Lastly, this study investigates the reactivity of other phenolic compounds: stilbene dimers, a dihydro-stilbene, a 4-*O*-methyl-stilbene and a simple phenol with the enzymatic secretome of *B. cinerea*. The observed efficient dimerization reactions consistently correlate with the presence of a para-phenol conjugated to an exocyclic double bond. The absence of this structural feature leads to variable results, with some compounds showing low conversion or no reaction at all. This research has allowed the development of a controlled method for the synthesis of specific dimers and tetramers of phenylpropanoid derivatives and novel stilbene derivatives, as well as an understanding of features that can promote efficient radical coupling reactions.

## 1 Introduction

Phenoxy radical couplings have been known for over 100 years and are ubiquitous in nature (Mazzaferro et al., 2015). They are involved in the biosynthetic pathways of natural products to form many polycyclic metabolites (Tang et al., 2017). In particular, these reactions are used in the biosynthesis of key components of plants: the lignans and the complex polymer lignin (Mazzaferro et al., 2015; Tobimatsu and Schuetz, 2019; Thach and Maimone, 2021). The synthesis of the latter is still a poorly understood topic (Tobimatsu and Schuetz, 2019; Thach and Maimone, 2021). In nature, phenoxy radical couplings are mostly carried out by laccase, peroxidase or cytochrome P450 enzymes, which are able to generate radicals from phenolic moieties (Tobimatsu and Schuetz, 2019; Zetzsche et al., 2022). At the laboratory level, phenoxy radical coupling based on chemical methods is also widely used and has recently been reviewed (Carson and Kozlowski, 2024). In all cases, the steps following radical formation are known to occur in solution and are therefore not enzymatically driven (Mate and Alcalde, 2017), in apparent contradiction to the control that plants have with lignin (Tobimatsu and Schuetz, 2019). The so-called “dirigent proteins” have been shown to play a key role in tuning selectivity in some specific cases (see the work on coniferyl alcohol by Davin et al. (1997)), but their function and mechanism are still under investigation (Paniagua et al., 2017).

Previous work has shown that the phytopathogenic fungus *B. cinerea* Pers. produces laccases (Dubernet et al., 1977; Fortina et al., 1996). Based on this knowledge, we have recently used a mixture of secreted enzymes, the “secretome”, containing these laccases obtained from the culture of *B. cinerea* (K16 strain, see the experimental section for more details) as a tool to produce natural product derivatives for drug discovery applications. Using this approach, a series of dimeric stilbene derivatives have been obtained, some of which exhibit potent antifungal, antiviral, and antibacterial properties (Gindro et al., 2017; Righi et al., 2020; Huber et al., 2022a; Huber et al., 2023; Zwygart et al., 2023). Although trimers and tetramers of stilbenes have been isolated and characterized in many plants (Shen et al., 2017), it has not been possible to generate them using this chemo-enzymatic approach starting from stilbene monomers. In contrast, the use of phenylpropanoids as starting material, under the same reaction conditions, led to the production of dimers, as well as trimers, tetramers, and probably larger insoluble polymers (Huber et al., 2022b).

**FIGURE 1:**
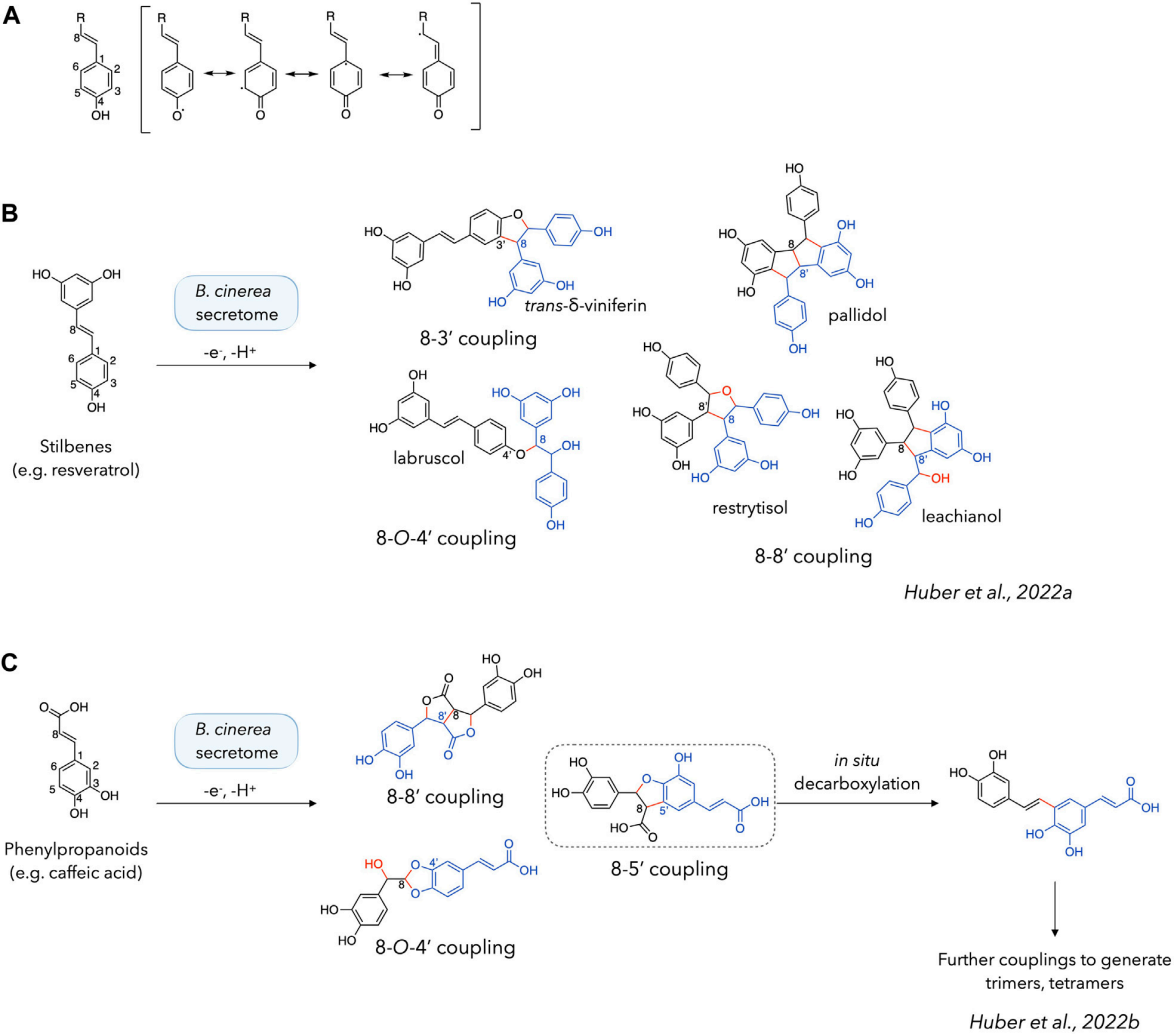
Phenoxy radical coupling reactions of stilbenes and phenylpropanoids. **(A)** Delocalization of the radical formed on the phenolic function. **(B, C)** Products obtained by phenoxy radical coupling using stilbenes (e.g., resveratrol) (Righi et al., 2020; Huber et al., 2022a) **(B)** or phenylpropanoids (e.g., caffeic acid) (Huber et al., 2022b) **(C)** as starting materials. Dimeric structures are shown with one monomer in black and another in blue, with the new bonds and atoms in red.

Phenoxy radical coupling reactions are known to be initiated by the formation of a radical on a phenolic function. This radical is delocalised on the benzene ring and, where appropriate, along the chain conjugated to it. In the case of stilbenes, the entire system is conjugated (Figure 1A). Looking at the products obtained from the reactions with these compounds, it can be seen that each dimeric product has its new bond formed between a C-8 position (on the exocyclic double bond) and another position. The latter can be another C-8 position (pallidol, restrytisol and leachianol scaffolds), a C-3 position (*trans*-δ-viniferin scaffold) or an *O*-4 position (acyclic dimer/labruscol (Nivelle et al., 2017) scaffold) (Figure 1B) (Gindro et al., 2017; Righi et al., 2020; Huber et al., 2022a).

While Liu et al., 2022 mentioned that a conjugated exocyclic double bond “may” be involved in phenoxy radical coupling, our previous investigations tend to show that it is systematically involved when present (Gindro et al., 2017; Righi et al., 2020; Huber et al., 2022a). Indeed, this is also consistent with their results for isoeugenol or coniferyl alcohol, the only two compounds in their set with a conjugated double bond at the C-7/C-8 position (Liu et al., 2022). The systematic involvement of the C-8 position seems to indicate that the radical is more frequently localized at this position, which could be explained by its relative stability as a para-quinone methide, analogue to a *p*-quinone. The presence of *trans*-δ-viniferin (resulting from a C-8/C-3 coupling) as the main product when working with resveratrol, and not a C-8/C-8′ coupling product (pallidol, restrytisol, leachianol) (Gindro et al., 2017) could be seen as a discrepancy. This point can however be mitigated by the fact that the C-8/C-8′ coupling products are split into several compounds, whereas the C-8/C-3′ coupling produces only one compound: *trans*-δ-viniferin.

Furthermore, our previous results using phenylpropanoids as substrates also indicate that each product obtained involves the formation of at least one bond at a C-8 position. (Figure 1C). In this case, the main compounds are obtained by C-8/C-5′, C-8/C-8′ and C-8/*O*-4′ couplings. Taken together, these examples clearly demonstrate the important role of the C-8 position when conjugated to the phenol ring in the generation of efficient laccase-catalysed coupling reactions. The absence of this conjugation chain appears to drastically reduce the reactivity in the case of stilbenes, as the dimers formed do not react further to form tetramers (Gindro et al., 2017; Righi et al., 2020; Huber et al., 2022a). In contrast to stilbenes, phenylpropanoids react further to form trimers, tetramers and presumably larger polymers (Huber et al., 2022b). To explain this formation of higher order compounds, we hypothesized in our previous work that a decarboxylation reaction is involved in the regeneration of the reactive moiety (Huber et al., 2022b) (Figure 1C). However, it should be mentioned that efficient phenoxy radical coupling has also been demonstrated in the absence of a conjugated exocyclic double bond, for example with vanillyl alcohol, isoeugenol and other simple phenols (Llevot et al., 2016).

To further explore the behaviour of phenoxy radical coupling reactions with the *B. cinerea* enzymatic secretome containing laccases, this study presents results on several phenols to better understand the structural features associated with efficient reactions. First, the formation of phenylpropanoid tetramers was dissected in independent, controlled steps, highlighting the role of a decarboxylation reaction. The second part of this article focuses on phenols with different structural features to test their influence on the formation of coupling products.

## 2 Results and discussion

### 2.1 Phenylpropanoid trimers and tetramers can be generated through the regeneration of the conjugated exocyclic double bond by decarboxylation

**FIGURE 2:**
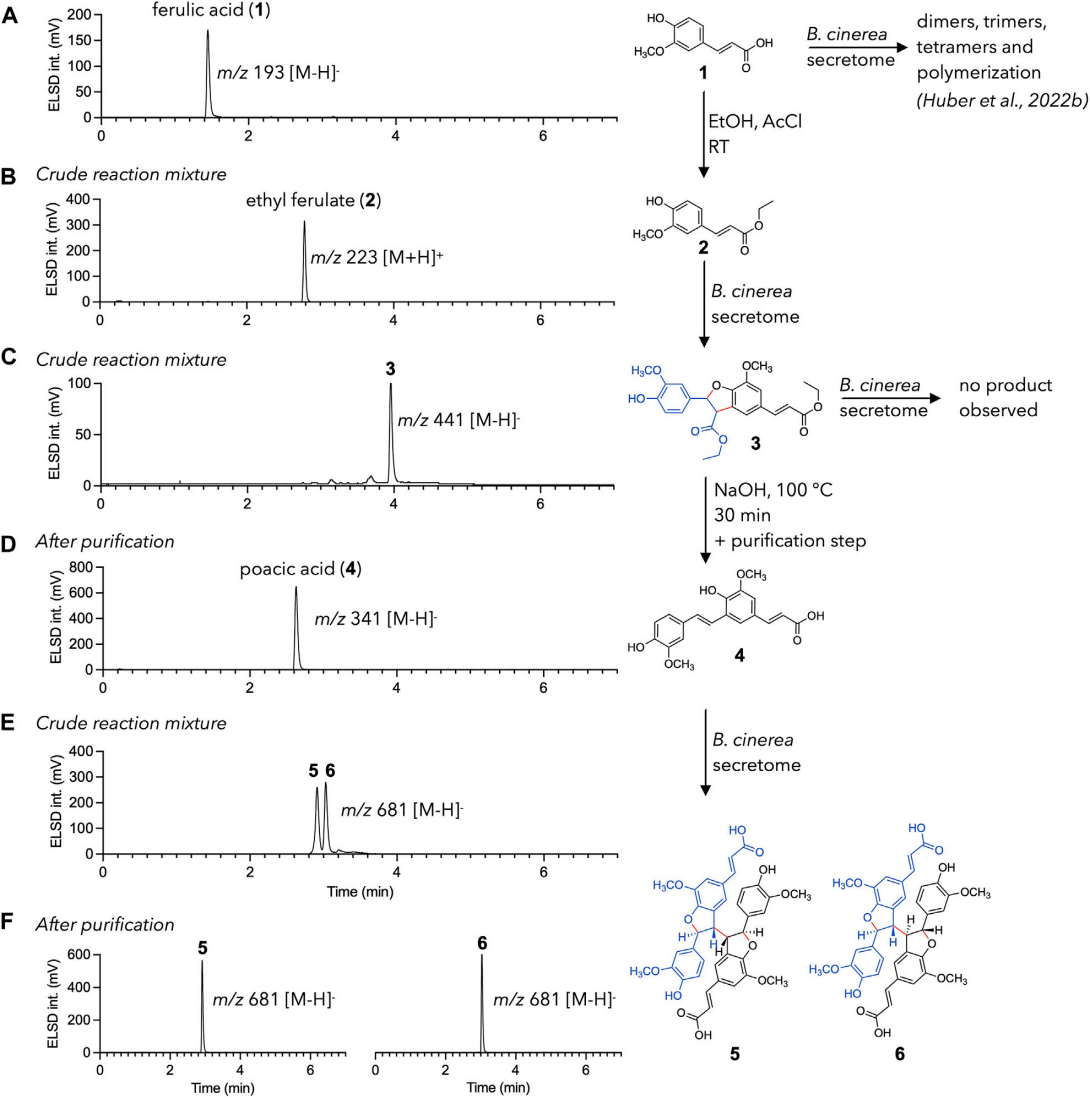
Directed radical coupling reaction of ferulic acid (**1**). UHPLC-ELSD chromatograms are shown for each step. **(A)** ferulic acid **1** is used as starting material. **(B)** Esterification leads to ethyl ferulate (**2**). **(C)** Ethyl ferulate (**2**) is efficiently converted to **3** using the enzymatic secretome of *Botrytis cinerea* **(D)** Ester cleavage—decarboxylation process of **3** results in the formation of poacic acid (**4**). Purification is necessary at this step as side products are also formed. **(E)** Poacic acid (**4**) is dimerized into diastereoisomers **5** and **6** using the enzymatic secretome of *B. cinerea*. **(F) 5** and **6** are easily separated to give the pure diastereoisomers.

As presented in the introduction, the formation of phenylpropanoid trimers and tetramers has already been discussed in our previous article, where mechanisms of formation were proposed for the various products observed (Huber et al., 2022b). In each case, the formation of structures larger than dimers was explained by a decarboxylation reaction of the dimer obtained by C-8/C-5′ coupling, leading to the recycling of the conjugated exocyclic double bond (Figure 1C). This structure could then be converted again into a radical and react in solution with other radical monomers to form trimers, or dimers to form tetramers. This hypothesis is investigated here by dividing the reaction cascade (radical formation and dimerization, decarboxylation, and second radical formation and dimerization) into separated, controlled steps carried out on ferulic acid (**1**). Each step was precisely monitored by UHPLC-PDA-ELSD-MS (Figure 2).

To this end, decarboxylation was inhibited by converting the carboxylic acid of ferulic acid to an ester. This was easily achieved by incubating ferulic acid in ethanol with acetyl chloride (Yue et al., 2017). The UHPLC monitoring allowed to make sure that the starting material was fully consumed (Figure 2B). Ethyl ferulate (**2**) was then incubated with the enzymatic secretome of *B. cinerea*, under conditions similar to those described in Huber et al. (2022b). Quantification of laccases in the fungal secretome of *B*. *cinerea* was carried out using the guaiacol assay method (Abd El Monssef et al., 2016) and gave a result of 0.9 U/L (see experimental section). The biotransformation reaction yielded mainly a dimer (*m/z* 441, [M-H]^-^) (Figure 2C). NMR analysis confirmed its identity as an ethyl ferulate dimer (**3**) (see the experimental section). This compound did not react further on prolonged incubation with the enzymatic secretome. This was expected since esterification blocked any decarboxylation reaction.

The following step was a saponification/neutralization step to convert the two esters back to carboxylic acids. This hydrolysis was intended to be followed by an immediate decarboxylation to produce poacic acid (**4**). In fact, this step was not as straightforward as expected, as the nature and relative amounts of the products obtained were highly dependent on the hydrolysis temperature and duration. A first attempt at 50°C in a 0.35 M NaOH solution was unsuccessful for poacic acid (**4**) generation in a reasonable yield but resulted in the production of other ferulic acid dimers (**7** and **8**) and a degradation product (**9**) (see Supplementary Figure S1).

A second trial was carried out in a boiling 0.35 M NaOH solution. Reaction’s samples were monitored at 10 min, 2 h, 6 h, and 22 h. Poacic acid (**4**) was obtained in good yield between 10 min and 6 h but disappeared upon prolonged times. It was still accompanied by smaller amount of compound **8** (Supplementary Figure S1). The saponification—decarboxylation reaction was therefore carried out at a higher scale for 30 min at reflux. The crude reaction mixture was purified by flash-chromatography, yielding 150 mg of pure poacic acid (**4**) (yield of 9.7% relative to compound **2)** (Figure 2D).

Poacic acid (**4**) was finally subjected to a biotransformation reaction with the *B. cinerea* secretome. Since the conjugation chain to the C-8 position was regenerated by decarboxylation, dimerization was expected to happen and to produce compounds **5** and **6**, previously isolated from the biotransformation reaction of ferulic acid (Huber et al., 2022b). The result matched the expectation and gave two very major compounds, that were isolated by semi-preparative HPLC-UV (Figures 2E,F). It should be noted that despite the absence of other intense signals (in ELSD, MS, and UV, see Supplementary Figure S2), the total mass of the isolated compounds was only one-third of that of the substrate. This is partly explained by the usual loss in preparative liquid chromatography, but could also indicate the formation of other, undetected compounds. NMR analysis allowed to confirm the identity of the isolated compounds as **5** and **6**. As a note, these two compounds were obtained here in higher amount and purity as compared to our previous study, thanks to the reduced complexity of the mixture (see Supplementary Figure S3 for more details) (Huber et al., 2022b).

This experiment shows that a simple reaction (in this case esterification) can avoid decarboxylation and thus completely change the reactivity and nature of the compounds obtained. Thus, the ferulic acid dimer **7**, poacic acid **4**, or tetramers **5** and **6** were selectively obtained in higher yields and purities (see Supplementary Material for more details). These results also highlight that, under our reaction conditions, the decarboxylation reaction plays a key role in the formation of phenylpropanoid tetramers, as hypothesized in our previous article (Huber et al., 2022b). This approach might be further extended by incubating poacic acid (**4**) with ferulic acid to obtain phenylpropanoid trimers that were previously reported (Huber et al., 2022b).

### 2.2 Reactivity of other phenols with laccases

The previous section emphasized the role of the conjugated exocyclic double bond in the coupling reactions of phenylpropanoids. Notably, stilbene and phenylpropanoid dimers lacking this feature did not produce larger coupling products upon prolonged incubation times with the enzymatic secretome of *B. cinerea* (see Figure 1). However, previous studies showed that dimers of the simple phenol (hydroxybenzene) were obtained by radical coupling when incubated with 2,2-diphenyl-1-picrylhydrazyl (DPPH), despite the absence of this reactive moiety (Liu et al., 2022). As mentioned in the introduction, other small phenols such as vanillyl alcohol or isoeugenol have also been efficiently coupled using laccases (Llevot et al., 2016). Thus, the behavior of different phenols when incubated with the enzymatic secretome of *B. cinerea* was investigated.

For this purpose, a set of six phenolic compounds was selected as follows: 1) resveratrol was used as a positive control, since its total conversion into dimers has been extensively studied (Gindro et al., 2017; Righi et al., 2020; Huber et al., 2022a). 2) The resveratrol dimer, *trans*-δ-viniferin, was also included as a negative control, as its lack of dimerization when incubated with laccases was previously shown (Gindro et al., 2017; Righi et al., 2020; Huber et al., 2022a). 3) *Trans*-ε-viniferin, another resveratrol dimer found in grapes (*Vitis vinifera* L.), was also selected (Shen et al., 2009). This compound is produced in plants by a C-8/C-10′ coupling, leaving a conjugation chain between the C-8′ carbon and a free para-phenol on one of the resveratrol moieties (Fuloria et al., 2022). This compound has previously been shown to yield resveratrol tetramers when incubated with a peroxidase (Takaya et al., 2002). 4) 4-*O*-methyl-resveratrol is a resveratrol derivative in which the para-phenol is protected as a methyl ether. It was chosen to test the reactivity of the phenols localized on the resorcinol ring. 5) The role of the C-7/C-8 exocyclic double bond was further investigated using dihydro-resveratrol, a resveratrol analogue obtained by reduction of this double bond. 6) Phenol was used as the simplest substrate in this type of reaction.

**FIGURE 3:**
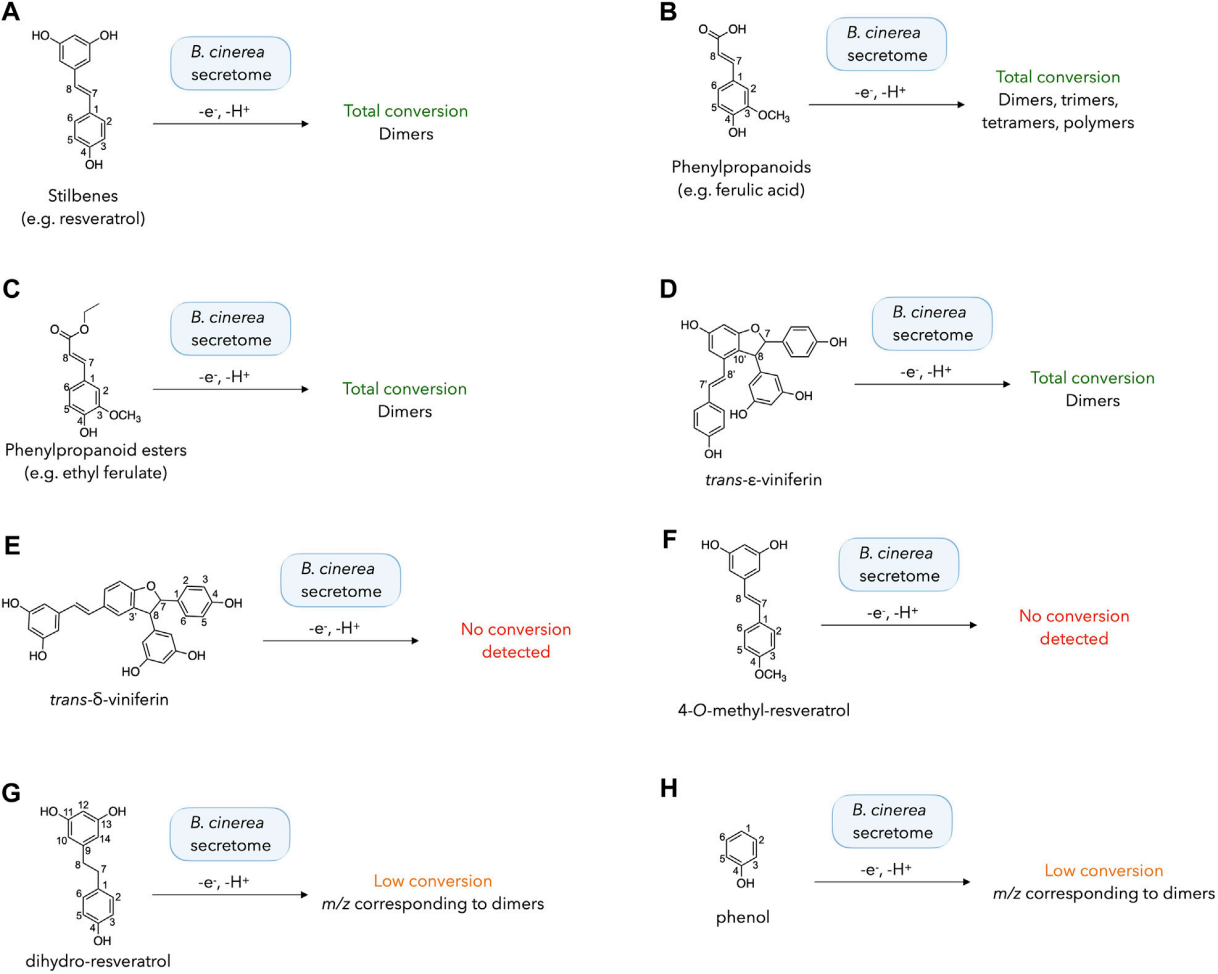
Biotransformation reactions performed with different phenolic compounds **(A–H)** using the enzymatic secretome of *Botrytis cinerea*. The results of each reaction were evaluated by UHPLC-PDA-ELSD-MS analysis after 48 h. The detailed chromatograms for each reaction are shown in Supplementary Figure S4.

The different substrates were incubated in the following conditions: two incubations with 5% and 20% of the enzymatic secretome of *B. cinerea*, and a negative control without enzymes. Mixtures were incubated for 48h, extracted and analyzed by UHPLC-PDA-ELSD-MS. For each substrate, the chromatographic profiles at 5% and 20% secretome were very similar and the negative control did not give any product. The results are summarized in Figure 3, also including the phenylpropanoids and phenylpropanoid esters described above. The detailed chromatograms are shown in Supplementary Figure S4.

The presence of a para-phenol conjugated to an exocyclic double bond is systematically correlated with a total consumption of the starting material with small amount of secretome (5%). This result can be seen for stilbenes (e.g. resveratrol), phenylpropanoids (e.g. ferulic acid), phenylpropanoid ester (e.g. ethyl ferulate) and *trans*-ε-viniferin (Figures 3A–D; Supplementary Figures S4A–D). In the latter case, several signals corresponding to resveratrol tetramers (*m/z* 905 corresponding to the [M-H]^-^ adduct) were detected, matching the results obtained with a peroxidase (horseradish peroxidase) by Takaya et al., 2002, who reported the generation of several resveratrol tetramers such as hopeaphenol, isohopeaphenol, vitisin B, and vitisin C.

For compounds lacking either the free para-phenol or the exocyclic double bond, the results were more variable (Figures 3E–H; Supplementary Figures S4E–H). In the case of *trans*-δ-viniferin, no dimeric product was detected (corresponding to resveratrol tetramers, as detected upon incubation of *trans*-ε-viniferin), even with a large secretome amount (20%). This result is coherent with the absence of tetramers generated in our previous research with stilbenes (Righi et al., 2020; Huber et al., 2022a). Similarly, incubation of 4-*O*-methyl-resveratrol did not yield dimers, suggesting that dimerization does not occur when only the phenols of the resorcinol ring are available, consistent with density functional theory (DFT) calculations indicating that the meta-phenols of resveratrol are difficult to oxidize (Cao et al., 2003). However, the results obtained with the other compounds mitigate this simplistic vision. Incubation of dihydro-resveratrol (lacking the exocyclic double bond) led to the formation of dimeric compounds (*m/z* 457, [M-H]^-^) with 5% or 20% of *B. cinerea* secretome. This result is in agreement with that obtained with a peroxidase from *Momordica charantia* L. (Xie et al., 2009). However, the conversion was very low and much of the starting compound was not converted under our reaction conditions. Using the simplest phenol as a substrate, a diphenol (*m/z* 185 [M-H]^-^) was produced with a very low conversion using both 5% and 20% of the enzymatic secretome.

**FIGURE 4:**
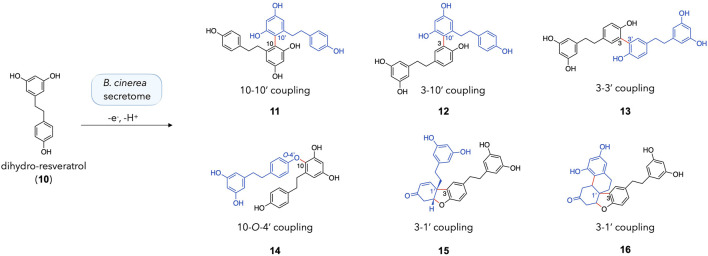
Dimeric products obtained with the incubation of dihydro-resveratrol (**10**) with the enzymatic secretome of *Botrytis cinerea*.

As it was initially thought that incubation of dihydro-resveratrol with the secretome of *B. cinerea* would not result in the formation of dimers, it was decided to further investigate the compounds generated and their similarity to those obtained with a peroxidase (Xie et al., 2009). The reaction was repeated on a larger scale (40 mg of dihydro-resveratrol **10**). The crude reaction was monitored by UHPLC-PDA-ELSD-MS and the generated compounds were purified by semi-preparative HPLC-UV scale according to the protocols of gradient transfer and dry load injection developed in our laboratory (Queiroz et al., 2019) (see the experimental section). This reaction led to the formation of four dimers with the following coupling patterns: a C-10/C-10′ coupling (**11**), a C-3/C-10′ coupling (**12**), a C-3/C-3′ coupling (**13**), and a C-10/*O*-4′ coupling (**14**) (Figure 4). These results are in good agreement with Xie et al (2009). It should be noted that **11** and **12** are the first dimers obtained by coupling involving the C-10 position (on the resorcinol moiety) using our *B. cinerea* secretome and stilbenes.

In addition, two compounds bearing a Pummerer’s ketone scaffold were obtained: **15** and **16**. Both were probably obtained by a C-3/C-1′ coupling, followed by several keto/enol tautomerization and cyclization steps. The proposed mechanism for the formation of **15** and **16** is shown in Supplementary Figure S5. According to this reaction mechanism, **15** is probably a precursor of **16**. A close derivative of this compound (with phenyl rings instead of phenols) was recently reported for the first time by Sarkar *et al.* in a paper describing the synthesis of Pummerer’s ketones (Sarkar et al., 2020). Both **15** and **16** are new compounds and are described hereafter.

**FIGURE 5:**
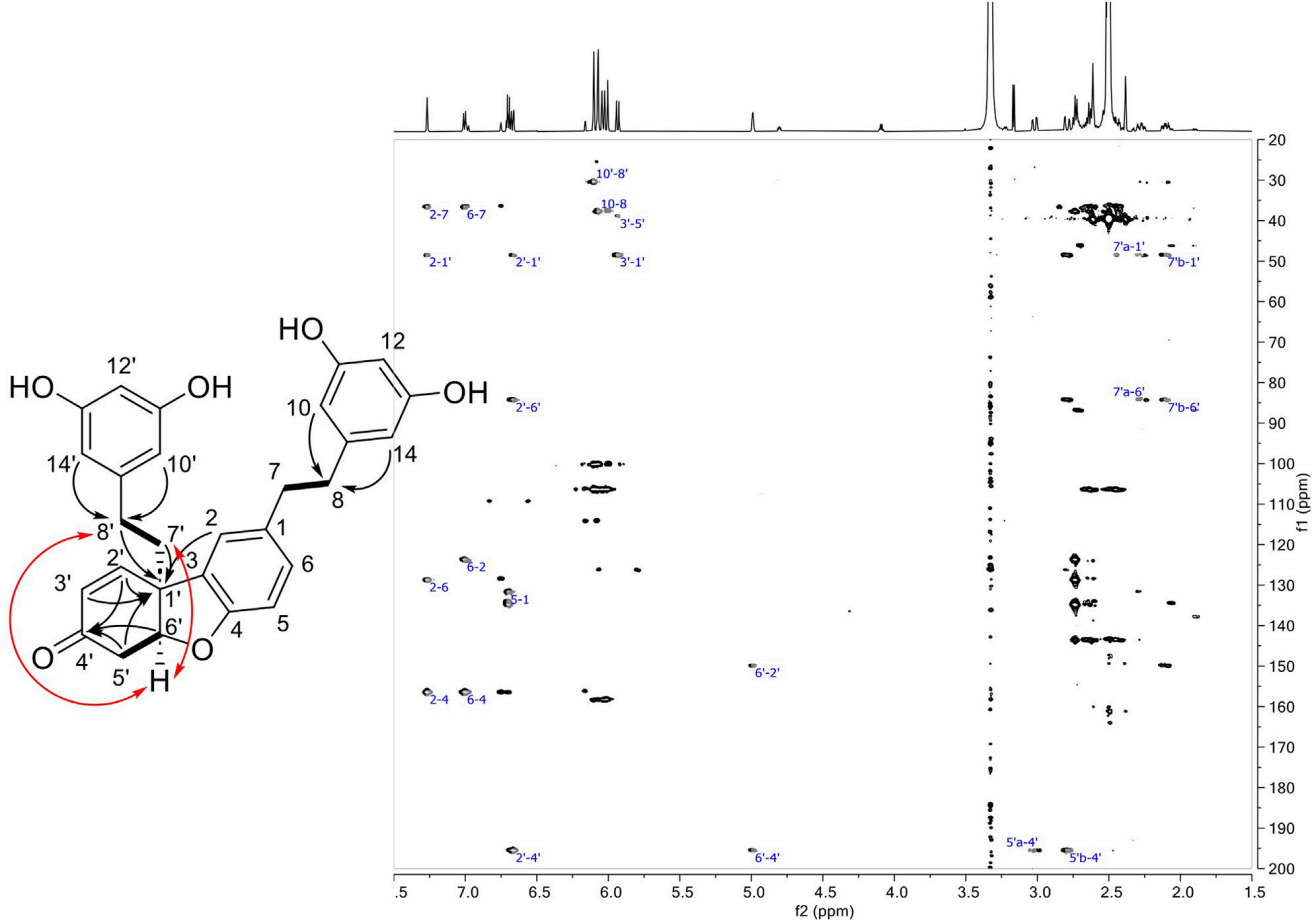
Left: Structure of compound **15** showing COSY (bond in bold), HMBC (black arrows) and ROESY correlations (red arrows). Right: HMBC spectrum of **15**.

Compound **15** was isolated in mixture with **16** (1:0.3 mol). Its ^1^H NMR spectrum showed characteristic signals from two 3,5-diphenol rings, i.e., a triplet (accounting for 1H) and a doublet (accounting for 2H) with meta coupling (*J* = 2.2 Hz) at δ_H_ 6.03 (H-12) and 6.07 (H-10, H-14) for the former and 6.04 (H-12′), 6.10 (H-10′, H-14′) for the latter. The presence of a third aromatic group consisting of a doublet (*J* = 1.9 Hz) at δ_H_ 7.27 (H-2), a doublet of doublet (*J* = 8.2, 1.9 Hz) at δ_H_ 7.01 (H-6) and a doublet (*J* = 8.2 Hz) at δ_H_ 6.70 (H-5) indicated that a substitution occurred at position C-3 of the first dihydro-resveratrol moiety. Only two protons of the fourth ring remain in the aromatic region at δ_H_ 6.67 (1H, dd, *J* = 10.2, 1.9 Hz, H-2′) and 5.93 (1H, d, *J* = 10.2 Hz, H-3′). The HSQC spectrum showed the presence of an oxygenated methine at δ_H_ 4.99 (δ_C_ 84.3) and five methylenes at δ_C_ 30.4 (CH_2_-8′), 36.5 (CH_2_-7′), 36.6 (CH_2_-7), 37.9 (CH_2_-8), and 38.9 (CH_2_-5′), indicating a partial hydrogenation of the phenol ring from the second dihydro-resveratrol moiety. The four methylenes CH_2_-7 and CH_2_-8 (and CH_2_-7′ and CH_2_-8′) were identified from the HMBC correlations from H-10/H-14 to CH_2_-8 (H-10′/H-14′ to CH_2_-8′) and from the COSY correlation from H_2_-8 at δ_H_ 2.64 (t, *J* = 8.1 Hz) to H_2_-7 at δ_H_ 2.74 (t, *J* = 8.1 Hz) (H_2_-8′ at δ_H_ 2.44 to H-7′b at δ_H_ 2.10 and H-7′a at δ_H_ 2.27) (Supplementary Table S1). The COSY correlations from the oxygenated methine H-6′ to the methylene H_2_-5′ (δ_H_ 2.79, dd, *J* = 17.6, 2.7 Hz, and 3.02 dd, *J* = 17.6, 4.1 Hz) and the HMBC correlations from H-6′, H_2_-5′ and H-2′ to a carbonyl at δ_C_ 195.5 (C-4′), from H-2′, H-3′, H-5′b, H_2_-8′, H_2_-7′ and H-2 to a quaternary carbon at δ_C_ 48.4 (C-1′) indicated that i) the phenolic ring of the second dihydro-resveratrol moiety was converted to a cyclohexenone, ii) the linkage of the two dihydro-resveratrol moieties was between C-3 and C-1′. A second linkage was inferred between C-6′ and the oxygen at C-4 in agreement with the HRMS data and due to the absence of phenolic signal at C-4. The other phenolic protons were observed at δ_H_ 9.04 for OH-11/OH-13 and 9.07 for OH-11′ and OH-13′. Finally, the ROESY correlation from H-6′ to H_2_-8′ and H-7′b indicated the relative *cis* configuration of H-6′ and H_2_-7′ (Figure 5; Supplementary Table S1).

**FIGURE 6:**
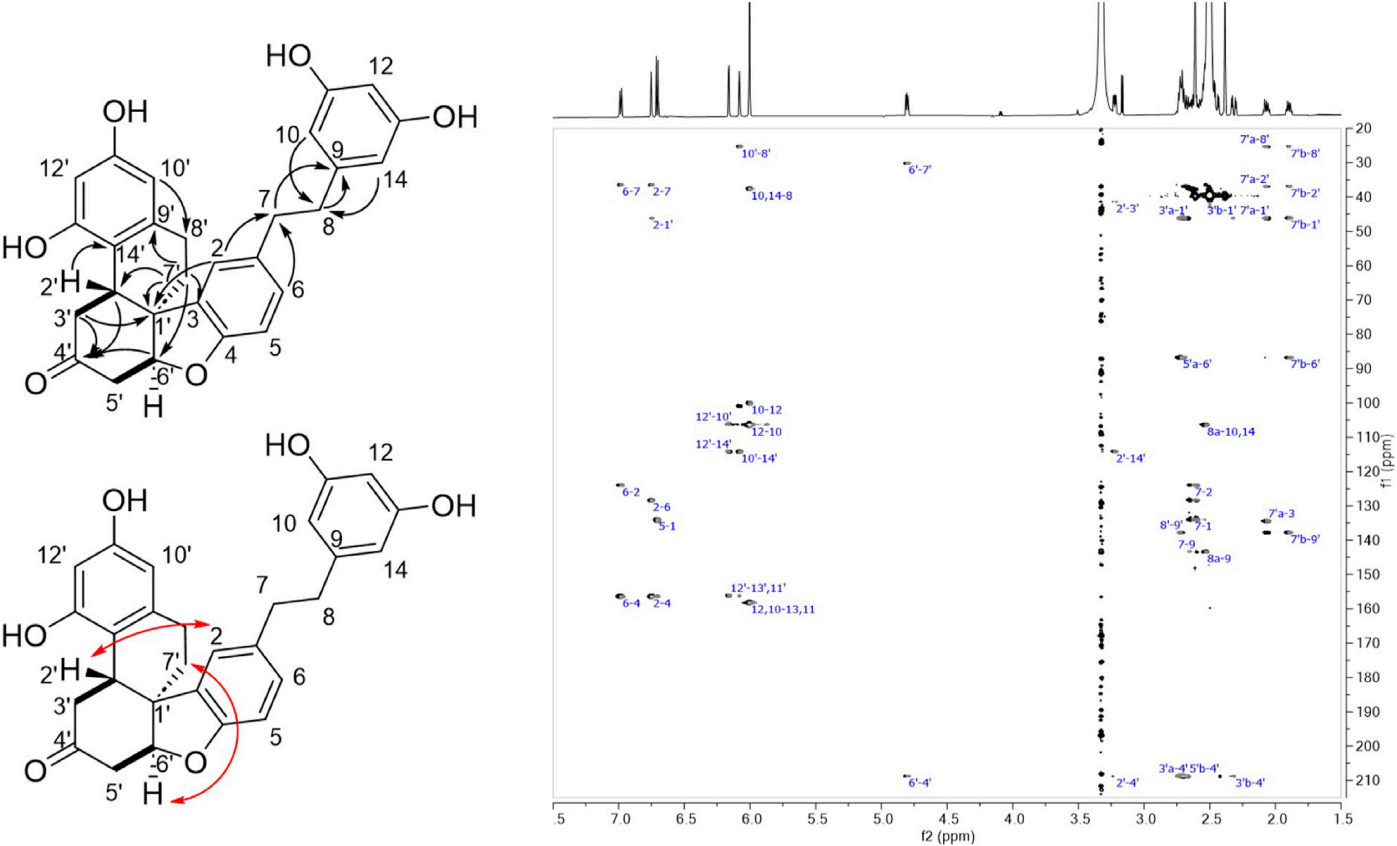
Left: Structure of compound **16** showing COSY (bond in bold), HMBC (black arrows) and ROESY correlations (red arrows). Right: HMBC spectrum of **16**.

The ^1^H and HSQC NMR spectra of compound **16** revealed the presence of eight aromatic protons corresponding to three aromatic cycles. A 1,3,4-tribstituded benzene with a doublet (*J* = 8.1 Hz) at δ_H_ 6.71 (H-5), a doublet (*J* = 1.9 Hz) at δ_H_ 6.75 (H-2) and a doublet of doublet (*J* = 8.1, 1.9 Hz) at δ_H_ 6.99 (H-6) were observed for the first aromatic ring. These signals were assigned to the phenolic group of a dihydro-resveratrol with a substitution at C-3. The singlet at δ_H_ 6.00 integrating for 3 protons and showing HSQC correlations with 2 carbons at δ_C_ 100.1 (CH-12) and 106.4 (CH-10, CH-14) were assigned to the 3,5-diphenol ring of resveratrol. The two doublets (*J* = 2.4 Hz) at δ_H_ 6.08 (H-10′) and 6.16 (H-12′) could originate from the 3,5-diphenol ring of the second dihydro-resveratrol with a substitution at C-14′. In addition, six methylenes at δ_C_ 25.4 (CH_2_-8′), 30.1 (CH_2_-7′), 36.5 (CH_2_-7), 37.7 (CH_2_-8), 41.4 (CH_2_-3′), 42.3 (CH_2_-5′), and two methines at δ_C_ 37.0 (CH-2′), 86.7 (CH-6′) were observed on the HSQC spectrum. As for compound **15**, **16** was shown to be a dihydro-resveratrol dimer with one of the phenol groups dearomatized. The ethylenic signals of each dihydro-resveratrol were assigned by the following HMBC correlations: from H-10/H-14 (δ_H_ 6.00) to CH_2_-8 (δ_C_ 37.7), from H-2/H-6 (δ_H_ 6.75/6.99) to CH_2_-7 (δC 36.5), and from H_2_-8 (δ_H_ 2.49/2.53) and H_2_-7 (δ_H_ 2.63) to C-9 (δ_C_ 143.5) for the first monomer; from H-10′ (δ_H_ 6.08) to CH_2_-8′ (δ_C_ 25.4) and from H_2_-7′ (δ_H_ 1.90/2.07) to C-9′ (δ_C_ 137.8), a quaternary carbon C-1′ (δ_C_ 46.1) and the two methines CH-2′ (δ_C_ 37.0) and CH-6′ (δ_C_ 87.6) for the second monomer (Supplementary Table S1). The COSY correlations from H-2′ (δ_H_ 3.23) to H_2_-3′ (δ_H_ 2.32/2.69) and from H-6′ (δ_H_ 4.80) to H_2_-5′ (δ_H_ 2.45/2.72) as well as the HMBC correlations from H-2′, H_2_-3′, and H-6′ to a carbonyl C-4′ (δ_C_ 208.8) and from H_2_-3′ to C-1′ indicated that a cyclohexanone substituted in C-2′ and C-6′ replaced the phenolic group of the second dihydro-resveratrol. The HMBC correlation from H-2′ to C-14′ (δ_C_ 114.2), from H-2 to C-1′ and from H_2_-7′ to C-3 indicated a bond between C-2′/C-14′ and C-1′/C-3. For the same reasons as for **15**, an ether was positioned between C-6′ and C-4. As with **15**, the ROESY correlations from H-6′ and H_2_-7′ allowed the relative configuration between H-6′ and H_2_-7′ to be defined as *cis*. Few correlations were observed with H-2′, but the one with H-2 seems to indicate that H-2′ was *trans* compared to H-6′ and H_2_-7′ (Figure 6; Supplementary Table S1).

Taken together, the reactivity of the selected series of phenols highlights the complexity of the radical coupling reactions. However, it is clear from these results that the presence of a conjugated exocyclic double bond in para-position to a phenol is associated with high catalytic efficiency and total conversion in every case studied. This was observed in the case of stilbenes and phenylpropanoids, where main products were obtained by dimerization reactions involving at least one C-8 position (Figure 1) which was in agreement with the literature. For example, in the case of resveratrol, the pioneering work of Langkage and Pryce (Langcake and Pryce, 1977) showed that the main compound obtained was *trans*-δ-viniferin, derived from a C-8/C-3′ dimerisation using horseradish peroxidase. These results have since been observed in numerous studies on laccases and peroxidases (Wilkens et al., 2010; Gindro et al., 2017; Righi et al., 2020; Huber et al., 2022a; Park et al., 2022). This point could be explained by the fact that the presence of an exocyclic conjugated double bond increases the delocalization of the radical and thus its stability. This improved stability probably increases its lifetime in solution and leads to a better conversion.

It should be noted that, in addition to C-8 dimerisation products, other compounds can be obtained, such as *trans*-ε-viniferin, iso-*trans*-ε-viniferin or iso-*trans*-δ-viniferin described by Sursin et al. (2023). However, these compounds are obtained in low yields (Sursin et al., 2023). In the case of iso-*trans*-ε-viniferin, the authors proposed a possible mechanism involving a dismutation of the radical species leading to cationic forms (Sursin et al., 2023).

In contrast, the absence of an exocyclic conjugated double bond results in significantly lower reaction yields under our reactions conditions, probably due to reduced stability and lifetime of the radicals. In such situation, dimerization can occur at unusual positions, as illustrated by the products generated from dihydro-resveratrol (C-10/C-10′, C-3/C-10′, C-10/*O*-4′ couplings).

Among our results, it remains difficult to explain the absence of detectable products upon incubation of 4-*O*-methyl-resveratrol with the enzymatic secretome of *B. cinerea* after the obtention of compounds **11**, **12,** and **14**. Indeed, these compounds prove that the meta-phenols can be oxidized to radicals. Dimerization of 4-*O*-methyl-resveratrol was however reported using a COX-1 enzyme (cyclooxygenase, prostaglandin synthase) (Szewczuk et al., 2005).

This highlights again marked differences in the results of phenoxy radical coupling with different enzymes and conditions. 

Furthermore, the absence of detectable products when incubating *trans*-δ-viniferin which holds a monophenol and two resorcinol moieties is also difficult to explain. Steric hindrance could be an explanation limiting the reactivity of *trans*-δ-viniferin with laccases. However, it seems strange that the resveratrol dimer *trans*-ε-viniferin is sterically tolerated and that all the others dimers (pallidol, leachianol, restrytisol, *trans*-δ-viniferin), which have the same molecular weight, are not accepted as substrates by these enzymes. Perhaps the explanation lies in a combination of 1) steric hindrance and 2) limited reactivity due to the absence of a stabilizing conjugated exocyclic double bond.

## 3 Conclusion

Previous studies in our laboratory highlighted the important role of the conjugated exocyclic double bond at the C-7/C-8 position in achieving efficient phenoxy radical coupling reactions with laccases from the enzymatic secretome of *B. cinerea*. Stilbenes were shown to form dimers by coupling of the C-8 position with other positions (C-8′, C-3′, or *O*-4′). The behavior of phenylpropanoids was however different, yielding not only dimers, but also trimers, tetramers, and larger polymers through coupling of a C-8 position with a C-8′, C-5′ or *O*-4′ one.

This work first focused on understanding the formation mechanism of phenylpropanoid trimers and tetramers. Decarboxylation reactions have been proposed as key steps in the regeneration of the conjugated exocyclic double bond, enabling subsequent reactions with radical monomers or dimers to form larger products. Experimental evidence supports this hypothesis under our reaction conditions, as the blocking of decarboxylation by esterification prevented further reactions beyond dimer formation. In the complex world of nature, other mechanisms must also be involved (other enzymes, dirigent proteins, specific conditions, etc.), as lignin formation is known to result from the polymerization of monolignols, which cannot decarboxylate (Sangha et al., 2014).

Finally, the reactivity of several phenols with laccases was explored. The presence of a conjugated exocyclic double bond was consistently associated with efficient reactions and dimer formation. However, the absence of this feature led to variable results. Some compounds showed low conversion or no detected reaction at all. The presence of the exocyclic conjugated double bond is thus thought to increase radical stability and lifetime and thereby to improve conversion rates. Further research is needed to explore additional factors influencing the reactivity of phenols and to uncover the full complexity of these reactions. A better understanding of these enzymatic reactions will eventually make it possible to use them in a more sophisticated way to create complex molecules with the same precision as nature.

## 4 Experimental section

### 4.1 *Botrytis cinerea* secretome extraction

The *B. cinerea* Pers., K16 strain, was obtained from naturally sporulated grape berries from the Changins Agroscope experimental vineyards in 2015. The strain was purified and identified by sequencing the ITS regions. It was grown on oatmeal agar medium (Difco) to collect conidia by vacuum aspiration for storage at −80°C until further use. The secretome was produced by cultivating *B. cinerea* in 1.5 L liquid medium (in 5 L bottles) at 22°C with a 12 h/12 h day/night cycle for 2 weeks. The medium was filtered on folded filter papers (500 mm, Prat Dumas) to remove the mycelium and the filtrate was precipitated with ammonium sulfate (80% saturation) and centrifugation (4,200 g, 4°C, 2 h). The pellet was resuspended in nanopure water (Evoqua Waters Technologies, 4.2 μScm^−1^). This protein crude extract was dialyzed (Spectra/Por 1 dialysis membrane, 6–8 kDa, diameter 14.6 mm) against nanopure water overnight at 4°C and then concentrated on polytethylene glycol beads (PEG 20,000) in the dialysis tube. The protein amount was determined by the Bradford method, using a Bio-Rad protein assay kit with BSA as a standard. The final volume was corrected to obtain a protein concentration of 2 μg/μL. The resulting extract (referred to as “secretome”) was aliquoted to 1 mL and stored at −20°C until use.

### 4.2 Guaiacol assay for laccase activity measurement

The laccase activity of the batch of *B. cinerea* secretome used for the biotransformation reactions was spectrophotometrically determined using the guaiacol assay method (Abd El Monssef et al., 2016). The reaction mixture contained 1 mL of guaiacol (2-methoxyphenol, 2 mM, *ε* = 0.6740 μM.cm^−1^), 3 mL of sodium acetate (10 mM) and 1 mL of fungal secretome. The absorbance change was monitored at 450 nm at 25°C. Enzyme activity was expressed as the amount of enzyme required to oxidize 1 µmol of guaiacol per min. The activity of the laccase presents in the *B. cinerea* secretome batch used in this study was evaluated at 0.9 U/L.

### 4.3 NMR analysis

NMR spectroscopic data were recorded on a Bruker Avance Neo 600 MHz NMR spectrometer equipped with a QCI 5 mm cryoprobe and a SampleJet automated sample changer (Bruker BioSpin, Rheinstetten, Germany). 1D and 2D NMR experiments (^1^H, COSY, ROESY, HMBC and HSQC) were recorded in DMSO-*d*_*6*_. Chemical shifts are reported in parts per million (δ) and coupling constants (*J*) in Hz. The residual DMSO-*d*_*6*_ signal (δ_H_ 2.50; δ_C_ 39.5) were used as internal standards for ^1^H and ^13^C NMR, respectively. All solvents used are HPLC or MS grade. Chemicals were purchased from Sigma-Aldrich unless otherwise stated.

### 4.4 Chromatographic separations

Reactions and fractions were monitored on a Waters Acquity ultra-high-performance liquid chromatography (UHPLC) system equipped with three detectors: a photodiode array (PDA), an evaporative light-scattering detector (ELSD) and a single quadrupole heated electrospray ionization (MS) detector, globally referred to as UHPLC-PDA-ELSD-MS (Waters, Milford, MA, United States). Separations were performed on an Acquity UPLC BEH C_18_ column (50 × 2.1 mm i.d., 1.7 µm; Waters) at 0.6 mL/min, 40°C with H_2_O (A) and MeCN (B) as solvents, both containing 0.1% formic acid (FA). The generic gradient used was from 5% to 100% MeCN in 7 min, followed by 1 min at 100% MeCN and 2 min of re-equilibration at 5% MeCN. The ESI parameters were: cone voltage 15 V, capillary voltage 800 V, probe temperature 600°C and source temperature 120°C. The acquisition was performed in positive or negative ionization mode with the *m/z* range set at 150–1000 Da. The ELSD temperature was set at 45°C, with a gain value of 9. The PDA data were acquired in the range 190–500 nm with a resolution of 1.2 nm. The sampling rate was set to 20 points/s. The data were processed using MassLynx (Waters, Milford, MA, United States).

Semi-preparative separations were performed on a Shimadzu HPLC-UV system equipped with an LC-20 A pump module, an SPD-20 A UV/Vis detector, a 7725I Rheodyne valve and an FRC-40 fraction collector (Shimadzu, Kyoto, Japan). Separations were performed on an Xbridge C_18_ column (250 × 19 mm i.d., 5 μm; Waters) at 17 mL/min, room temperature with H_2_O (A) and MeCN or MeOH (B) as solvents, both containing 0.1% FA. Samples were introduced using a dry-load method (Queiroz et al., 2019).

The flash separation was performed on a Büchi system equipped with a C-620 controller, two C-605 pump modules, a C-640 UV detector and a C-660 fraction collector. Separations were performed on a BGB Scorpius C_18_ C_1_8e-HP155 g 30 µm column (BGB Analytik, Böckten, Switzerland) with H_2_O (A) and MeOH (B) as solvents, both containing 0.1% FA.

The pure compounds were analyzed on a Waters Acquity UHPLC system equipped with a Q-Exactive Focus mass spectrometer (Thermo Scientific, Bremen, Germany), using heated electrospray ionization source (HESI-II). The chromatographic separation was carried out on an Acquity UPLC BEH C_18_ column (50 × 2.1 mm i.d., 1.7 μm; Waters) at 0.6 mL/min, 40°C with H_2_O (A) and MeCN (B) both containing 0.1% FA as solvents. The gradient was carried out as follows: 5%–100% B in 7 min, 1 min at 100% B, and a reequilibration step at 5% B in 2 min. The ionization parameters were the same as reported by Rutz et al. (2019).

### 4.5 Esterification of ferulic acid

The procedure was based on Yue *et al.* article (Yue et al., 2017). 1.00 g of ferulic acid (**1**) was solubilized in 50 mL of ethanol and 1 mL of acetyl chloride in a 100 mL round-bottomed flask. The reaction was stirred for 48 h at room temperature. The total consumption of the starting material was controlled by UHPLC-PDA-ELSD-MS monitoring. The solvent was evaporated using a rotary evaporator. 30 mL of EtOH was added 3 times and evaporated to remove traces of acetic acid, yielding 1.10 g (96% yield) of ethyl ferulate (**2**) pure enough without further purification.

Ethyl ferulate **(2)**: ^1^H NMR (DMSO-*d*_6_, 600 MHz) δ 1.25 (3H, t, *J* = 7.1 Hz, H_3_-11), 3.81 (3H, s, 3-OCH_3_), 4.16 (2H, q, *J* = 7.1 Hz, H_2_-10), 6.47 (1H, d, *J* = 15.9 Hz, H-8), 6.79 (1H, d, *J* = 8.1 Hz, H-5), 7.11 (1H, dd, *J* = 8.1, 2.2 Hz, H-6), 7.32 (1H, d, *J* = 2.2 Hz, H-2), 7.54 (1H, d, *J* = 15.9 Hz, H-7), 9.58 (1H, s, 4-OH); ^13^C NMR (DMSO-*d*_6_, 151 MHz) δ 14.2 (CH_3_-11), 55.7 (3-OCH_3_), 59.7 (CH_2_-10), 111.2 (CH-2), 114.6 (CH-8), 115.5 (CH-5), 123.1 (CH-6), 125.6 (C-1), 144.9 (CH-7), 147.9 (C-4), 149.3 (C-3), 166.6 (C-9); HR-ESI/MS analysis: *m/z* 221.0825 [M-H]^-^, (calcd for C_12_H_13_O_4_^−^, 221.0819, ∆ = 2.7 ppm). SMILES: OC1 = CC = C(C=C1OC)/C=C/C(OCC) = O.

### 4.6 Enzymatic dimerization of ethyl ferulate

The biotransformation reaction was performed in a 1 L Schott bottle, starting from 1 g of ethyl ferulate (**2**). The substrate was first dissolved in 50 mL of acetone, then water was added (410 mL) and finally the secretome of *B. cinerea* was added (40 mL). This mixture was incubated for 48 h at room temperature in the dark with gentle magnetic stirring. After evaporation of the acetone with a rotary evaporator, the water suspension was extracted with 3 × 200 mL of EtOAc. The combined organic phases were dried to give approximately 900 mg of crude reaction mixture, which was analyzed by UHPLC-PDA-ELSD-MS. The mixture was found to contain a very major dimeric product (**3**, see Figure 2C for the chromatogram of the crude reaction mixture with ELSD detection).

8–5′-benzofuran-diethylferulate (**3**): UV (MeOH) λ_max_ 201, 224 (sh), 289 (sh), 325 nm; ^1^H NMR (DMSO-*d*_6_, 600 MHz) δ 1.25 (3H, t, *J* = 7.1 Hz, H_3_-11), 1.26 (3H, t, *J* = 7.1 Hz, H_3_-11′), 3.76 (3H, s, 3-OCH_3_), 3.85 (3H, s, 3′-OCH_3_), 4.19 (2H, q, *J* = 7.1 Hz, H_2_-10′), 4.21 (2H, m, H_2_-10), 4.51 (1H, d, *J* = 8.0 Hz, H-8), 5.91 (1H, d, *J* = 8.0 Hz, H-7), 6.55 (1H, d, *J* = 15.9 Hz, H-8′), 6.77 (1H, d, *J* = 8.1 Hz, H-5), 6.81 (1H, dd, *J* = 8.1, 1.9 Hz, H-6), 7.00 (1H, d, *J* = 1.9 Hz, H-2), 7.26 (1H, s, H-6′), 7.40 (1H, s, H-2′), 7.63 (1H, d, *J* = 15.9 Hz, H-7′), 9.16 (1H, s, 4′-OH); ^13^C NMR (DMSO-*d*_6_, 151 MHz) δ 14.1 (CH_3_-11), 14.3 (CH_3_-11′), 54.2 (CH-8), 55.7 (3-OCH_3_), 56.0 (3′-OCH_3_), 59.8 (CH_2_-10′), 61.4 (CH_2_-10), 87.3 (CH-7), 110.8 (CH-2), 112.4 (CH-2′), 115.3 (CH-5), 115.7 (CH-8′), 118.3 (CH-6′), 119.4 (CH-6), 126.3 (C-5′), 128.1 (C-1′), 129.8 (C-1), 144.3 (C-3′), 144.6 (CH-7′), 147.1 (C-4), 147.7 (C-3), 149.4 (C-4′), 166.5 (C-9′), 170.3 (C-9); HR-ESI/MS analysis: *m/z* 441.1566 [M-H]^-^, (calcd for C_24_H_25_O_8_^−^, 441.1555, ∆ = 2.5 ppm). SMILES: OC1 = C(OC)C=C(C2OC(C(OC) = CC(/C=C/C(OCC) = O) = C3) = C3C2C(OCC) = O)C=C1.

### 4.7 Saponification—decarboxylation

Two small scale tests were carried out to find the best conditions, based on Yue et al., 2017 and Ralph et al., 1994 articles. Method A: At 50°C: 12 mg of **3** were added to 10 mL of 0.35 M NaOH in a round-bottomed flask. The mixture was heated to 50°C and stirred. 100 μL samples were taken after 10 min, 2 h, 6 h, and 24 h. At the end of the reaction, the flask was placed in an ice bath and acidified to pH 1 with a 32% HCl solution. An insoluble precipitate formed, which was extracted with 3 × 20 mL of EtOAc. The final reaction mixture (ca. 10 mg) separation was optimized at the UHPLC scale and transferred to the semi-preparative HPLC scale, using a gradient from 15% MeCN to 50% MeCN in 60 min. Compounds **6** (0.4 mg, t_R_ = 44.5 min), **7** (0.8 mg, t_R_ = 11.3 min), **8** (0.6 mg, t_R_ = 15.7 min) and **9** (0.5 mg, t_R_ = 20.0 min) were collected and analyzed by both HRMS and NMR.

Method B: At reflux: 10 mg of **3** were added to 10 mL of 0.35 M NaOH in a round-bottomed flask equipped with a condenser. The mixture was refluxed (heating set up at 120°C) and stirred. Samples were taken after 10 min, 2 h, 6 h, and 22 h. At the end of the reaction, the flask was placed in an ice bath and acidified to pH 1 with a 32% HCl solution. An insoluble precipitate formed, which was extracted with 3 × 20 mL of EtOAc. UHPLC-PDA-ELSD-MS confirmed the recovery of poacic acid as the major compound, together with **8** as a minor compound. This procedure was repeated on a larger scale for 30 min, with about 800 mg of the previous crude reaction mixture containing **3**. The obtained mixture was purified by flash column chromatography on a C_18_ column, using a gradient from 45% MeOH to 55% MeOH for 40 min, followed by a step from 55% to 65% until 60 min. The separation afforded 150 mg of poacic acid (**4**) (24% yield, t_R_ = 30.0 min).

(2*E*)-3-[4-hydroxy-3-[(1*E*)-2-(4-hydroxy-3-methoxyphenyl)ethenyl]-5-methoxyphenyl]-2-propenoic acid = poacic acid (**4**): UV (MeOH) λ_max_ (log ε) 235 (sh) (4.26), 290 (4.16), 323 (4.24) nm. ^1^H NMR (DMSO-*d*_6_, 600 MHz) δ 3.83 (3H, s, 3′-OCH_3_), 3.87 (3H, s, 3-OCH_3_), 6.47 (1H, d, *J* = 15.9 Hz, H-8′), 6.75 (1H, d, *J* = 8.1 Hz, H-5), 6.97 (1H, dd, *J* = 8.1, 1.9 Hz, H-6), 7.12 (1H, d, *J* = 1.9 Hz, H-2), 7.21 (3H, m, H-2′, H-7, H-8), 7.52 (1H, s, H-6′), 7.53 (1H, d, *J* = 15.8 Hz, H-7′); ^13^C NMR (DMSO-*d*_6_, 151 MHz) δ 55.5 (3-OCH_3_), 56.1 (3′-OCH_3_), 109 (C-2′), 109.7 (C-2), 115.7 (C-5), 116.2 (C-8′), 119.4 (C-8), 119.6 (C-6′), 119.9 (C-6), 125.4 (C-1′), 129.4 (C-7), 130.5 (C-1), 144.7 (C-7′), 145.9 (C-4′), 146.5 (C-4), 147.8 (C-3), 148 (C-3′), 167.8 (C-9′). ESI(−)-HRMS *m/z* 341.1029 [M-H]^-^, (calcd for C_19_H_17_O_6_, 341.1025, Δ = 1.1 ppm). MS/MS spectrum: CCMSLIB00006718004. SMILES: OC(/C=C/C1 = CC(OC) = C(O)C (/C=C/C2 = CC = C(O)C(OC) = C2) = C1) = O.

*threo*-8,8-bis [8–5′-benzofuran di-ferulic acid] (**6**): UV (MeOH) λ_max_ (log ε) 232 (sh) (4.61), 289 (4.48), 323 (4.52) nm. ^1^H NMR (CD_3_OD, 600 MHz) δ 3.64 (6H, s, 3-OCH_3_), 3.98 (6H, s, 3′-OCH_3_), 4.07 (2H, d, *J* = 4 Hz, H-8), 5.44 (2H, d, *J* = 4 Hz, H-7), 6.23 (2H, d, *J* = 2 Hz, H-2), 6.33 (2H, d, *J* = 15.8 Hz, H-8′), 6.42 (2H, dd, *J* = 8.1, 2 Hz, H-6), 6.63 (2H, d, *J* = 8.1 Hz, H-5), 7.12 (2H, d, *J* = 1.5 Hz, H-6′), 7.23 (2H, d, *J* = 1.5 Hz, H-2′), 7.56 (2H, d, *J* = 15.8 Hz, H-7′); ^13^C NMR (CD_3_OD, 151 MHz) δ 56.2 (CH-8), 56.3 (3-OCH_3_), 56.9 (3′-OCH_3_), 88.3 (CH-7), 109.6 (CH-2), 113.8 (CH-2′), 116.1 (CH-5), 117.9 (CH-8′), 118.5 (CH-6), 119.4 (CH-6′), 130.2 (C-5′), 130.5 (C-1′), 134.3 (C-1), 145.8 (CH-7′), 146.2 (C-3′), 147.5 (C-4), 149 (C-3), 152.3 (C-4′), 171.3 (C-9′). ESI(−)-HRMS *m/z* 681.2005 [M-H]^-^, (calcd for C_38_H_33_O_12_, 681.1972, Δ = 4.8 ppm). MS/MS spectrum: CCMSLIB00006717993. SMILES: OC(/C=C/C1 = CC(OC) = C(O[C@@](C2 = CC(OC) = C(O)C=C2)([H])[C@@]3 ([H])[C@@]4 ([H])[C@@](C5 = CC(OC) = C(O)C=C5)([H])OC6 = C(OC)C=C (/C=C/C(O) = O)C=C64)C3 = C1) = O.

8–5′-benzofuran-diferulic acid **(7)**: ^1^H NMR (CD_3_OD, 600 MHz) δ 3.83 (3H, s, 3-OCH_3_), 3.91 (3H, s, 3′OCH_3_), 4.31 (1H, d, *J* = 7.6 Hz, H-8), 6.02 (1H, d, *J* = 7.6 Hz, H-7), 6.36 (1H, d, *J* = 15.8 Hz, H-8′), 6.8 (1H, d, *J* = 8.1 Hz, H-5), 6.85 (2H, dd, *J* = 8.1, 2 Hz, H-6), 6.96 (1H, d, *J* = 2 Hz, H-2), 7.2 (1H, d, *J* = 1.5 Hz, H-2′), 7.27 (1H, d, *J* = 1.5 Hz, H-6′), 7.63 (1H, d, *J* = 15.8 Hz, H-7′); ^13^C NMR (CD_3_OD, 151 MHz) δ 56.4 (3-OCH_3_), 56.8 (3′-OCH_3_), 56.9 (CH-8), 89.1 (CH-7), 110.6 (CH-2), 113.9 (CH-2′), 116.3 (CH-5), 116.9 (CH-8′), 119.2 (CH-6′), 119.9 (CH-6), 128.2 (C-5′), 130 (C-1′), 132.9 (C-1), 146.1 (C-3′), 146.5 (CH-7′), 148.1 (C-4), 149.3 (C-3), 151.4 (C-4′), 170.8 (C-9′), 174.0 (C-9). ESI(−)-HRMS *m/z* 385.0933 [M-H]^-^, (calcd for C_20_H_17_O_8_, 385.0923, Δ = 2.6 ppm). MS/MS spectrum: CCMSLIB00006717995. SMILES: OC(/C=C/C1 = CC2 = C(O[C@@](C3 = CC(OC) = C(O)C=C3)([H])[C@@]2 ([H])C(O) = O)C(OC) = C1) = O.

(*Z*)-2-(5-((*E*)-2-carboxyvinyl)-2-hydroxy-3-methoxyphenyl)-3-(4-hydroxy-3-methoxyphenyl)acrylic acid **(8)**: ^1^H NMR (DMSO-*d*_6_, 600 MHz) δ 3.35 (3H, s, 3′-OCH_3_), 3.88 (3H, s, 3-OCH_3_), 6.37 (1H, d, *J* = 15.8 Hz, H-8), 6.62 (1H, d, *J* = 2.1 Hz, H-2′), 6.64 (1H, d, *J* = 8.2 Hz, H-5′), 6.72 (1H, dd, *J* = 8.2, 2.1 Hz, H-6′), 6.90 (1H, d, *J* = 2.0 Hz, H-6), 7.34 (1H, d, *J* = 2.0 Hz, H-2), 7.47 (1H, d, *J* = 15.8 Hz, H-7), 7.63 (1H, s, H-7′), 9.14 (1H, s, 4-OH), 9.44 (1H, s, 4′-OH), 12.16 (2H, s, COOH); ^13^C NMR (DMSO-*d*_6_, 151 MHz) δ 54.6 (3′-OCH_3_), 56.1 (3-OCH_3_), 109.7 (CH-2), 112.8 (CH-2′), 115.2 (CH-5′), 115.9 (CH-8), 124.4 (CH-6), 124.6 (C-5), 125.0 (CH-6′), 125.7 (C-1), 125.9 (C-1′), 125.9 (C-8′), 139.8 (CH-7′), 144.3 (CH-7), 146.8 (C-4), 147.0 (C-3′), 148.1 (C-4′), 148.1 (C-3), 167.9 (C-9), 168.4 (C-9′); HR-ESI/MS analysis: *m/z* 385.0936 [M-H]^-^, (calcd for C_20_H_17_O_8_^−^, 385.0929, ∆ = 1.8 ppm). SMILES: O=C(O)/C=C/C1 = CC(/C(C(O) = O) = C/C2 = CC(OC) = C(O)C=C2) = C(O)C(OC) = C1.

(*E*)-3-(3-formyl-4-hydroxy-5-methoxyphenyl)acrylic acid **(9):** ^1^H NMR (DMSO-*d*_6_, 600 MHz) δ 3.92 (3H, s, 3-OCH_3_), 6.51 (1H, d, *J* = 15.9 Hz, H-8), 7.48 (1H, d, *J* = 2.0 Hz, H-6), 7.54 (1H, d, *J* = 15.9 Hz, H-7), 7.61 (1H, d, *J* = 2.0 Hz, H-2), 10.27 (1H, s, CO-H), 10.69 (1H, s, 4-OH), 12.27 (1H, s, COOH); ^13^C NMR (DMSO-*d*_6_, 151 MHz) δ 56.4 (3-OCH_3_, 115.0 (CH-2), 117.8 (CH-8), 121.5 (CH-6), 122.3 (C-5), 125.7 (C-1), 143.3 (CH-7), 148.9 (C-3), 152.6 (C-4), 167.7 (C-9), 191.0 (CO-H); HR-ESI/MS analysis: *m/z* 221.0463 [M-H]^-^, (calcd for C_11_H_9_O_5_^−^, 221.0455, ∆ = 3.4 ppm). SMILES: O=C(O)/C=C/C1 = CC(C=O) = C(O)C(OC) = C1.

### 4.8 Enzymatic dimerization of poacic acid

The biotransformation reaction was carried out in a 25 mL Schott bottle starting from 20 mg of poacic acid (**4**). The substrate was first dissolved in 2 mL of acetone, then water was added (17.6 mL) and finally the secretome of *B. cinerea* (400 μL, 2%) was added. This mixture was incubated for 24 h at room temperature in the dark with gentle magnetic stirring. After total evaporation of the solvent with a rotary evaporator, the dry precipitate was resuspended with MeOH and filtered on Büchner to obtain approximately 18 mg of crude reaction mixture, which was analyzed by UHPLC-PDA-ELSD-MS. The two dimers obtained were further separated by semi-preparative HPLC-UV, using a gradient from 30% to 60% MeOH in 30 min, yielding **5** (3.1 mg, t_R_ = 14.2 min) and **6** (3.0 mg, t_R_ = 15.2 min). The two compounds obtained were analyzed by UHPLC-PDA-ELSD-MS and NMR to confirm their identity.

*meso*-8,8-bis [8–5′-benzofuran di-ferulic acid] (**5**): UV (MeOH) λ_max_ (log ε) 234 (sh) (4.53), 290 (4.39), 324 (4.44) nm. ^1^H NMR (CD_3_OD, 600 MHz) δ 3.78 (6H, s, 3-OCH_3_), 3.91 (6H, s, 3′-OCH_3_), 4.06 (2H, d, *J* = 5.6 Hz, H-8), 5.45 (2H, d, *J* = 5.6 Hz, H-7), 6.2 (2H, d, *J* = 15.9 Hz, H-8′), 6.68 (2H, d, *J* = 1.6 Hz, H-6′), 6.78 (2H, dd, *J* = 8.1, 1.8 Hz, H-6), 6.8 (2H, d, *J* = 8.1 Hz, H-5), 6.82 (2H, d, *J* = 1.8 Hz, H-2), 7.2 (2H, d, *J* = 1.6 Hz, H-2′), 7.54 (2H, d, *J* = 15.9 Hz, H-7′); ^13^C NMR (CD_3_OD, 151 MHz) δ 54.4 (CH-8), 56.4 (3-OCH_3_), 56.8 (3′-OCH_3_), 89.5 (CH-7), 110.9 (CH-2), 114.1 (CH-2′), 116.5 (CH-5), 117.2 (CH-8′), 118.7 (CH-6′), 120.3 (CH-6), 130.2 (C-1′), 130.4 (C-5′), 133.3 (C-1), 146.1 (C-3′), 146.3 (CH-7′), 148.3 (C-4), 149.3 (C-3), 152.3 (C-4′), 171 (C-9′). ESI(−)-HRMS *m/z* 681.2004 [M-H]^-^, (calcd for C_38_H_33_O_12_, 681.1972, Δ = 4.7 ppm). MS/MS spectrum: CCMSLIB00006717998. SMILES: OC(/C=C/C1 = CC(OC) = C(O[C@@](C2 = CC(OC) = C(O)C=C2)([H])[C@@]3 ([H])[C@]4 ([H])[C@](C5 = CC(OC) = C(O)C=C5)([H])OC6 = C(OC)C=C (/C=C/C(O) = O)C=C64)C3 = C1) = O.

### 4.9 Biotransformation of phenolic compounds

Biotransformation reactions were performed in 2 mL Eppendorf tubes with 4.4 µmol of the different starting compounds: Phenol, 4-*O*-methyl-resveratrol (AmBeed, Arlington Heights, IL, United States), resveratrol (Biopurify, Chengdu, China), *trans*-δ-viniferin (isolated according to the method described in (Righi et al., 2020; Huber et al., 2022a)), *trans*-ε-viniferin (isolated from *V. vinifera* canes) and dihydro-resveratrol (obtained by hydrogenation of resveratrol, see section below). For each substrate, three samples were prepared: two reactions with 5% and 20% secretome of *B. cinerea*, respectively, and a negative control without secretome. The total volume was fixed at 1 mL. The required volume of water was added first, followed by the secretome (50 µL for 5%, 200 µL for 20%) and finally 100 µL of the stock solution loaded with substrate (at 44 mM). The detailed list of volumes is given in Supplementary Table S2. After preparation, samples were incubated at room temperature with gentle shaking in the dark. After 48 h, the acetone was removed under vacuum in a centrifugal evaporator and the water was extracted with 2 × 500 µL of ethyl acetate. The combined organic phases were dried under a nitrogen stream, the samples were solubilized again in 200 µL of MeOH and analyzed by UHPLC-PDA-ELSD-MS.

### 4.10 Resveratrol hydrogenation

Resveratrol was solubilized in a 1:1 mixture of MeOH and EtOAc under nitrogen atmosphere at 0.025 M. Then, 0.1 equivalent of the catalyst Pd/C 10% was added and the system was purged 3 times with vacuum and filled back with H_2_ atmosphere (simple balloon pressure). The mixture was stirred at room temperature overnight (approx. 15 h). The crude reaction mixture was filtered on a celite^®^ pad (1 cm) and washed with 4 × 20 mL of a 1:1 MeOH/EtOAc mixture. The solvent was evaporated *in vacuo* to give dihydro-resveratrol (**10**) pure enough without further purification.

dihydro-resveratrol **(10)**: UV (MeOH) λmax (log ε) 230 (sh) (4.14), 280 (3.46) nm. ^1^H NMR (DMSO-*d*_6_, 600 MHz) δ 2.60 (2H, m, H_2_-8), 2.67 (2H, m, H_2_-7), 6.01 (1H, t, *J* = 2.2 Hz, H-12), 6.05 (2H, d, *J* = 2.2 Hz, H-10, H-14), 6.64 (2H, d, *J* = 8.4 Hz, H-3, H-5), 6.98 (2H, *J* = 8.4 Hz, H-2, H-6), 9.01 (2H, s, 11-OH, 13-OH), 9.10 (1H, s, 4-OH); ^13^C NMR (DMSO-*d*_6_, 151 MHz) δ 36.1 (CH_2_-7), 37.6 (CH_2_-8), 100.1 (CH-12), 106.4 (CH-10, CH-14), 114.9 (CH-3, CH-5), 129.1 (CH-2, CH-6), 131.7 (C-1), 143.6 (C-9), 155.3 (C-4), 158.2 (C-11, C-13); HR-ESI/MS analysis: *m/z* 229.0864 [M-H]^-^, (calcd for C_14_H_13_O_3_^−^, 229.0870, ∆ = 2.7 ppm). Supplementary Figures S6–S11. SMILES: OC1 = CC(O) = CC(CCC2 = CC = C(O)C=C2) = C1.

### 4.11 Enzymatic dimerization of dihydro-resveratrol

The biotransformation reaction was performed in a 100 mL Schott bottle, starting from 40 mg of dihydro-resveratrol (**10**). The substrate was first dissolved in 4 mL of acetone, then water was added (34 mL) and finally the secretome of *B. cinerea* was added (2 mL). This mixture was incubated for 48 h at room temperature in the dark with gentle magnetic stirring. After total evaporation of the solvent with a rotary evaporator, the dry deposit was resuspended with MeOH and transferred in a 10 mL vial and dried under nitrogen stream to give 34.8 mg. The crude reaction mixture was analyzed by UHPLC-PDA-ELSD-MS and matched the profile obtained at small scale (Supplementary Figure S4G). The separation was optimized at the UHPLC scale and transferred to the semi-preparative HPLC scale. The whole amount was injected using a gradient from 25% to 40% MeCN in 60 min. Compounds **11** (0.2 mg, t_R_ = 15.5 min), **12** (0.3 mg, t_R_ = 19.0 min), **13** (0.2 mg, t_R_ = 30.7 min)**, 14** (0.2 mg, t_R_ = 38.0 min)**, 15** (0.4 mg, t_R_ = 31.5 min) and **16** (0.2 mg, t_R_ = 32.5 min) were collected and analyzed by both HRMS and NMR.

10–10′-dihydro-resveratrol dimer **(11)**: UV (MeOH) λmax (log ε) 229 (sh) (4.27), 285 (3.67) nm. ^1^H NMR (DMSO-*d*_6_, 600 MHz) δ 2.29 (4H, m, H_2_-8, H_2_-8′), 2.39 (2H, overlapped, H-7b, H-7’b), 2.52 (2H, overlapped, H-7a, H-7’a), 6.18 (2H, d, *J* = 2.3 Hz, H-14, H-14′), 6.20 (2H, d, *J* = 2.3 Hz, H-12, H-12′), 6.56 (4H, d, *J* = 8.5 Hz, H-3, H-5, H-3′, H-5′), 6.73 (4H, d, *J* = 8.5 Hz, H-2, H-6, H-2′, H-6′), 8.52 (2H, s, 11-OH, 11′-OH), 8.91 (2H, s, 13-OH, 13′-OH), 9.04 (2H, s, 4-OH, 4′-OH); ^13^C NMR (DMSO-*d*_6_, 151 MHz) δ 35.4 (CH_2_-7, CH_2_-7′), 36.3 (CH_2_-8, CH_2_-8′), 100.3 (CH-12, CH-12′), 106.6 (CH-14, CH-14′), 114.6 (C-10, C-10′), 114.9 (CH-3, CH-5, CH-3′, CH-5′), 128.9 (CH-2, CH-6, CH-2′, CH-6′), 132.4 (C-1, C-1′), 155.3 (C-4, C-4′), 156.4 (C-11, C-13, C-11′, C-13′); HR-ESI/MS analysis: *m/z* 457.1652 [M-H]^-^, (calcd for C_28_H_25_O_6_^−^, 457.1657, ∆ = 1.0 ppm). MS/MS spectrum: CCMSLIB00011431687. NMR spectra: Supplementary Figures S12–S16. SMILES: OC1 = CC(O) = C(C(C(O) = CC(O) = C2) = C2CCC3 = CC = C(O)C=C3)C(CCC4 = CC = C(O)C=C4) = C1.

3–10′-dihydro-resveratrol dimer **(12)**: UV (MeOH) λmax (log ε) 228 (sh) (4.44), 284 (3.82) nm. ^1^H NMR (DMSO-*d*_6_, 600 MHz) δ 2.32 (1H, m, H-8’b), 2.41 (2H, m, H-7’b, H-8’a), 2.47 (1H, overlapped, H-7’a), 2.60 (2H, overlapped, H_2_-8), 2.68 (2H, m, H_2_-7), 6.02 (1H, t, *J* = 2.2 Hz, H-12), 6.09 (2H, d, *J* = 2.2 Hz, H-10, H-14), 6.15 (1H, d, *J* = 2.4 Hz, H-14′), 6.19 (1H, d, *J* = 2.4 Hz, H-12′), 6.54 (2H, d, *J* = 8.4 Hz, H-3′, H-5′), 6.67 (2H, d, *J* = 8.4 Hz, H-2′, H-6′), 6.75 (1H, d, *J* = 8.1 Hz, H-5), 6.80 (1H, d, *J* = 2.3 Hz, H-2), 6.97 (1H, dd, *J* = 8.1, 2.3 Hz, H-6), 8.63 (1H, s, 11′-OH), 8.96 (1H, s, 13′-OH), 9.03 (2H, s, 11-OH, 13-OH), 9.04 (1H, s, 4-OH); ^13^C NMR (DMSO-*d*_6_, 151 MHz) δ 35.8 (CH_2_-7′), 36.5 (CH_2_-7, CH_2_-8′), 38.1 (CH_2_-8), 100.1 (CH-12), 100.3 (CH-12′), 106.2 (CH-10, CH-14), 106.6 (CH-14′), 114.9 (CH-3′, CH-5′), 115.1 (CH-5), 116.7 (C-10′), 124.3 (C-3), 127.4 (CH-6), 128.9 (CH-2′, CH-6′), 131.5 (C-1), 132.1 (CH-2), 132.4 (C-1′), 142.3 (C-9′), 144.1 (C-9), 153.3 (C-4), 155.1 (C-4′), 155.8 (C-11′), 156.5 (C-13′), 158.2 (C-11, C-13); HR-ESI/MS analysis: *m/z* 457.1654 [M-H]^-^, (calcd for C_28_H_25_O_6_^−^, 457.1657, ∆ = 0.6 ppm). MS/MS spectrum: CCMSLIB00011431688. NMR spectra: Supplementary Figures S17–S21. SMILES: OC1 = CC(O) = C(C2 = CC(CCC3 = CC(O) = CC(O) = C3) = CC = C2O)C(CCC4 = CC = C(O)C=C4) = C1.

3–3′-dihydro-resveratrol dimer **(13)**: UV (MeOH) λmax (log ε) 226 (sh) (4.32), 289 (3.61) nm. ^1^H NMR (DMSO-*d*_6_, 600 MHz) δ 2.64 (4H, m, H_2_-8, H_2_-8′), 2.71 (4H, m, H_2_-7, H_2_-7′), 6.03 (2H, t, *J* = 2.1 Hz, H-12, H-12′), 6.09 (4H, d, *J* = 2.1 Hz, H-10, H-14, H-10′, H-14′), 6.74 (2H, brs, H-5, H-5′), 6.94 (2H, d, *J* = 2.2 Hz, H-2, H-2′), 6.96 (2H, d, *J* = 7.8 Hz, H-6, H-6′), 9.02 (4H, s, 11-OH, 13-OH, 11′-OH, 13′-OH); ^13^C NMR (DMSO-*d*_6_, 151 MHz) δ 36.3 (CH_2_-7, CH_2_-7′), 37.9 (CH_2_-8, CH_2_-8′), 100.1 (CH-12, CH-12′), 106.4 (CH-10, CH-14, CH-10′, CH-14′), 115.9 (CH-5, CH-5′), 126.2 (C-3, C-3′), 127.7 (CH-6, CH-6′), 131.0 (CH-2, CH-2′), 143.9 (C-9, C-9′), 158.2 (C-11, C-13, C-11′, C-13′); HR-ESI/MS analysis: *m/z* 457.1654 [M-H]^-^, (calcd for C_28_H_25_O_6_^−^, 457.1657, ∆ = 0.6 ppm). MS/MS spectrum: CCMSLIB00011431689. NMR spectra: Supplementary Figures S22–S26. SMILES: OC1 = CC(O) = CC(CCC2 = CC = C(O)C(C3 = C(O)C=CC(CCC4 = CC(O) = CC(O) = C4) = C3) = C2) = C1.

4-*O*-10′-dihydro-resveratrol dimer **(14)**: UV (MeOH) λmax (log ε) 229 (sh) (4.51), 283 (3.86) nm. ^1^H NMR (DMSO-*d*_6_, 600 MHz) δ 2.47 (2H, overlapped, H_2_-8′), 2.55 (2H, overlapped, H_2_-7′), 2.63 (2H, overlapped, H_2_-8), 2.71 (2H, m, H_2_-7), 6.02 (1H, t, *J* = 2.2 Hz, H-12), 6.08 (2H, d, *J* = 2.2 Hz, H-10, H-14), 6.13 (1H, d, *J* = 2.8 Hz, H-14′), 6.26 (1H, d, *J* = 2.8 Hz, H-12′), 6.59 (2H, d, *J* = 8.5 Hz, H-3′, H-5′), 6.66 (2H, d, *J* = 8.6 Hz, H-3, H-5), 6.82 (2H, d, *J* = 8.5 Hz, H-2′, H-6′), 7.11 (2H, d, *J* = 8.6 Hz, H-2, H-6), 9.03 (2H, s, 11-OH, 13-OH), 9.07 (1H, s, 13′-OH), 9.09 (1H, s, 4′-OH), 9.18 (1H, s, 11′-OH); ^13^C NMR (DMSO-*d*_6_, 151 MHz) δ 32.4 (CH_2_-8′), 35.1 (CH_2_-7′), 36.1 (CH_2_-7), 37.7 (CH_2_-8), 100.1 (CH-12), 101.8 (CH-12′), 106.2 (CH-10, CH-14), 106.8 (CH-14′), 128.9 (CH-2′, CH-6′), 129.1 (CH-2, CH-6), 131.7 (C-1′), 132.4 (C-10′), 134.2 (C-1), 136.0 (C-9′), 143.6 (C-9), 150.6 (C-11′), 155.0 (C-13′), 155.3 (C-4′), 156.9 (C-4), 158.2 (C-11, C-13); HR-ESI/MS analysis: *m/z* 457.1653 [M-H]^-^, (calcd for C_28_H_25_O_6_^−^, 457.1657, ∆ = 0.8 ppm). MS/MS spectrum: CCMSLIB00011431690. NMR spectra: Supplementary Figures S27–S31. SMILES: OC1 = CC(O) = CC(CCC2 = CC = C (OC(C(O) = CC(O) = C3) = C3CCC4 = CC = C(O)C=C4)C=C2) = C1.

8,9b-bis(3,5-dihydroxyphenethyl)-4a, 9b-dihydrodibenzo [*b*,*d*]furan-3(4*H*)-one (**15,** in mixture with **16**): ^1^H NMR (DMSO-*d*_6_, 600 MHz) δ 2.10 (1H, td, *J* = 13.5, 12.3, 5.6 Hz, H-7’b), 2.27 (1H, td, *J* = 13.5, 12.7, 4.9 Hz, H-7’a), 2.44 (2H, overlapped, H_2_-8′), 2.64 (2H, t, *J* = 8.1 Hz, H_2_-8), 2.74 (2H, t, *J* = 8.1 Hz, H_2_-7), 2.79 (1H, dd, *J* = 17.6, 2.7 Hz, H-5’b), 3.02 (1H, dd, *J* = 17.6, 4.1 Hz, H-5’a), 4.99 (1H, dt, *J* = 4.1, 2.5, 1.9 Hz, H-6′), 5.93 (1H, d, *J* = 10.2 Hz, H-3′), 6.03 (1H, t, *J* = 2.2 Hz, H-12), 6.04 (1H, t, *J* = 2.2 Hz, H-12′), 6.07 (2H, d, *J* = 2.2 Hz, H-10, H-14), 6.10 (2H, d, *J* = 2.2 Hz, H-10′, H-14′), 6.67 (1H, dd, *J* = 10.2, 1.9 Hz, H-2′), 6.70 (1H, d, *J* = 8.2 Hz, H-5), 7.01 (1H, dd, *J* = 8.2, 1.9 Hz, H-6), 7.27 (1H, d, *J* = 1.9 Hz, H-2), 9.04 (2H, s, 11-OH, 13-OH), 9.07 (2H, s, 11′-OH, 13′-OH); ^13^C NMR (DMSO-*d*_6_, 151 MHz) δ 30.4 (CH_2_-8′), 36.5 (CH_2_-7′), 36.6 (CH_2_-7), 37.9 (CH_2_-8), 38.9 (CH_2_-5′), 48.4 (C-1′), 84.3 (CH-6′), 100.2 (C-12), 100.3 (C-12′), 106.4 (C-10, C-14, C-10′, C-14′), 109.2 (CH-5), 123.7 (CH-2), 126.3 (CH-3′), 128.7 (CH-6), 131.7 (C-1), 134.9 (C-3), 143.5 (C-9′), 143.6 (C-9), 149.8 (C-2′), 156.4 (C-4), 158.2 (C-11, C-13), 158.3 (C-11′, C-13′), 195.5 (C-4′); HR-ESI/MS analysis: *m/z* 457.1653 [M-H]^-^, (calcd for C_28_H_25_O_6_^−^, 457.1657, ∆ = 0.8 ppm). MS/MS spectrum: CCMSLIB00011431691. NMR spectra: Supplementary Figures S32–S36. SMILES: OC1 = CC(O) = CC(CCC2 = CC3 = C (OC(CC(C=C4) = O)C43CCC5 = CC(O) = CC(O) = C5)C=C2) = C1.

11-(3,5-dihydroxyphenethyl)-2,4-dihydroxy-4b,5,7,7a, 13,14-hexahydro-6*H*-phenanthro [1,10a-*b*]benzofuran-6-one **(16)**: UV (MeOH) λmax (log ε) 230 (sh) (4.30), 286 (3.84) nm. ^1^H NMR (DMSO-*d*_6_, 600 MHz) δ 1.90 (1H, dt, *J* = 12.7, 6.4 Hz, H-7’b), 2.07 (1H, dt, *J* = 12.7, 6.4 Hz, H-7’a), 2.32 (1H, dd, *J* = 17.0, 3.8 Hz, H-3’b), 2.45 (1H, dd, *J* = 16.7, 4.4 Hz, H-5’b), 2.49 (1H, overlapped, H-8b), 2.53 (1H, overlapped, H-8a), 2.63 (2H, overlapped, H_2_-7), 2.69 (1H, dd, *J* = 17.0, 9.1 Hz, H-3’a), 2.72 (1H, dd, *J* = 16.7, 7.1 Hz, H-5’a), 2.72 (2H, overlapped, H_2_-8′), 3.23 (1H, dd, *J* = 9.1, 3.8 Hz, H-2′), 4.80 (1H, dd, *J* = 7.1, 4.4 Hz, H-6′), 6.00 (3H, s, H-10, H-12, H-14), 6.08 (1H, d, *J* = 2.4 Hz, H-10′), 6.16 (1H, d, *J* = 2.4 Hz, H-12′), 6.71 (1H, d, *J* = 8.1 Hz, H-5), 6.75 (1H, d, *J* = 1.9 Hz, H-2), 6.99 (1H, dd, *J* = 8.1, 1.9 Hz, H-6), 9.00 (2H, s, 11-OH, 13-OH), 9.04 (1H, s, 11′-OH), 9.33 (1H, s, 13′-OH); ^13^C NMR (DMSO-*d*_6_, 151 MHz) δ 25.4 (CH_2_-8′), 30.1 (CH_2_-7′), 36.5 (CH_2_-7), 37.0 (CH-2′), 37.7 (CH_2_-8), 41.4 (CH_2_-3′), 42.3 (CH_2_-5′), 46.1 (C-1′), 86.7 (CH-6′), 100.1 (CH-12), 101.0 (CH-12′), 106.3 (CH-10′), 106.4 (CH-10, CH-14), 109.0 (CH-5), 114.2 (C-14′), 124.0 (CH-2), 128.4 (CH-6), 134.0 (C-1), 134.4 (C-3), 137.8 (C-9′), 143.4 (C-9), 156.2 (C-11′, C-13′), 156.4 (C-4), 158.2 (C-11, C-13), 208.8 (C-4′); HR-ESI/MS analysis: *m/z* 457.1653 [M-H]^-^, (calcd for C_28_H_25_O_6_^−^, 457.1657, ∆ = 0.8 ppm). MS/MS spectrum: CCMSLIB00011431692. NMR spectra: Supplementary Figures S37–S46. SMILES: OC1 = CC(O) = CC(CCC2 = CC3 = C (OC(C4)C53CCC6 = CC(O) = CC(O) = C6C5CC4 = O)C=C2) = C1.

## Data Availability

The raw data files for the NMR are available at the following link: https://doi.org/10.26037/yareta:26ty7islnfh33cwyok4fkqzvwe. The MS/MS spectrum of each isolated compound has its own accession number from CCMSLIB00011431687 to CCMSLIB00011431692 on the Global Natural Product Social Molecular Networking (GNPS) (accessed via: https://gnps.ucsd.edu/ProteoSAFe/static/gnps-splash.jsp).
