# Supplementary material for: Study of phenoxy radical couplings using the enzymatic secretome of Botrytis cinerea
Source: Front Chem. 2024 May 28;12:1390066. doi: 10.3389/fchem.2024.1390066 (PMC11165214; doi:10.3389/fchem.2024.1390066)
Supplement: Supplementary file 1 [file DataSheet1.PDF]

Supplementary Material

Study of phenoxy radical couplings using the enzymatic secretome of *Botrytis cinerea*

Robin Huber[1,2], Laurence Marcourt[1,2], Fabien Félix[1,2], Sébastien Tardy[1,2], Emilie Michellod[3], Leonardo Scapozza[1,2], Jean-Luc Wolfender[1,2], Katia Gindro[3†], Emerson F. Queiroz[1,2*†]

The first saponification – decarboxylation test (method A) was carried out at 50 °C in a 0.35 M NaOH solution. Samples were taken after 10 min, 2h, 6h and 22h (Figure S1). After 10 min, compound **7** (previously found in grass cell walls[1]) was obtained as an almost pure compound, corresponding to the simple hydrolysis (without decarboxylation). The expected decarboxylated poacic acid (**4**) was found at 2h and 6h, but only in small amount, and totally disappeared after 24h. Other peaks were detected and therefore isolated by semi-preparative HPLC-UV to better understand the reaction. Compound **8** (previously isolated in wheat plants[2]) was shown to have lost its dihydrofuran ring by elimination followed by ring opening, but without decarboxylation. Compound **9** is a monomer obtained by oxidative cleavage. Interestingly, a poacic acid dimer (**6**, described in the main article) was found upon prolonged reaction time (Figure S1).

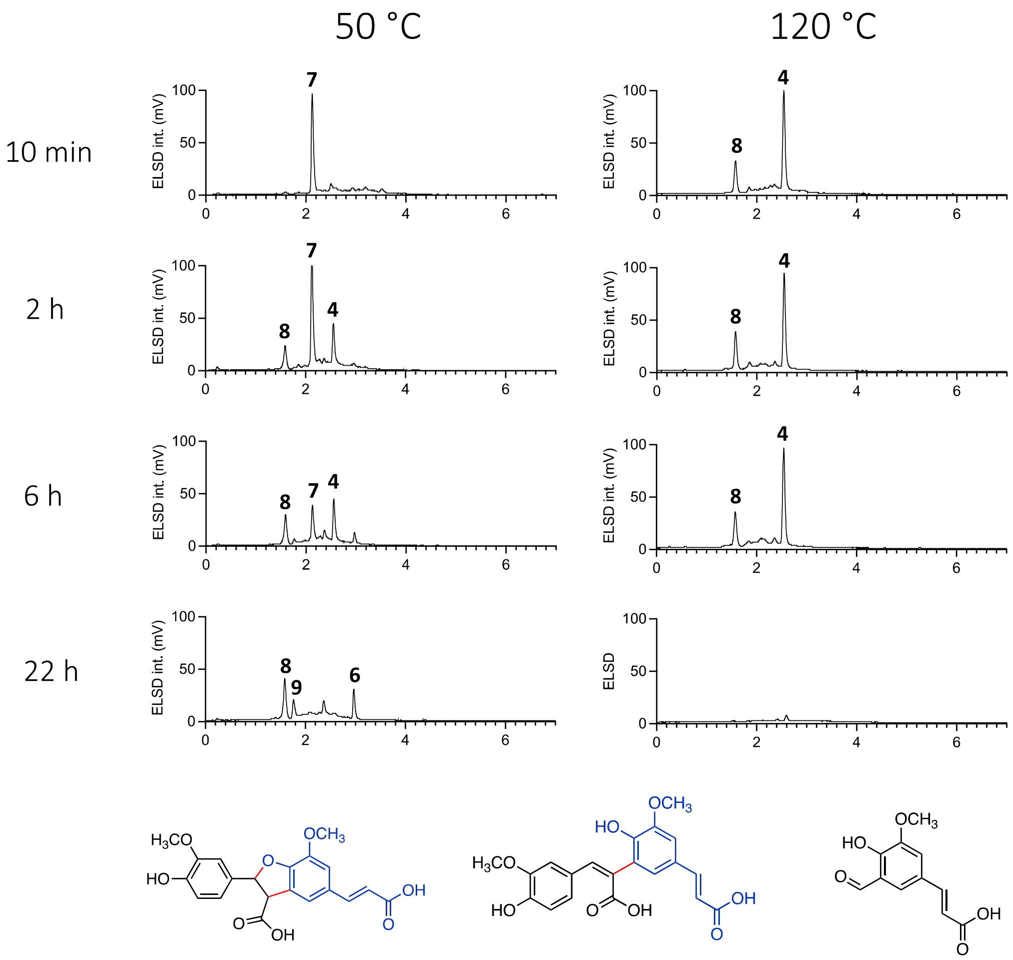

7 **8** **9**

Figure S1. Samples taken during hydrolysis at 50 and 120 °C and analyzed by UHPLC-PDA-ELSD-MS, with the ELSD detection displayed. Compounds **4**, **6**, **7**, **8** and **9** were isolated by semi-preparative HPLC and compared with previously isolated compounds or *de novo* elucidated by NMR. Optimal condition for obtaining poacic acid (**4**) is hydrolysis at 120 °C between 10 min and 6 h.

---

[1] Ralph J, Quideau S, Grabber JH, Hatfield RD. Identification and synthesis of new ferulic acid dehydrodimers present in grass cell walls. J Chem Soc, Perkin Trans 1. 1994; 3485. doi:10.1039/p19940003485

[2] Callipo L, Cavaliere C, Fuscoletti V, Gubbiotti R, Samperi R, Laganà A. Phenilpropanoate identification in young wheat plants by liquid chromatography/tandem mass spectrometry: monomeric and dimeric compounds. J Mass Spectrom. 2010;45: 1026–1040. doi:10.1002/jms.1800

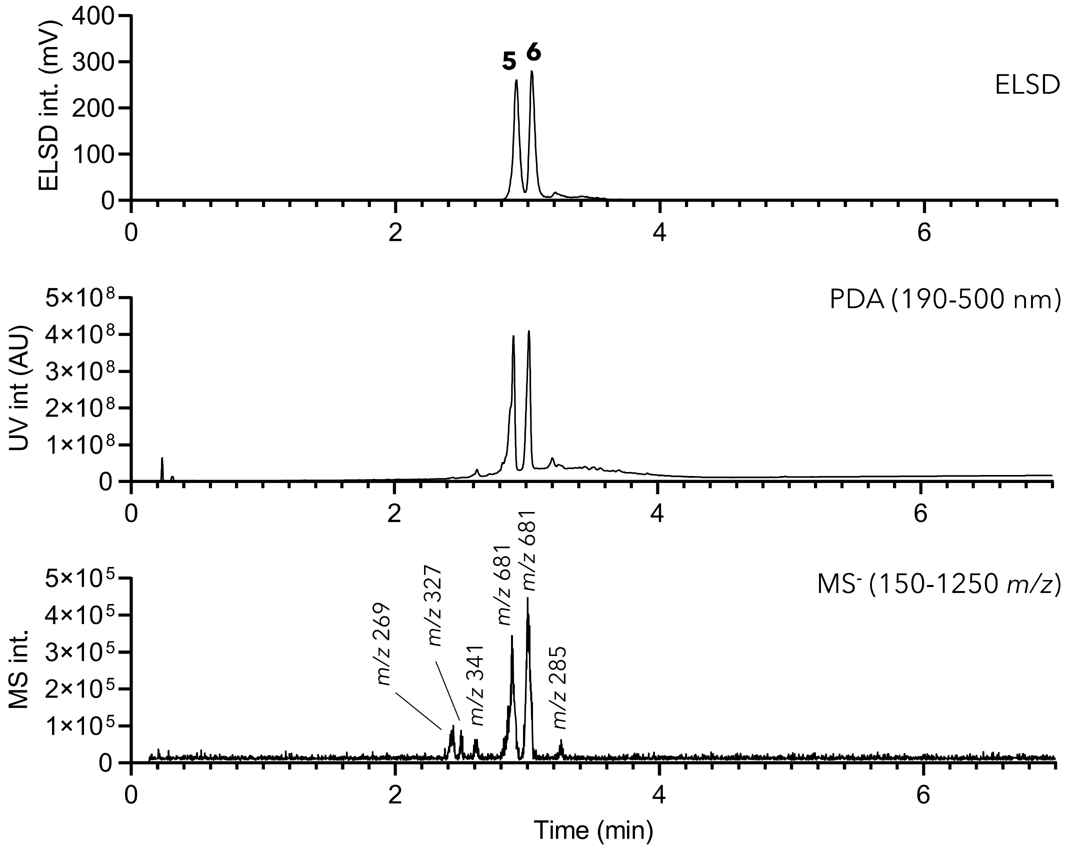

Figure S2. UHPLC-PDA-ELSD-MS analysis of the crude reaction mixture of the poacic acid dimerization. The ELSD detector highlights the presence of two major compounds in the mixture (**5** and **6**), further confirmed by the absence of other strong signals with UV (190-500 nm) and MS (150-1250 *m/z*) detection. The annotated values on the MS chromatogram are the top scan ion for each signal.

Compounds **5** and **6** were obtained by tetramerization of ferulic acid in one of our previous works[3]. 0.7 mg of compounds **5** (numbered **13** in Huber *et al*. 2022) and 0.6 mg of **6** (numbered **14** in Huber *et al*. 2022) were isolated in our previous article, starting from 80 mg of ferulic acid. In the present article, 3.1 mg of **5** and 3.0 mg of **6** were isolated, starting from 18.0 mg of poacic acid (**4**).

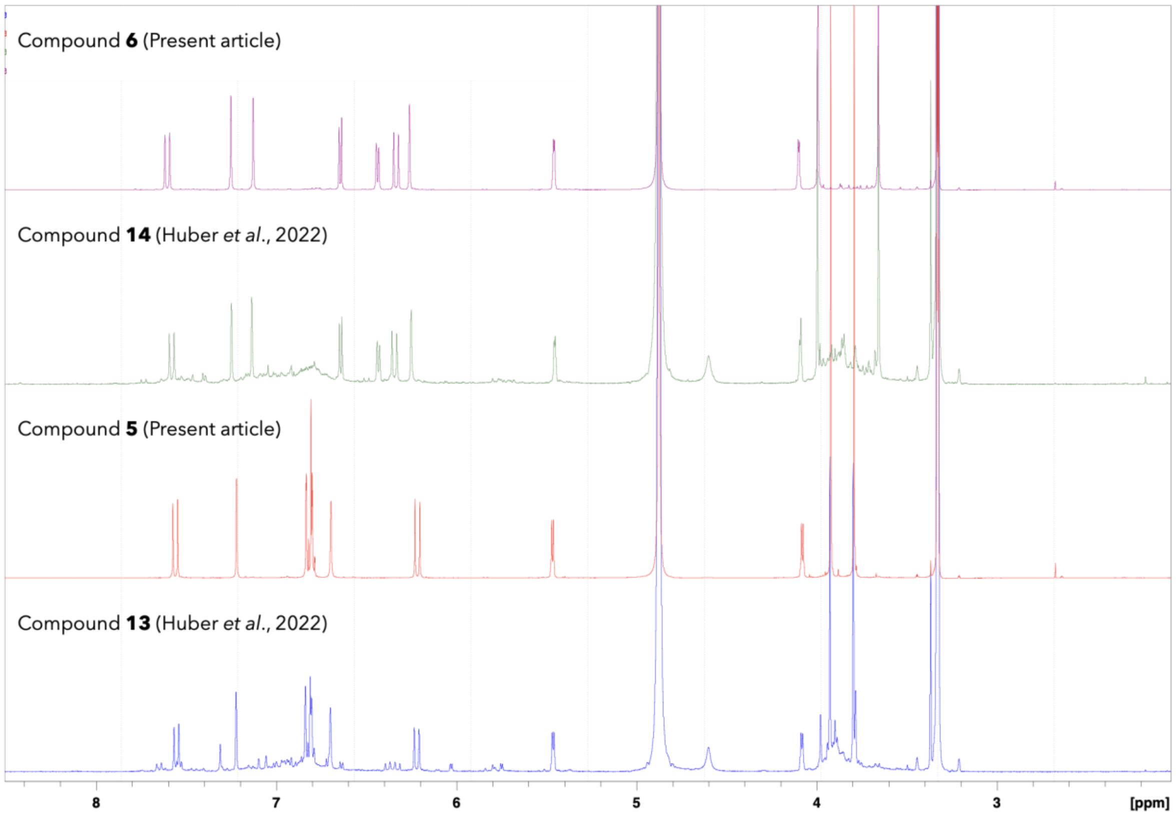

Figure S3. Purity comparison of compounds **5** and **6** obtained in this article by dimerization of poacic acid (**4**) with the same compounds isolated in our previous publication from tetramerization of ferulic acid (**13** and **14**)[3]

[3]Huber R, Marcourt L, Koval A, Schnee S, Righi D, Michellod E, *et al*. Chemoenzymatic synthesis of complex phenylpropanoid derivatives by the Botrytis cinerea secretome and evaluation of their Wnt inhibition activity. Front Plant Sci. 2022;12: 805610. doi:10.3389/fpls.2021.805610

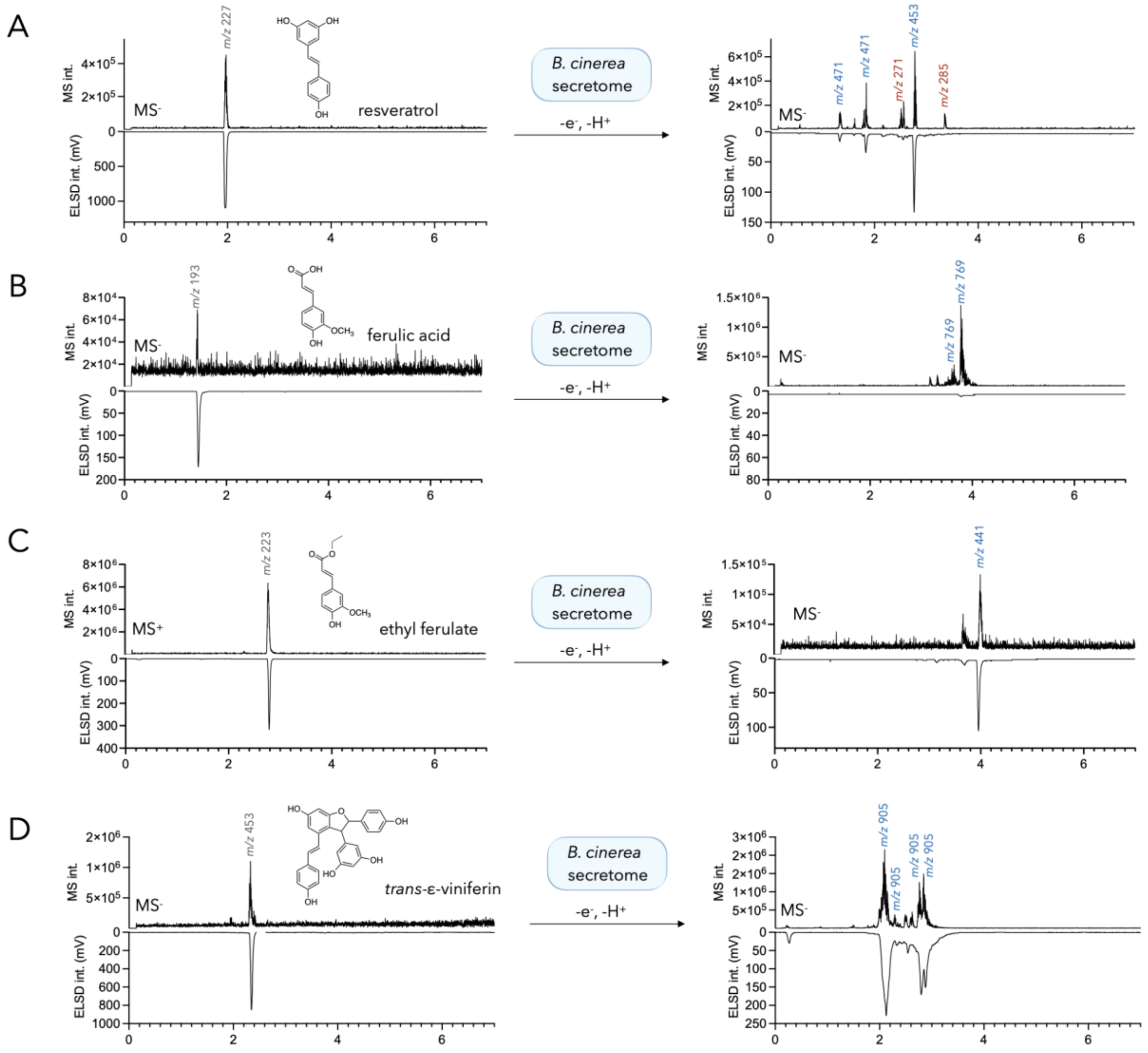

Figure S4 (first part, continued on next page) Incubation of different phenols with the enzymatic secretome of *Botrytis cinerea* (20%). Detected *m/z* corresponding to the starting materials are displayed in grey, those corresponding to dimers or higher polymers are displayed in blue, and the two signals coming from the secretome are shown in red. In the case of ferulic acid (plot B), almost no ELSD signal is visible here. In our previous work, the disappearance of the ELSD signal was shown to be time dependent, fastened with higher amount of secretome and correlated with higher *m/z* signal values in the MS detection[4]. Note that phenol (plot H) is not detected by ELSD due to its volatility.

---

[4]Huber R, Marcourt L, Koval A, Schnee S, Righi D, Michellod E, *et al*. Chemoenzymatic synthesis of complex phenylpropanoid derivatives by the Botrytis cinerea secretome and evaluation of their Wnt inhibition activity. Front Plant Sci. 2022;12: 805610. doi:10.3389/fpls.2021.805610

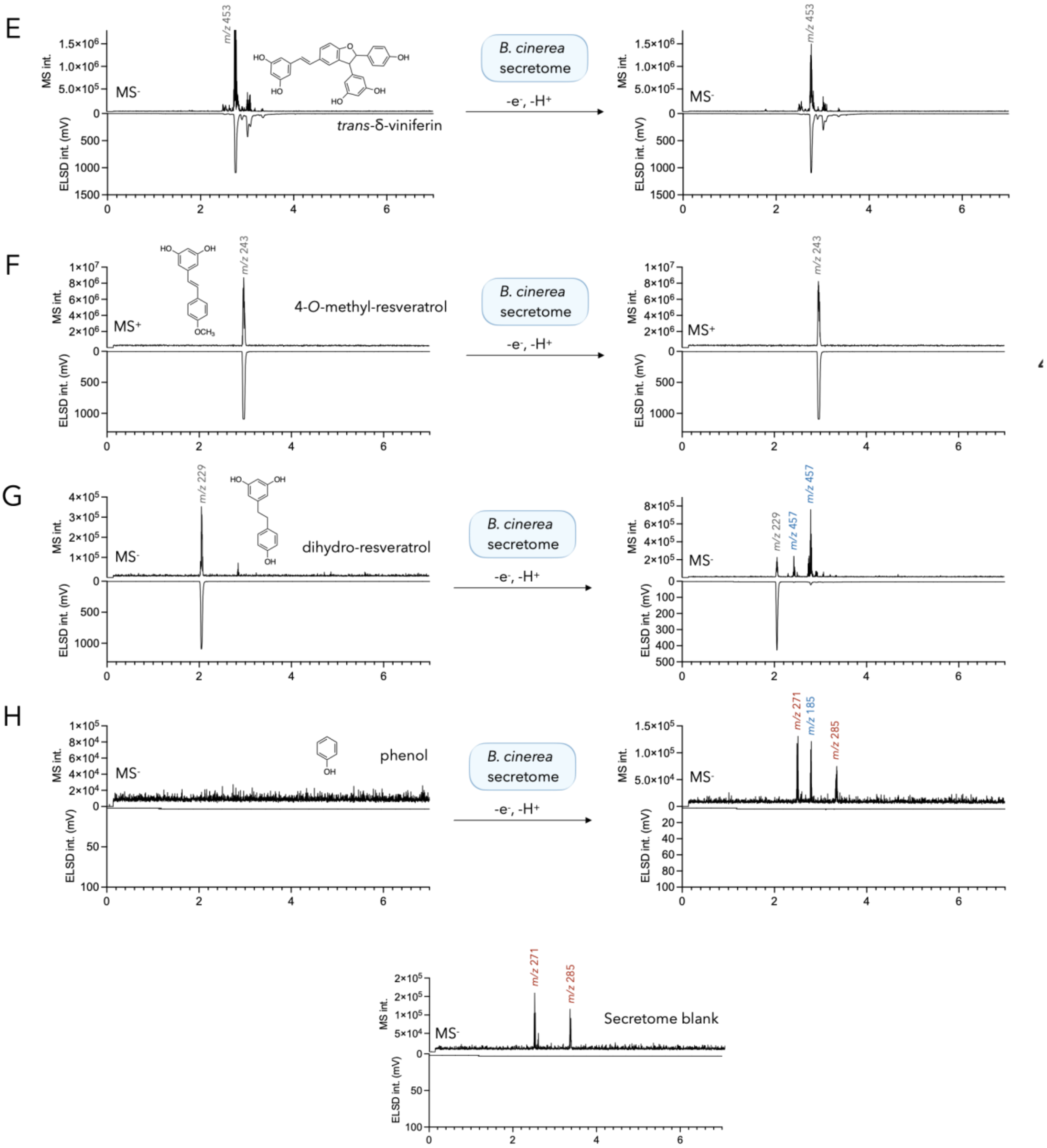

Figure S4 (second part)

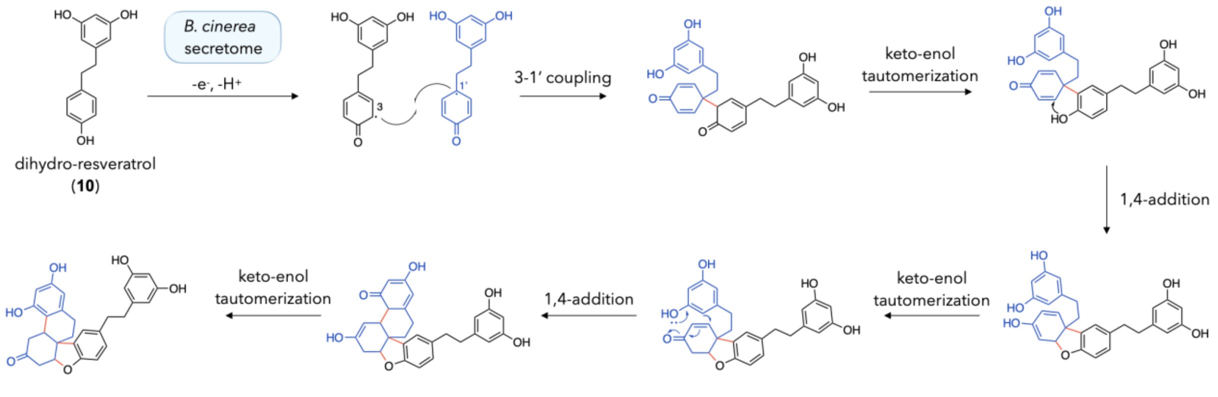

Figure S5. Proposed mechanism of formation for compounds **15** and **16**.

Table S1: ^{1}H, ^{13}C chemical shifts of compounds **15** and **16** recorded in DMSO-d_6 at 600 MHz and HMBC and ROESY correlations.
no: not observed

	**15**				**16**			
No	δ_H (Multiplicity, *J*)	δ_C	HMBC	ROESY	δ_H (Multiplicity, *J*)	δ_C	HMBC	ROESY
1	-	134.9			-	134.0	-	-
2	7.27 (d, 1.9 Hz)	123.7	C-4, CH-6, CH_2-7, C-1'	H_2-7, H_2-8, H-7'a, H-7'b	6.75 (d, 1.9 Hz)	124.0	C-4, CH-6, CH_2-7, C-1'	H_2-7, H-8a, H-2', H_2-8'
3	-	131.6	-	-	-	134.4	-	-
4	-	156.4	-	-	-	156.4	-	-
5	6.70 (d, 8.1 Hz)	109.2	C-1, C-3	-	6.71 (d, 8.1 Hz)	109.0	C-1, C-3	-
6	7.01 (dd, 8.2,1.9 Hz)	128.7	CH-2, C-4, CH_2-7	H_2-7, H_2-8	6.99 (dd, 8.1,1.9 Hz)	128.4	CH-2, C-4, CH_2-7	H_2-7, H-8a
7	2.74 (t, 8.2 Hz)	36.6	C-1, CH-2, CH-6, CH_2-8, C-9	H-2, H-6, H-10, H-14	2.63 (overlapped)	36.5	C-1, CH-2, CH-6, C-9	H-2, H-6
8	2.64 (t, 8.0 Hz)	37.9	CH_2-7, C-9, CH-10, CH-14	H-2, H-6, H-10, H-14	2.53 (overlapped)<br>2.49 (overlapped)	37.7	CH-10, CH-14, C-9	H-2, H-6
9	-	143.6	-	-	-	143.4	-	-
10	6.07 (d, 2.2 Hz)	106.4	CH_2-8, CH-12, CH-14, C-11	H_2-7, H_2-8, 11-OH	6.00 (s)	106.4	CH_2-8, C-11, CH-12, CH-14	11-OH,
11	-	158.2	-	-	-	158.2		
12	6.03 (t, 2.2 Hz)	100.2	CH-10, C-11, C-13, CH-14	11-OH, 13-OH	6.00 (s)	100.1	CH-10, C-11, C-13, CH-14	11-OH, 13-OH
13	-	158.2	-	-	-	158.2	-	-
14	6.07 (d, 2.2 Hz)	106.4	CH_2-8, CH-10, CH-12, C-13	H_2-7, H_2-8, 13-OH	6.00 (s)	106.4	CH_2-8, CH-10, CH-12, C-13	13-OH,
1'	-	48.4	-	-	-	46.1	-	-
2'	6.67 (dd, 10.2,1.9 Hz)	149.8	C-1', C-4', C-6'	H-3'	3.23 (dd, 9.1,3.8 Hz)	37.0	CH_2-3', C-4', C-14'	H-2
3'	5.93 (d, 10.2 Hz)	126.3	C-1', CH_2-5'	H-2'	2.69 (dd, 17.0,9.1 Hz)<br>2.32 (dd, 17.0,3.8 Hz)	41.4	C-1', C-4'<br>C-1', C-4'	H-6'

4'	-	195.5	-	-	-	208.8	-	-
5'	3.02 (dd, 17.6,4.0 Hz)<br>2.79 (dd, 17.7,2.7 Hz)	38.9	C-4'<br>C-1', C-4', CH-6'	H-6'<br>H-6'	2.72 (dd, 16.7,7.1 Hz)<br>2.45 (dd, 16.7,4.4 Hz)	42.3	C-4', CH-6'<br>C-4'	H-6'
6'	4.99 (dt, 4.2,2.3 Hz)	84.3	CH-2', C-4'	H-5'a, H-5'b, H-7'a, H-7'b, H_2-8'	4.80 (dd, 7.1,4.4 Hz)	86.7	CH_2-7', C-4'	H-3'a, H-5'b, H-7'a, H-7'b
7'	2.27 (td, 13.2,12.7,4.9 Hz)<br>2.10 (td, 13.7,12.3,5.6 Hz)	36.5	C-1', CH-6', C-3<br>C-1', CH-2', CH-6'	H-6', H-10', H-14', H-2<br>H-6', H-10', H-14', H-2	2.07 (dt, 12.7,6.4 Hz)<br>1.90 (dt, 12.7,6.4 Hz)	30.1	C-1', CH-2', CH-6', CH_2-8', C-9', C-3,<br>C-1', CH-2', CH-6', CH_2-8', C-9'	H-6'<br>H-6'
8'	2.44 (m)	30.4	C-1', C-9', CH-10', CH-14'	H-6', H-10', H-14'	2.72 (overlapped)	25.4	C-9'	H-2, H-10'
9'	-	143.5	-		-	137.8	-	-
10'	6.10 (d, 2.2 Hz)	106.4	CH_2-8', CH-12', C-11', CH-14'	H7'a, H-7'b, H_2-8', 11'-OH	6.08 (d, 2.4 Hz)	106.3	CH_2-8', C-11', CH-12', C-14'	H_2-8', 11'-OH
11'	-	158.3			-	156.2		
12'	6.04 (t, 2.2 Hz)	100.3	CH-10', C-11', C-13', CH-14'	11'-OH, 13'-OH	6.16 (d, 2.4 Hz)	101.0	CH-10', C-14', C-11', C-13'	11'-OH, 13'-OH
13'	-	no	-	-	-	156.2	-	-
14'	6.10 (d, 2.2 Hz)	106.4	CH_2-8', CH-10', CH-12', C-13'	H7'a, H-7'b, H_2-8', 13'-OH	-	114.2	-	-
11-OH	9.04 (s)	-	CH-10, C-11, CH-12	H-10, H-12	9.00 (s)	-	CH-10, C-11, CH-12	H-10, H-12
13-OH	9.04 (s)	-	CH-12, C-13, CH-14	H-12, H-14	9.00 (s)	-	CH-12, C-13, CH-14	H-12, H-14
11'-OH	9.07 (s)	-	CH-10', C-11', CH-12'	H-10', H-12'	9.04 (s)	-	CH-10', C-11', CH-12'	H-10', H-12'
13'-OH	9.07 (s)	-	CH-12', C-13', CH-14'	H-12', H-14'	9.33 (s)	-	CH-12', C-13', CH-14'	H-12'

Table S2. Detailed volumes for the biotransformation reactions of phenols

substrate	Vol. stock substrate (µL)	Vol. H_2O (µL)	Vol. secretome (µL)
resveratrol	100	850	50 µL (5%)
resveratrol	100	700	200 µL (20%)
resveratrol	100	900	- (neg control)
trans-δ-viniferin	100	850	50 µL (5%)
trans-δ-viniferin	100	700	200 µL (20%)
trans-δ-viniferin	100	900	- (neg control)
4-*O*-methyl-resveratrol	100	850	50 µL (5%)
4-*O*-methyl-resveratrol	100	700	200 µL (20%)
4-*O*-methyl-resveratrol	100	900	- (neg control)
dehydro-resveratrol	100	850	50 µL (5%)
dehydro-resveratrol	100	700	200 µL (20%)
dehydro-resveratrol	100	900	- (neg control)
phenol	100	850	50 µL (5%)
phenol	100	700	200 µL (20%)
phenol	100	900	- (neg control)
-	100 (acetone)	850	50 µL (5%)
-	100 (acetone)	700	200 µL (20%)

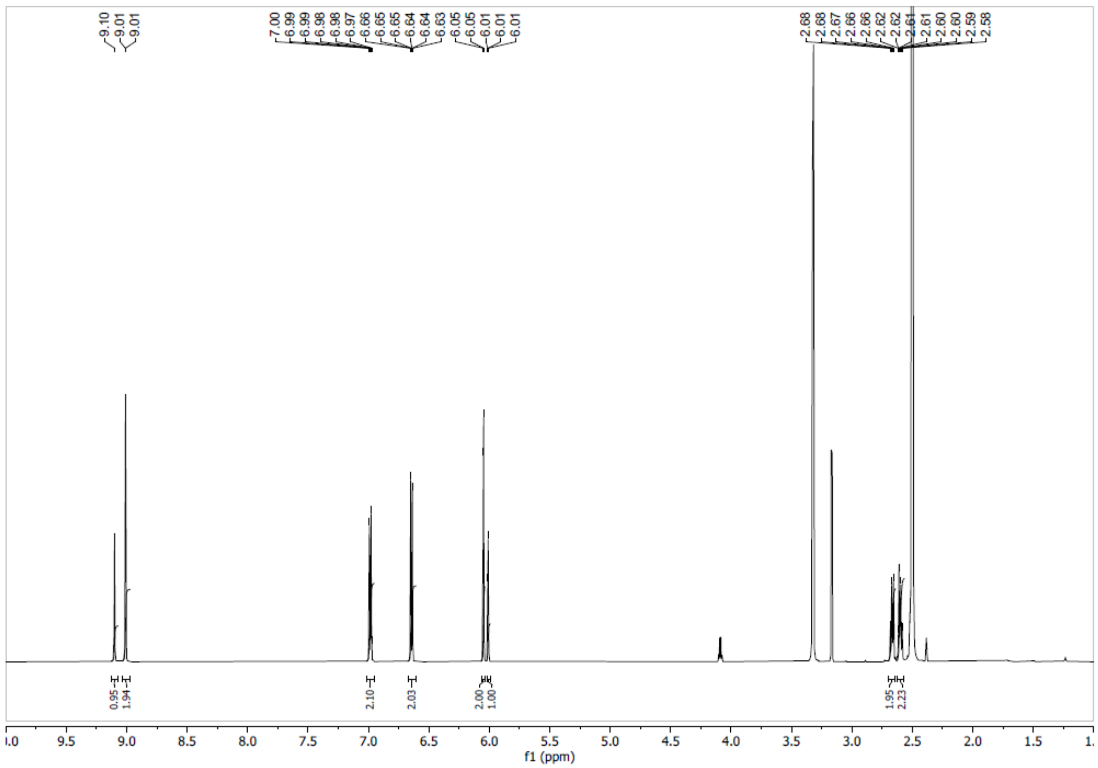

Figure S6. ^{1}H NMR spectrum of compound **10** in DMSO-d_6 at 600 MHz

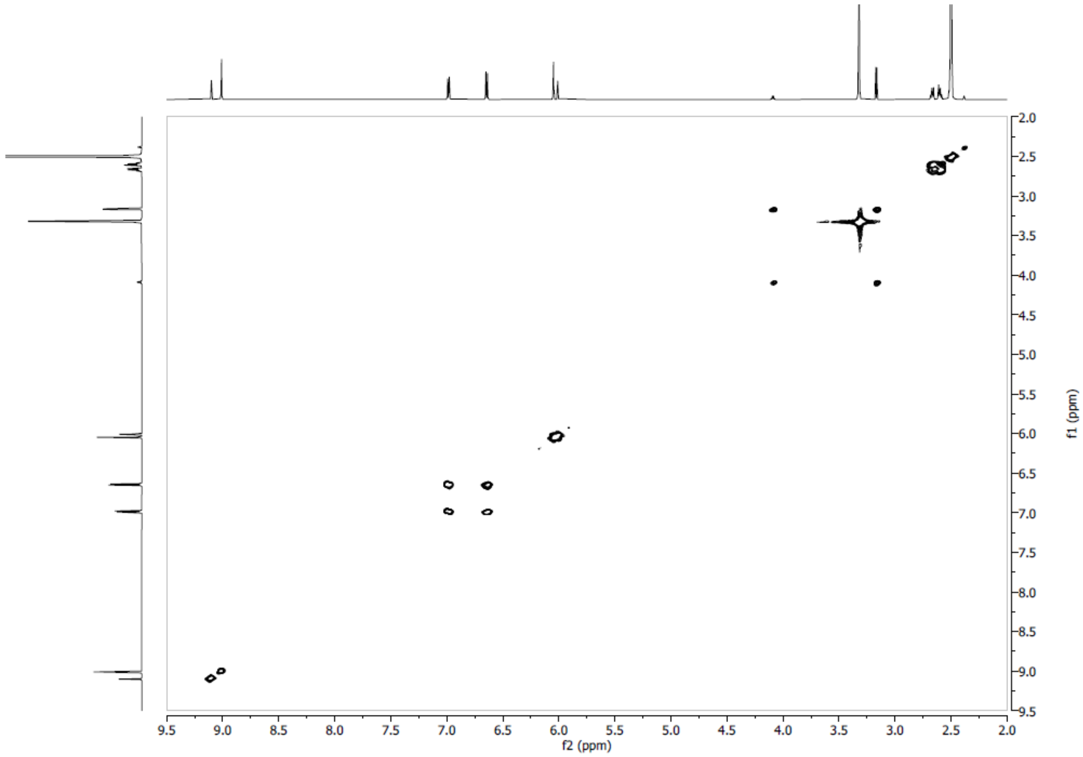

Figure S7. COSY NMR spectrum of compound **10** in DMSO-d_6

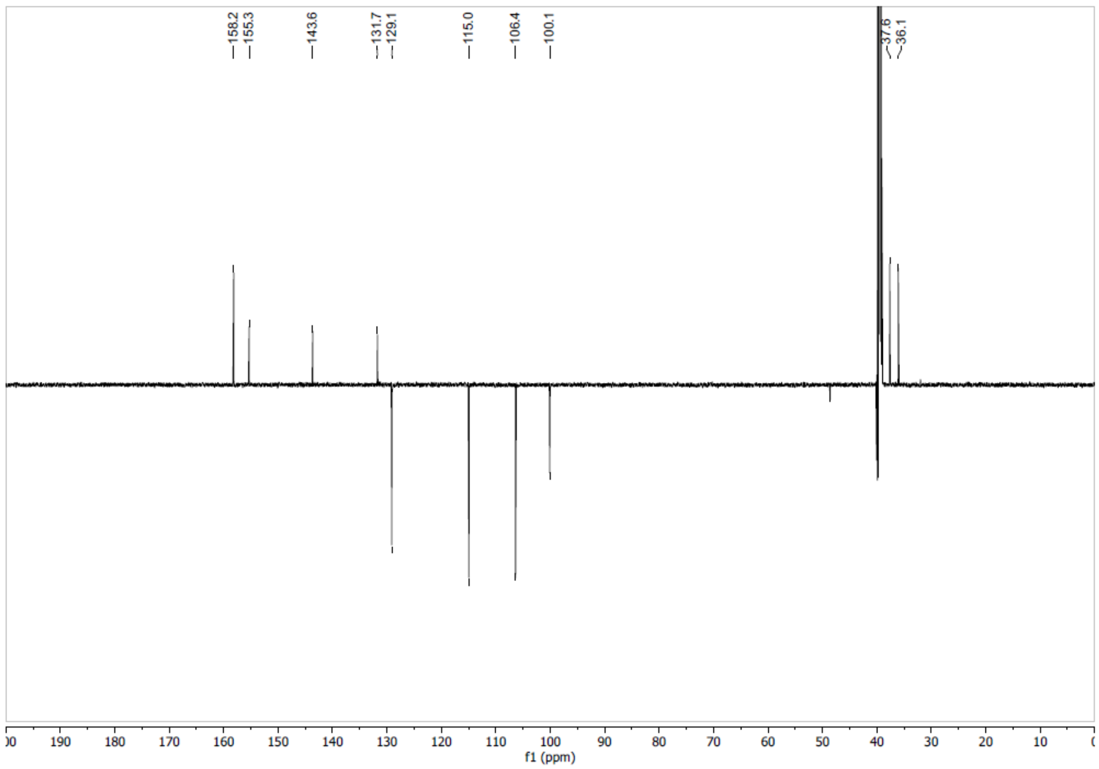

Figure S8. ^{13}C-DEPTQ NMR spectrum of compound **10** in DMSO-d_6 at 151 MHz

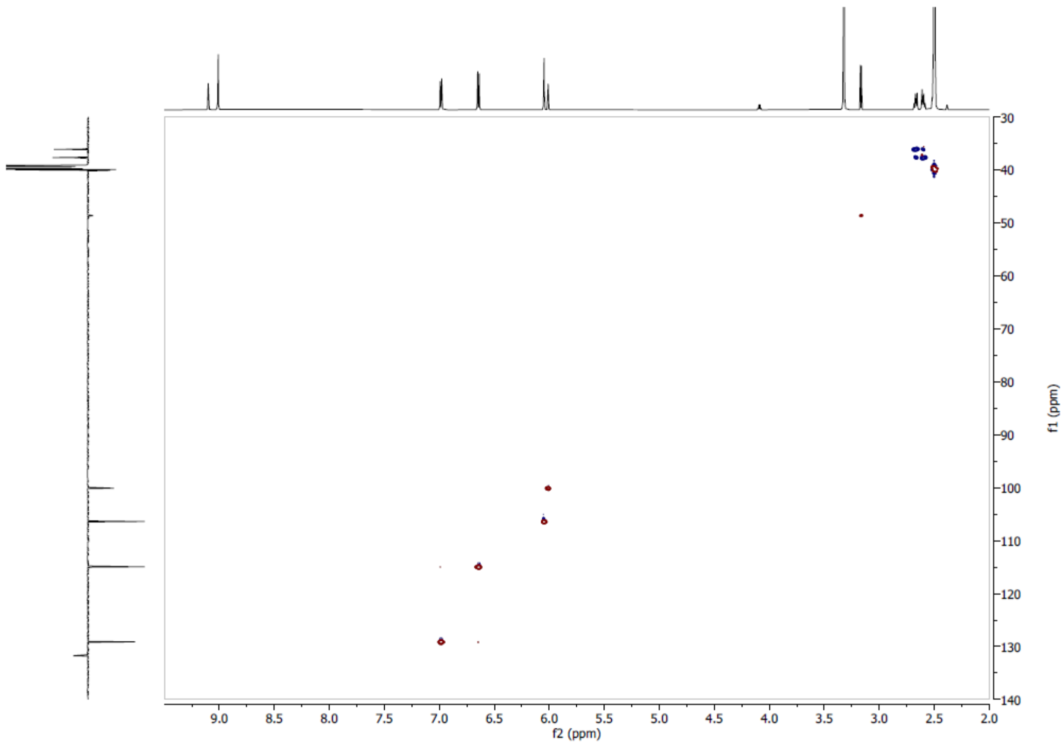

Figure S9. Edited HSQC NMR spectrum of compound **10** in DMSO-d_6

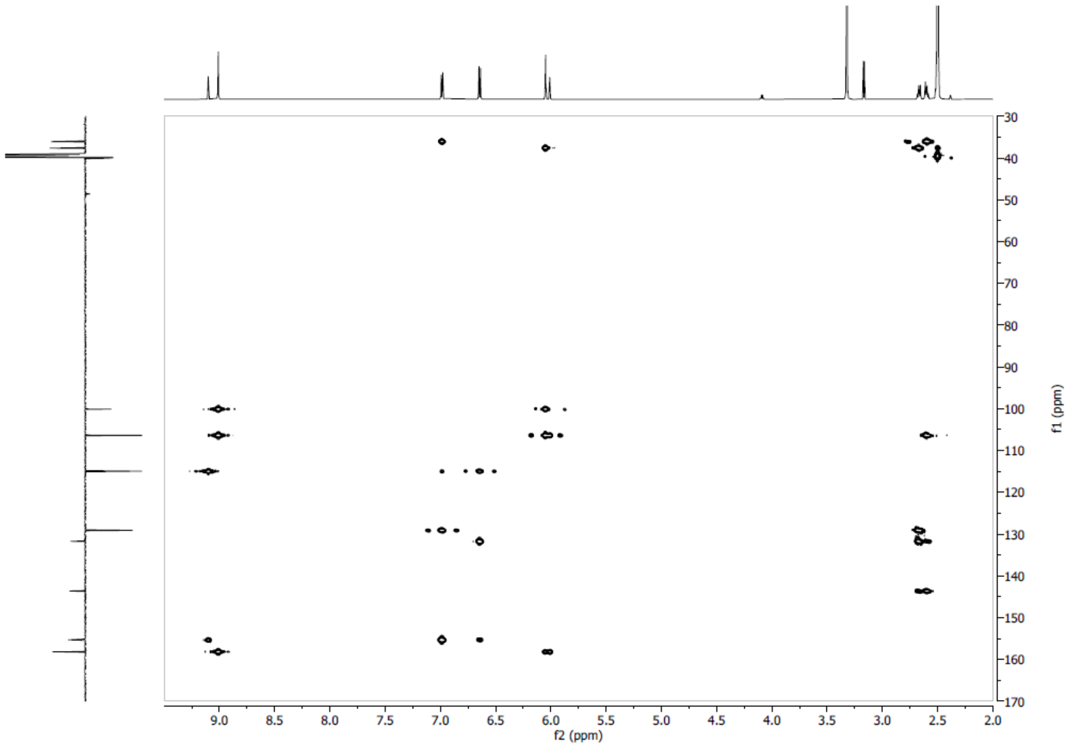

Figure S10. HMBC NMR spectrum of compound **10** in DMSO-d_6

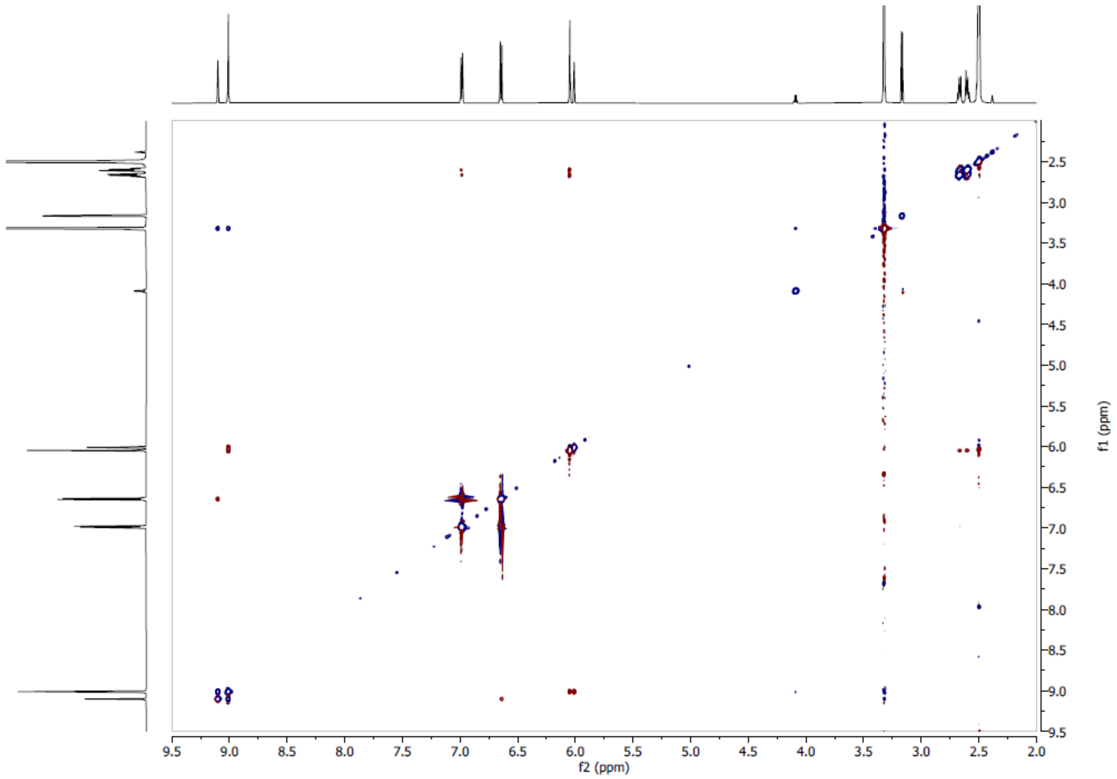

Figure S11. ROESY NMR spectrum of compound **10** in DMSO-d_6

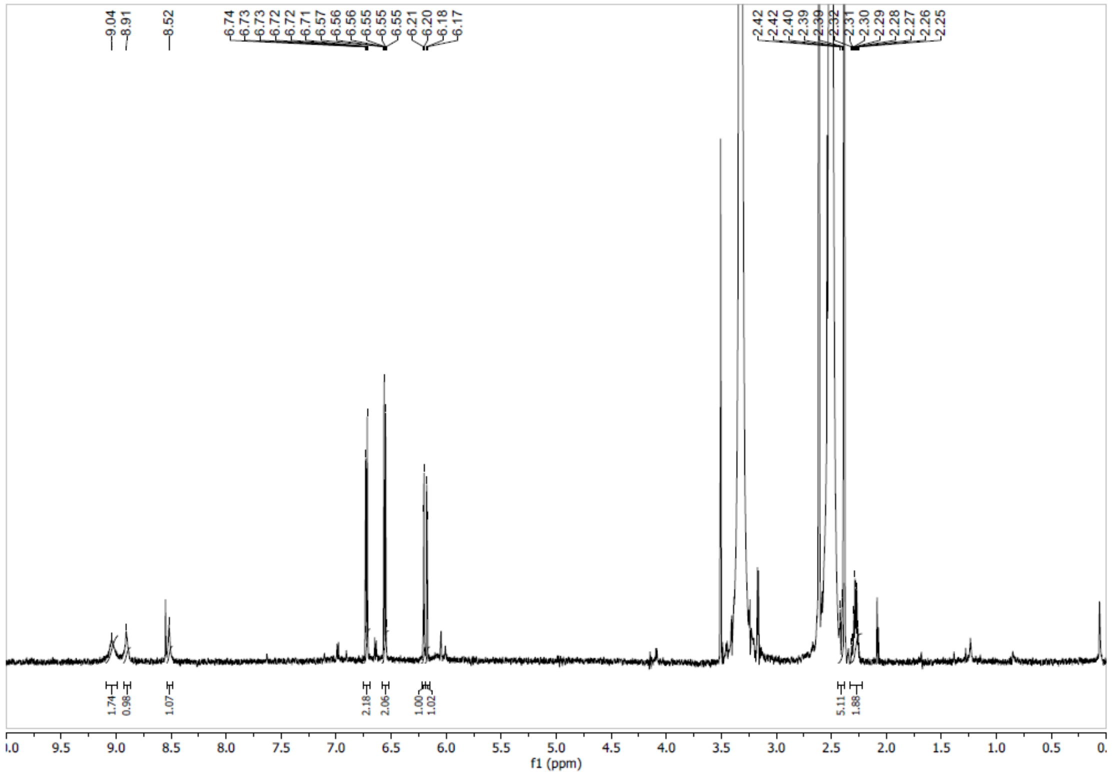

Figure S12. ^{1}H NMR spectrum of compound **11** in DMSO-d_6 at 600 MHz

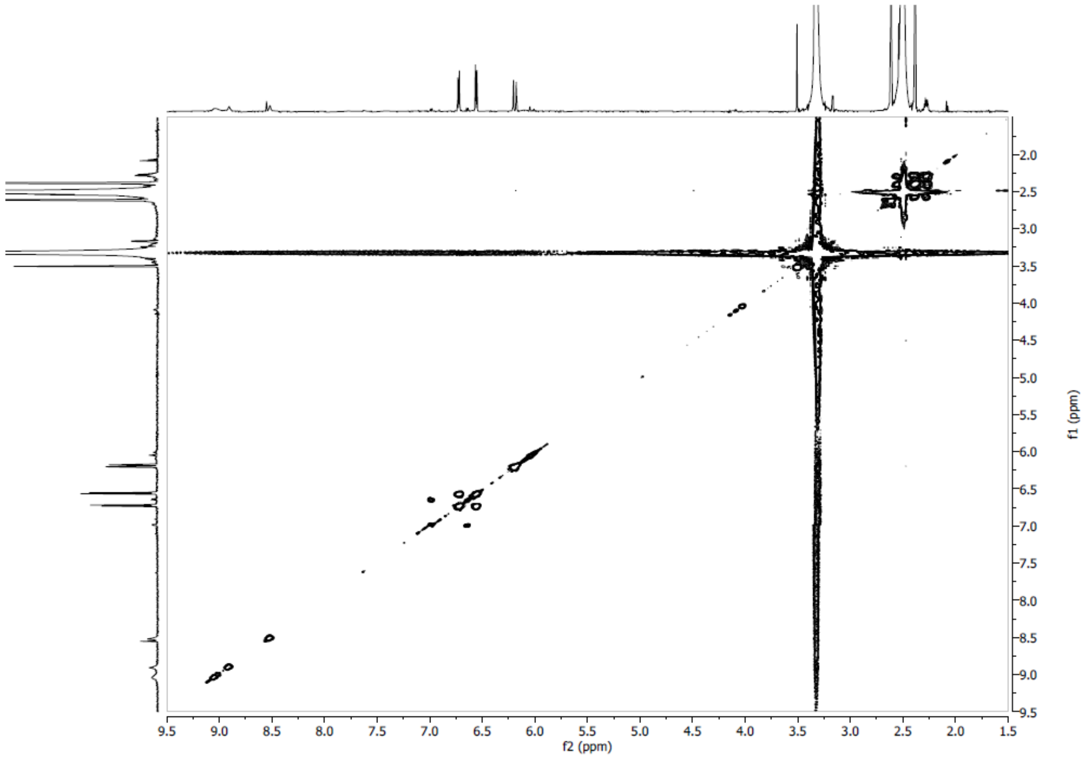

Figure S13. COSY NMR spectrum of compound **11** in DMSO-d_6

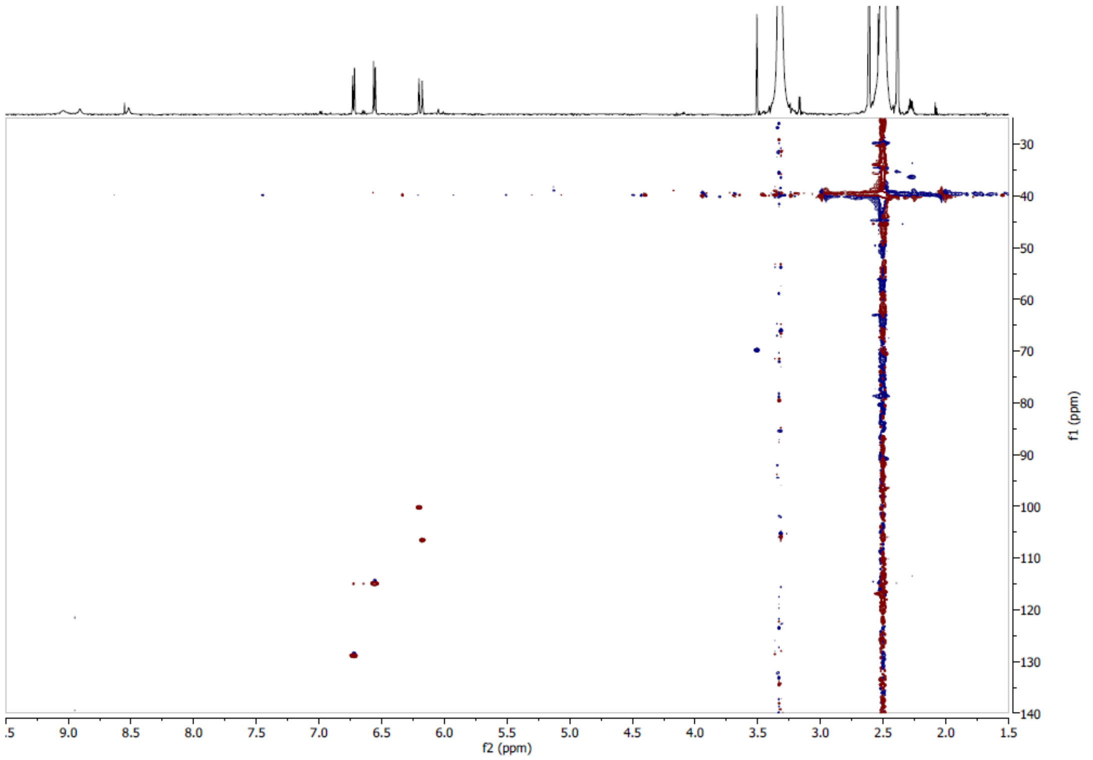

Figure S14. Edited HSQC NMR spectrum of compound **11** in DMSO-d_6

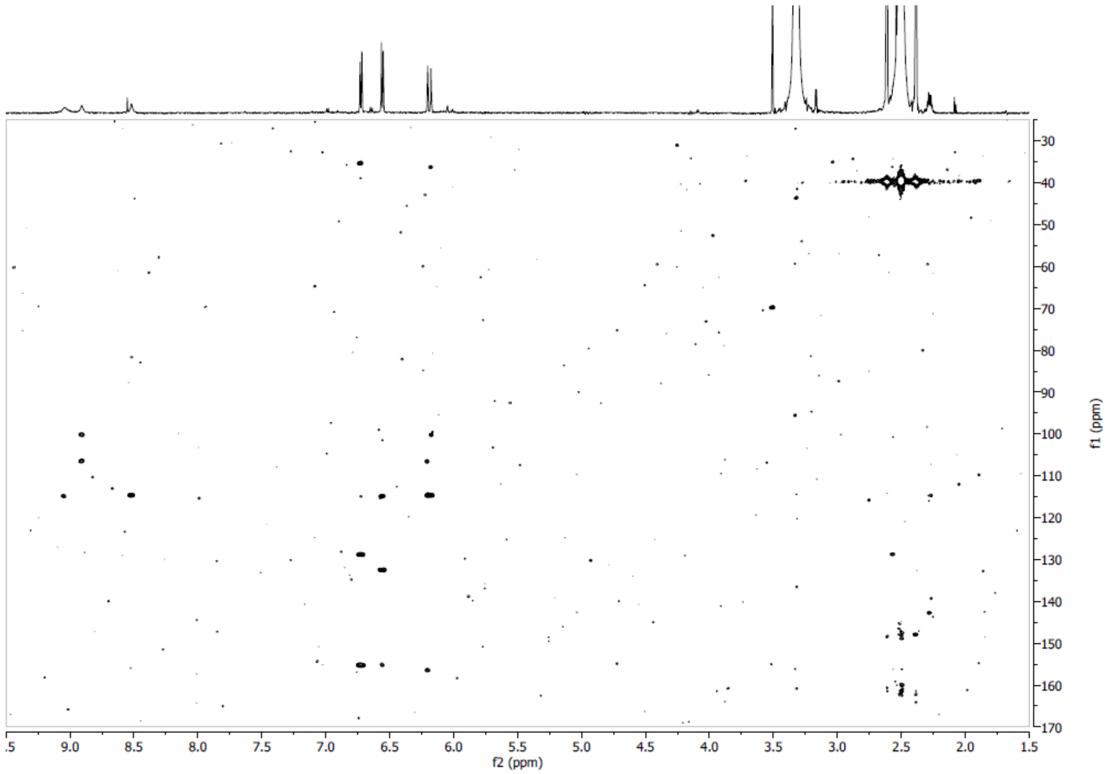

Figure S15. HMBC NMR spectrum of compound **11** in DMSO-d_6

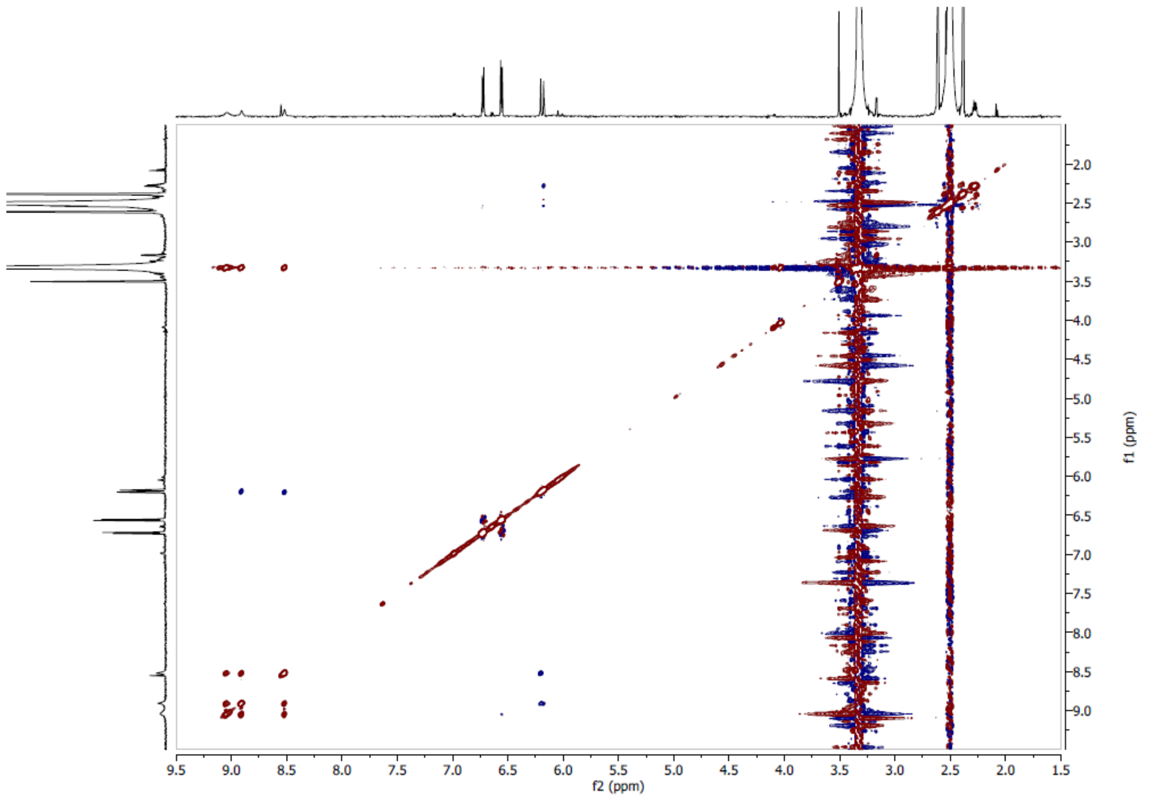

Figure S16. ROESY NMR spectrum of compound **11** in DMSO-d_6

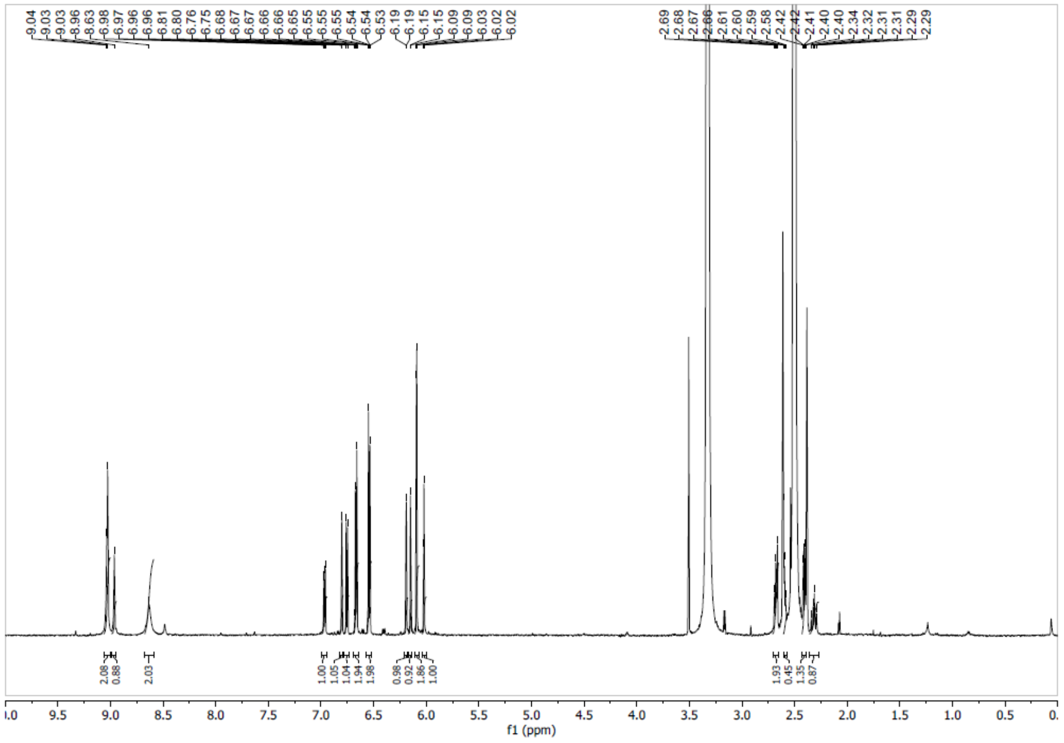

Figure S17. ^{1}H NMR spectrum of compound **12** in DMSO-d_6 at 600 MHz

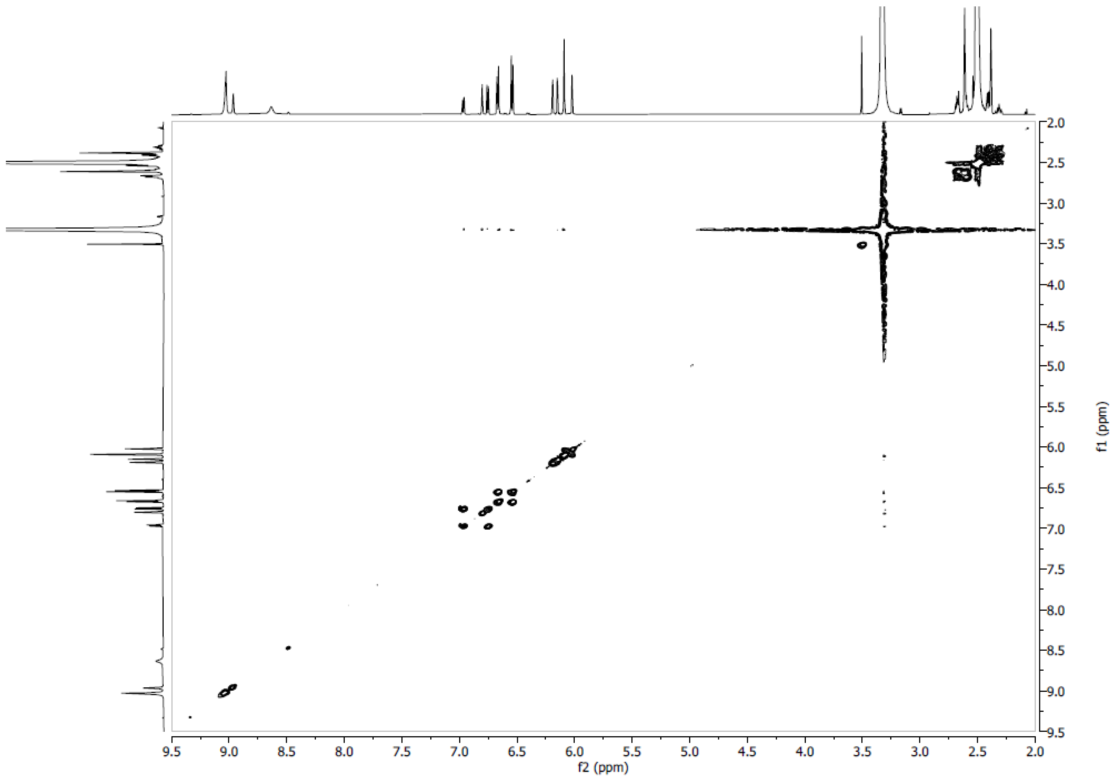

Figure S18. COSY NMR spectrum of compound **12** in DMSO-d_6

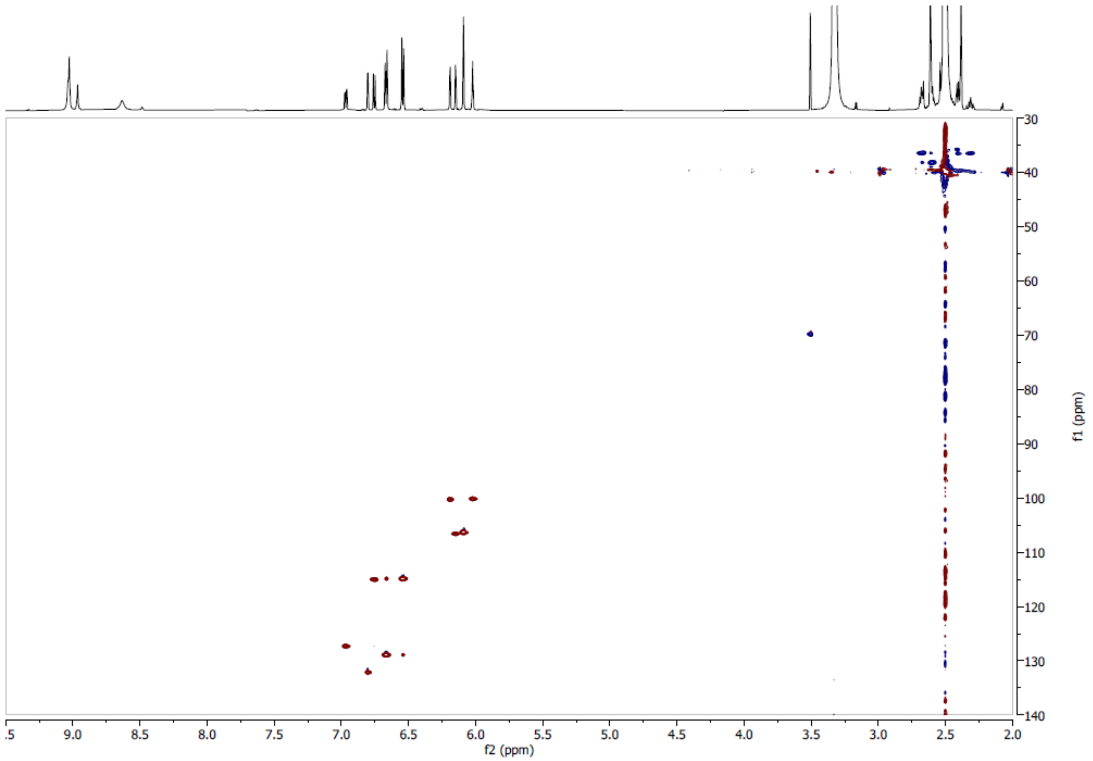

Figure S19. Edited HSQC NMR spectrum of compound **12** in DMSO-d_6

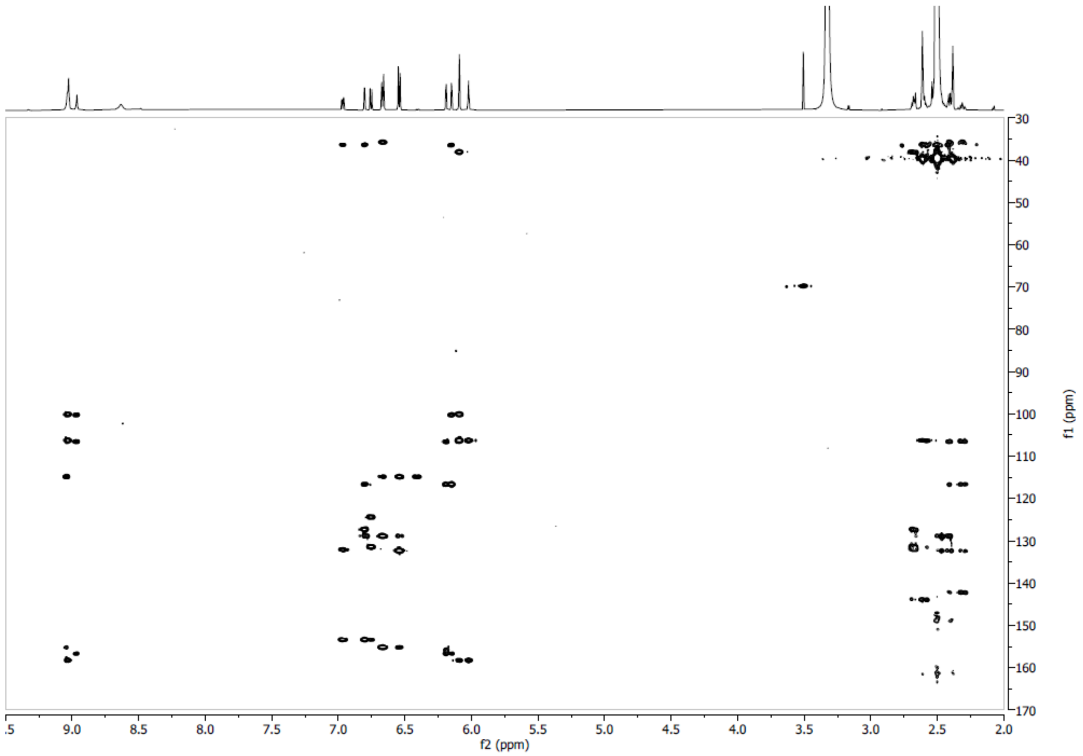

Figure S20. HMBC NMR spectrum of compound **12** in DMSO-d_6

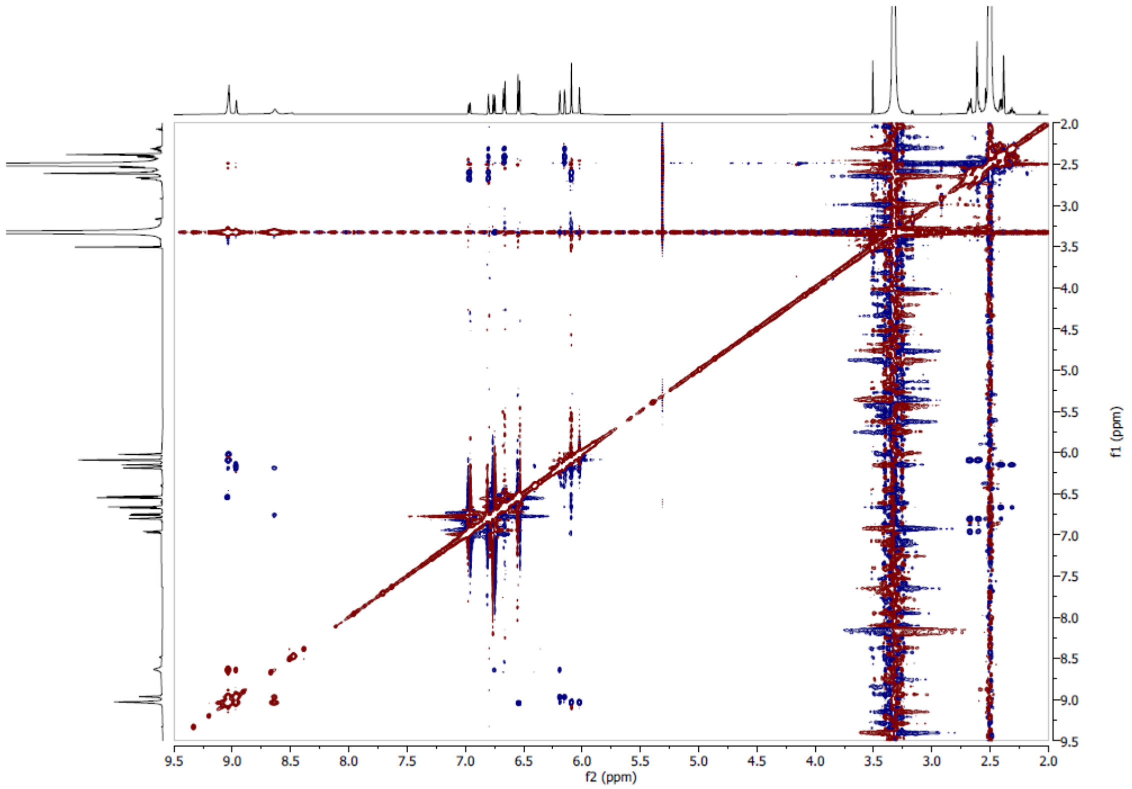

Figure S21. ROESY NMR spectrum of compound **12** in DMSO-d_6

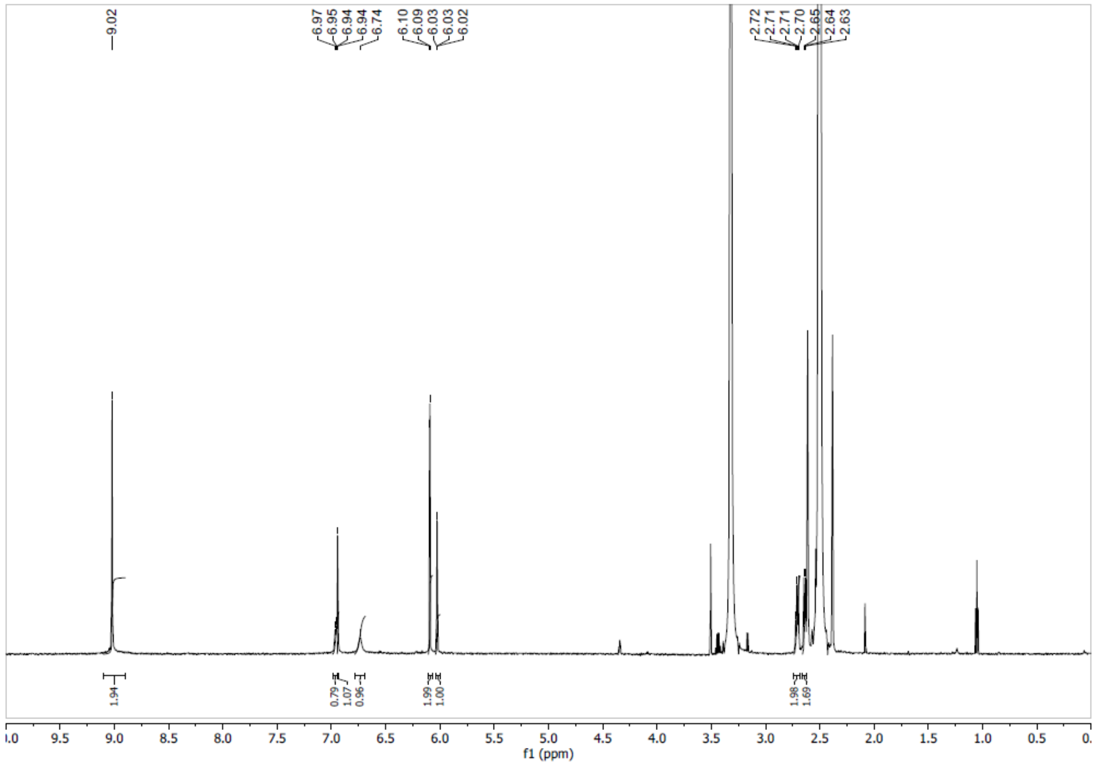

Figure S22. ^{1}H NMR spectrum of compound **13** in DMSO-d_6 at 600 MHz

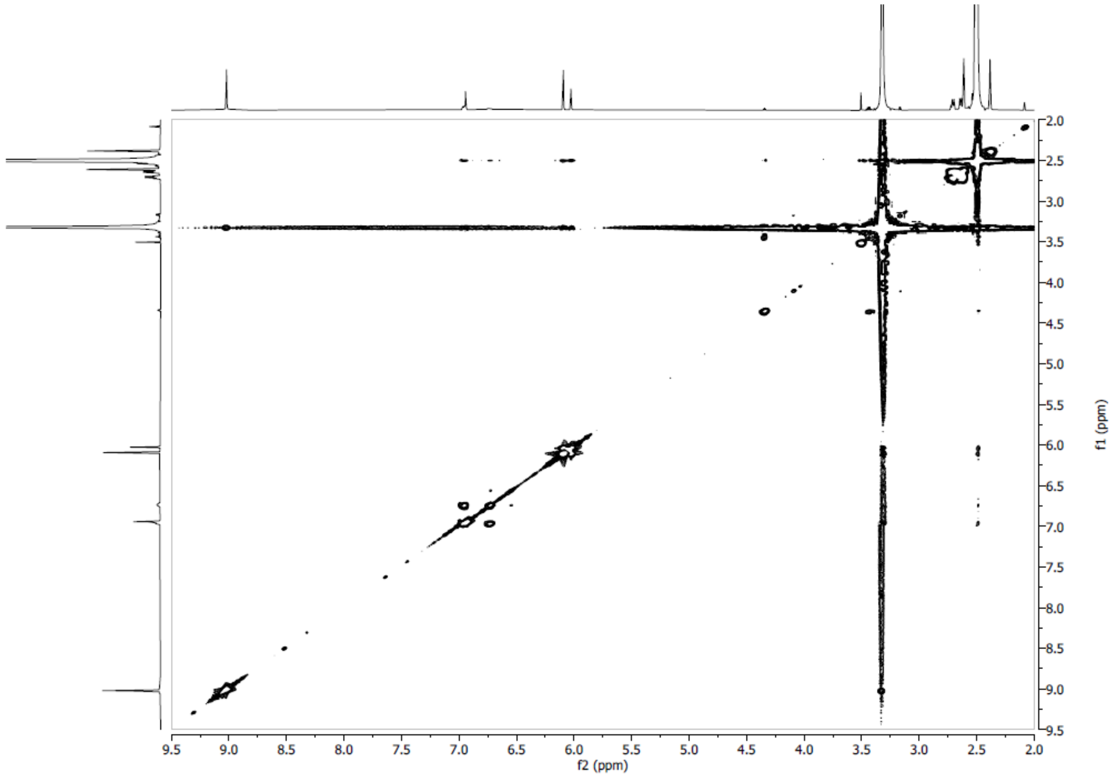

Figure S23. COSY NMR spectrum of compound **13** in DMSO-d_6

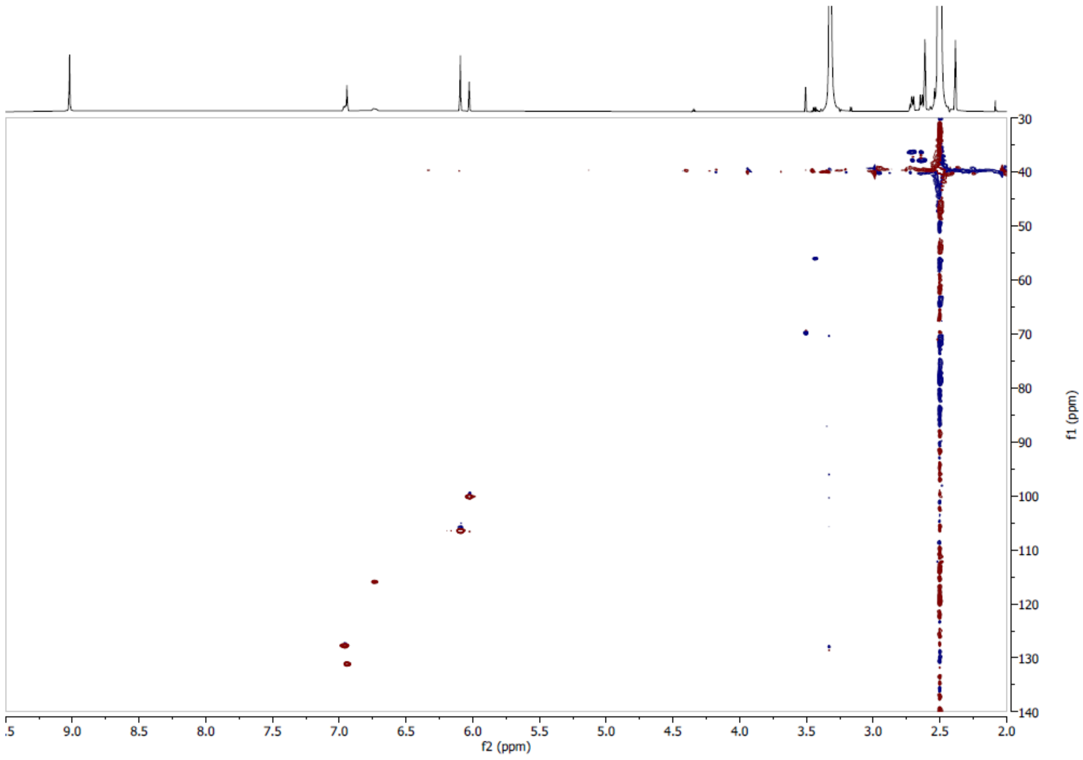

Figure S24. Edited HSQC NMR spectrum of compound **13** in DMSO-d_6

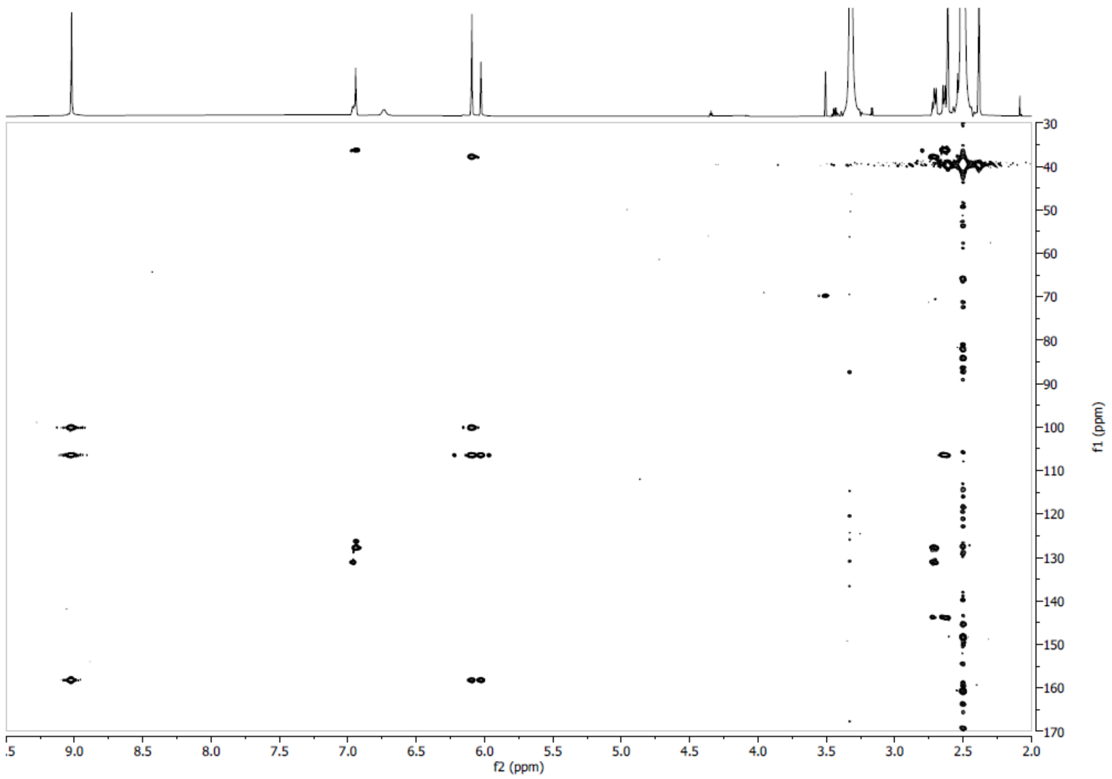

Figure S25. HMBC NMR spectrum of compound **13** in DMSO-d_6

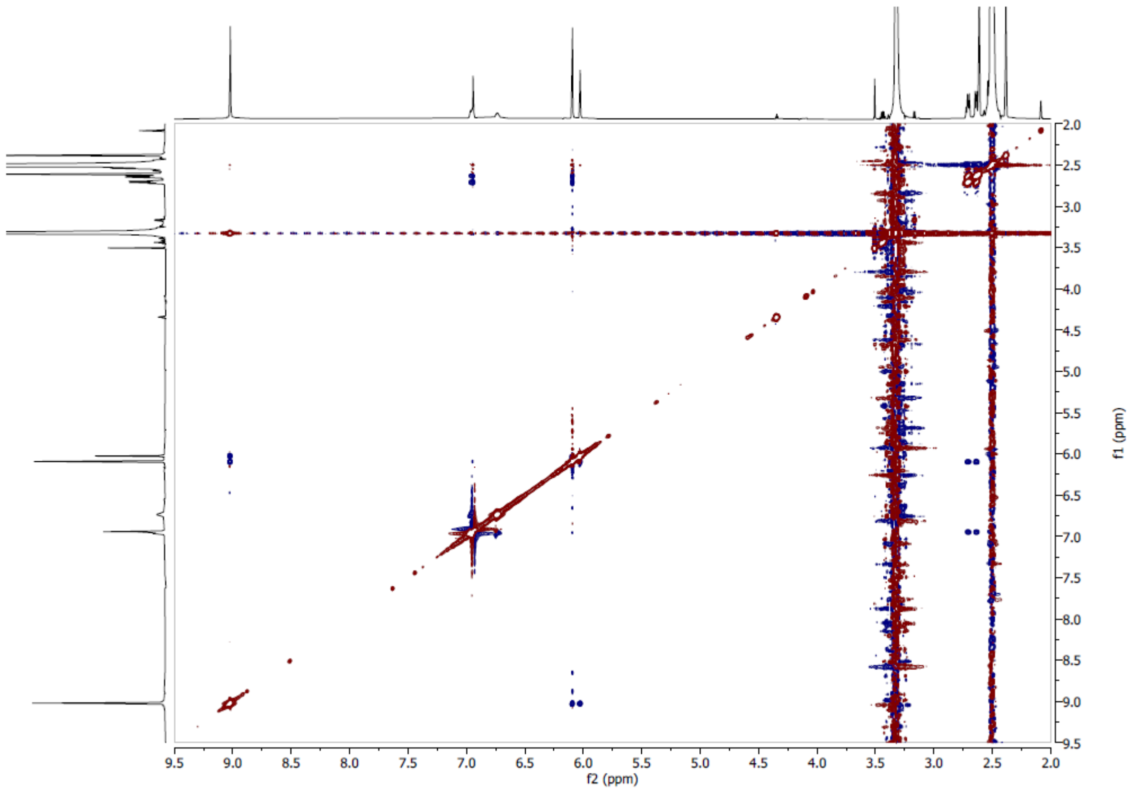

Figure S26. ROESY NMR spectrum of compound **13** in DMSO-d_6

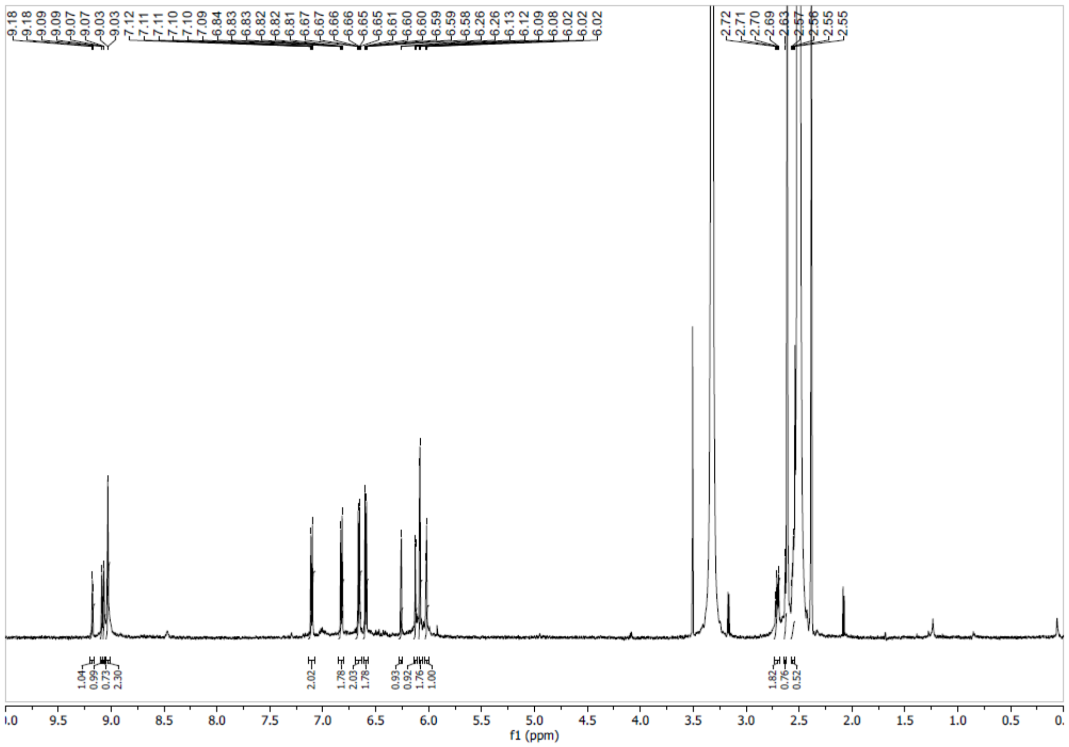

Figure S27. ^{1}H NMR spectrum of compound **14** in DMSO-d_6 at 600 MHz

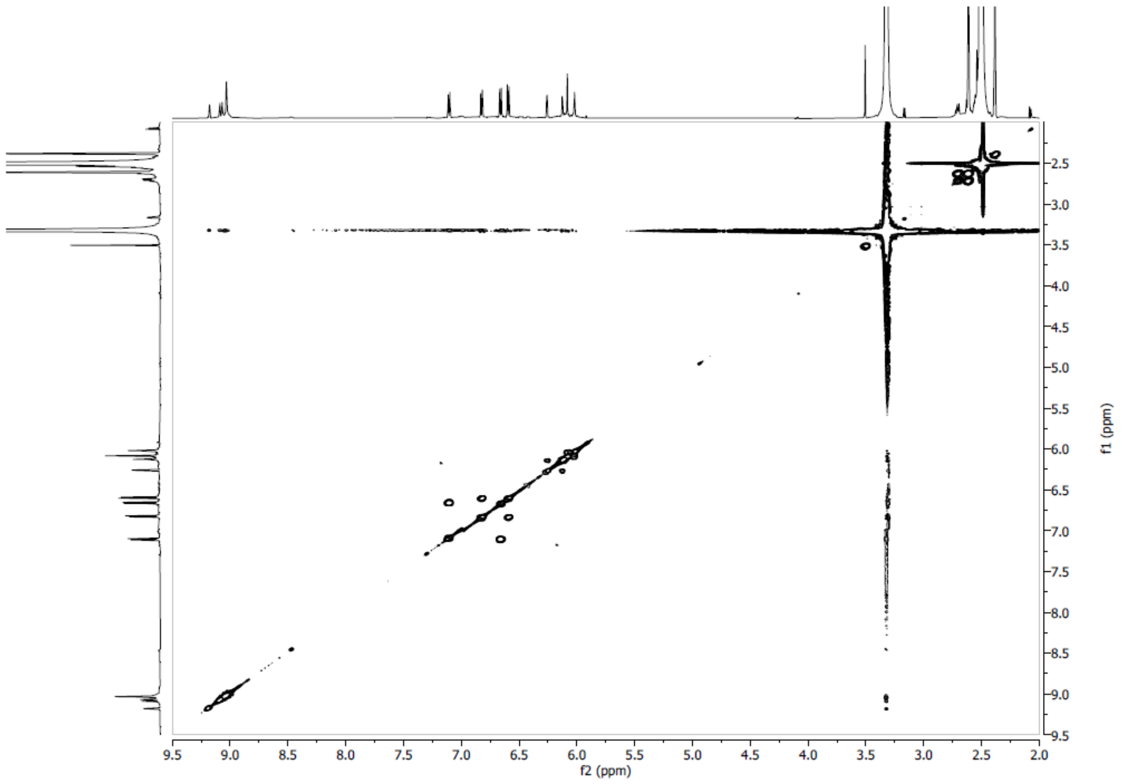

Figure S28. COSY NMR spectrum of compound **14** in DMSO-d_6

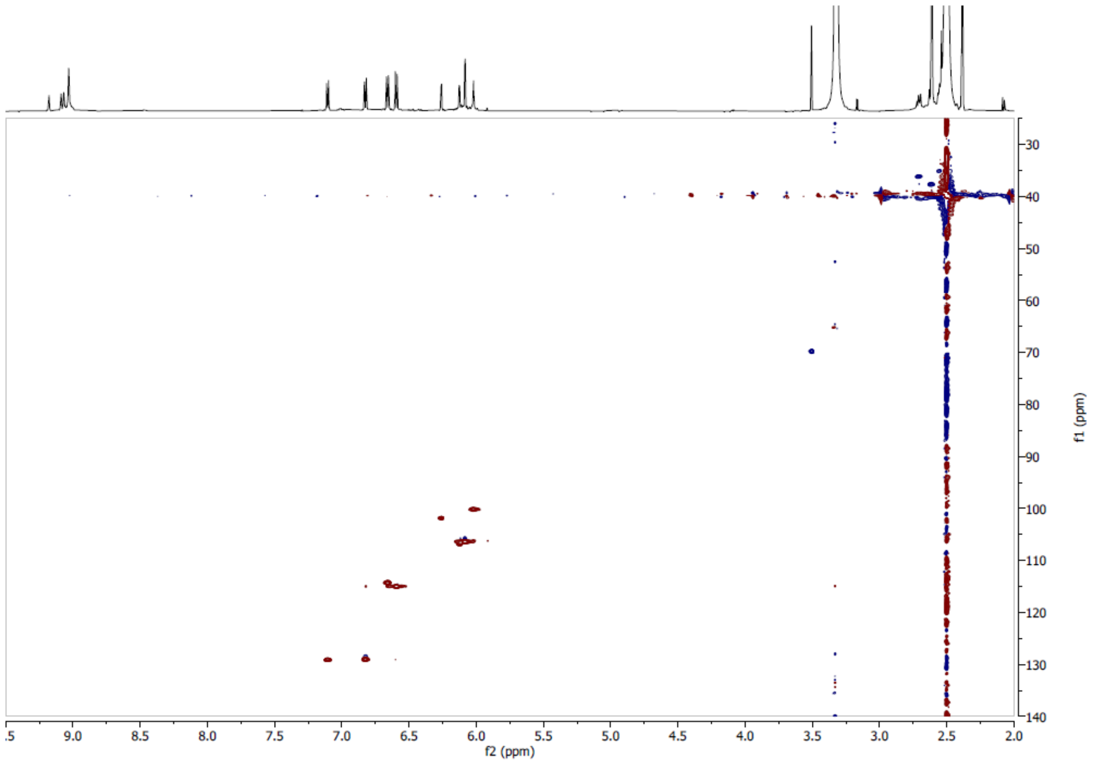

Figure S29. Edited HSQC NMR spectrum of compound **14** in DMSO-d_6

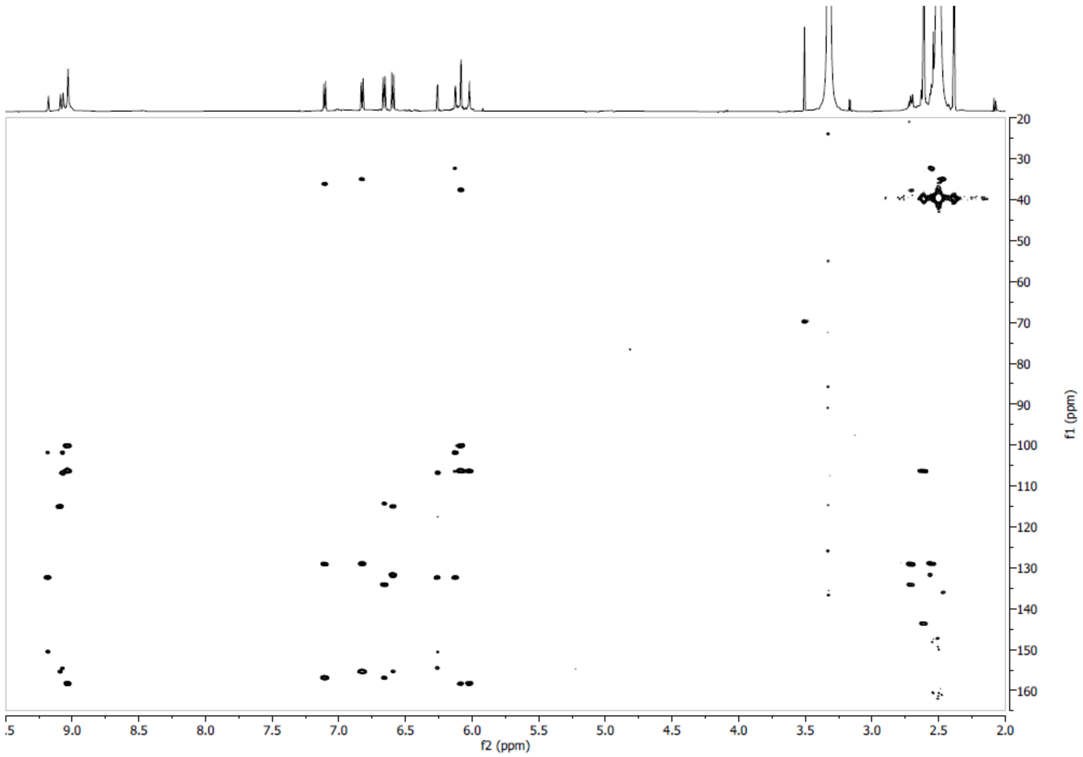

Figure S30. HMBC NMR spectrum of compound **14** in DMSO-d_6

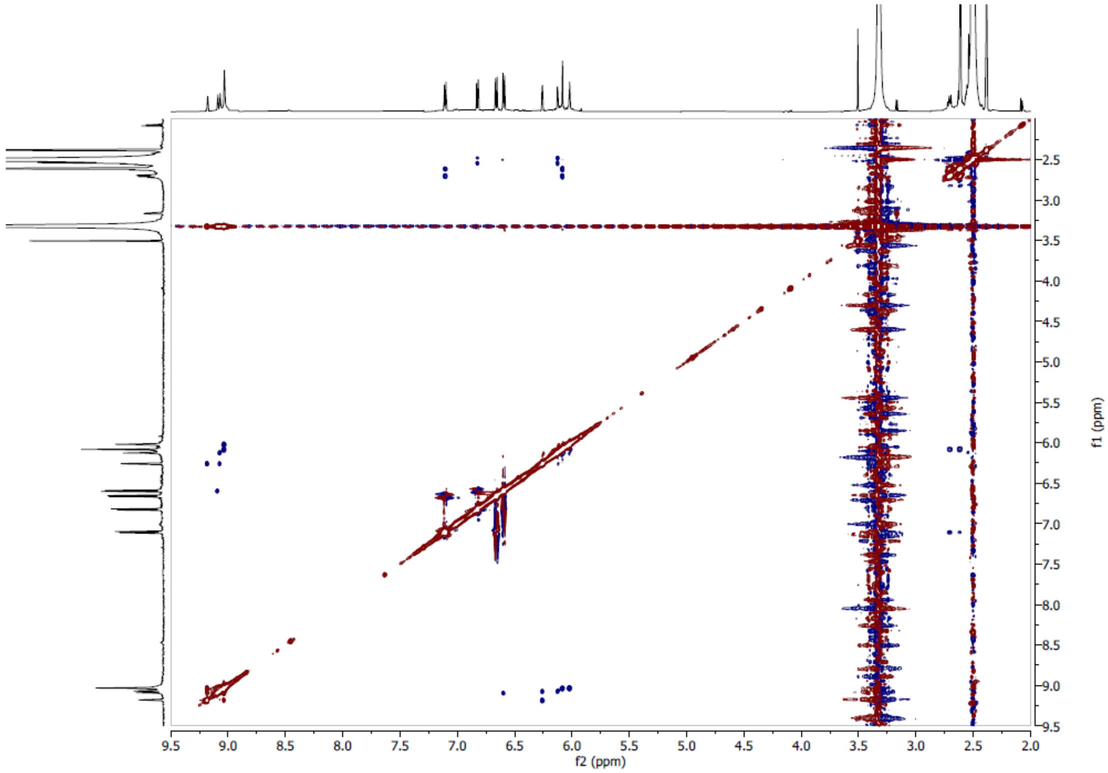

Figure S31. ROESY NMR spectrum of compound **14** in DMSO-d_6

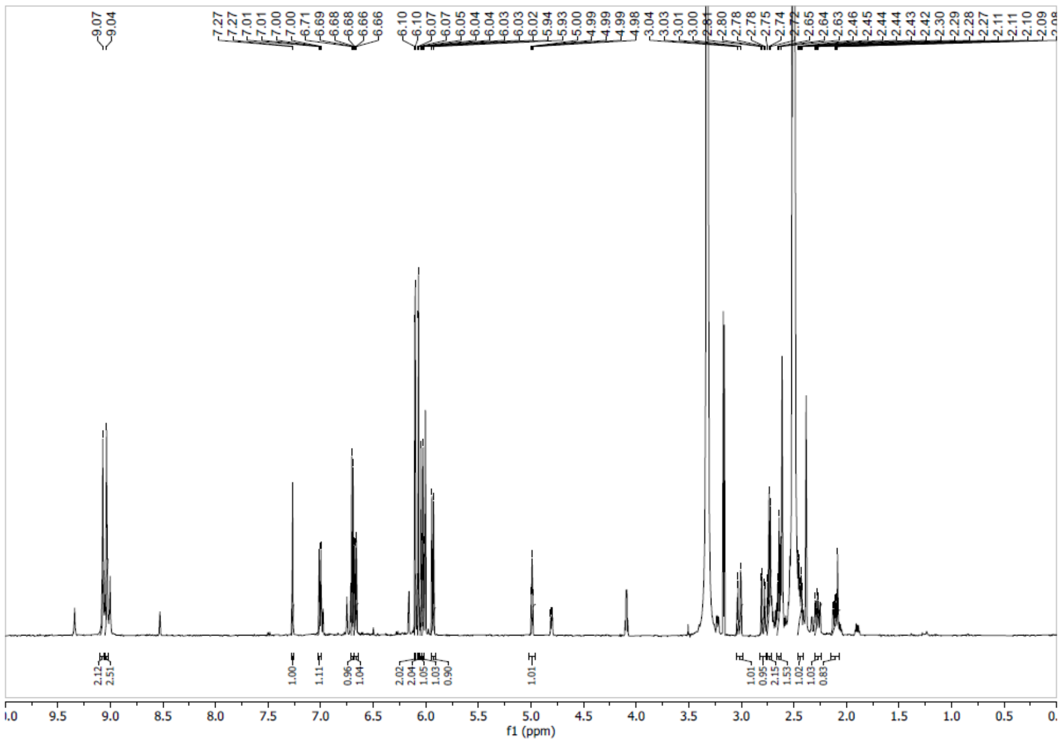

Figure S32. ^{1}H NMR spectrum of compound **15** in DMSO-d_6 at 600 MHz

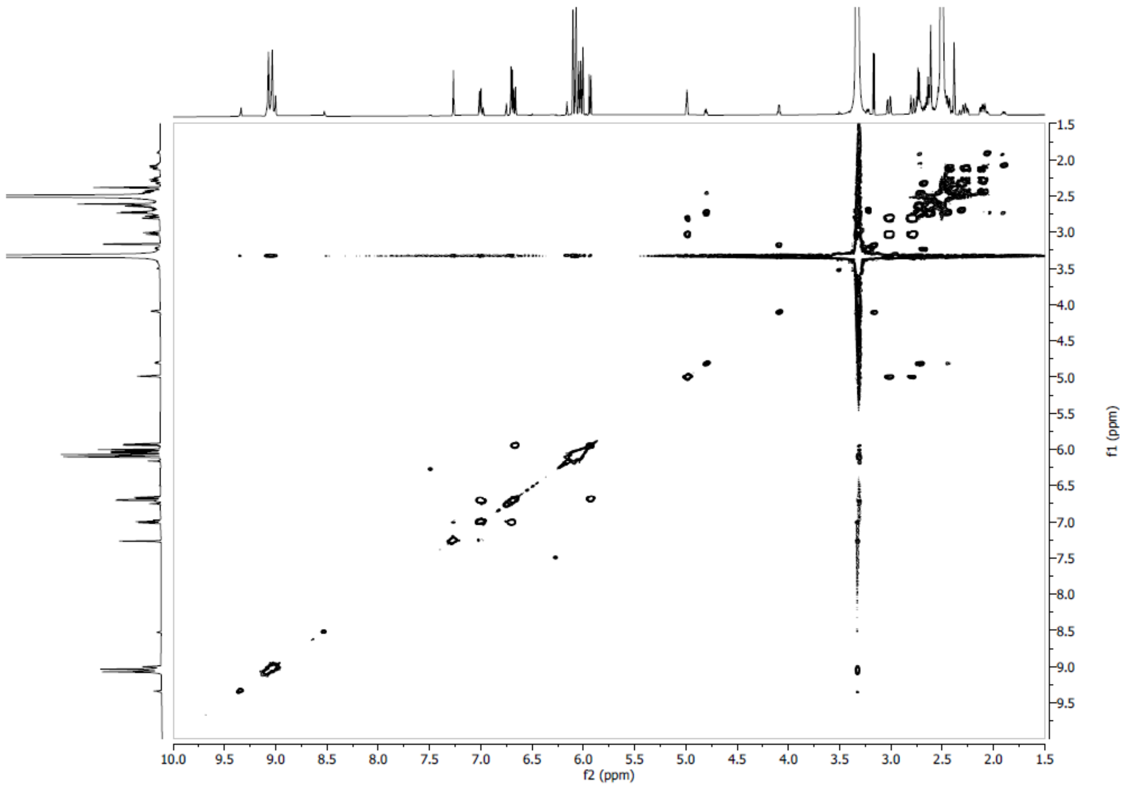

Figure S33. COSY NMR spectrum of compound **15** in DMSO-d_6

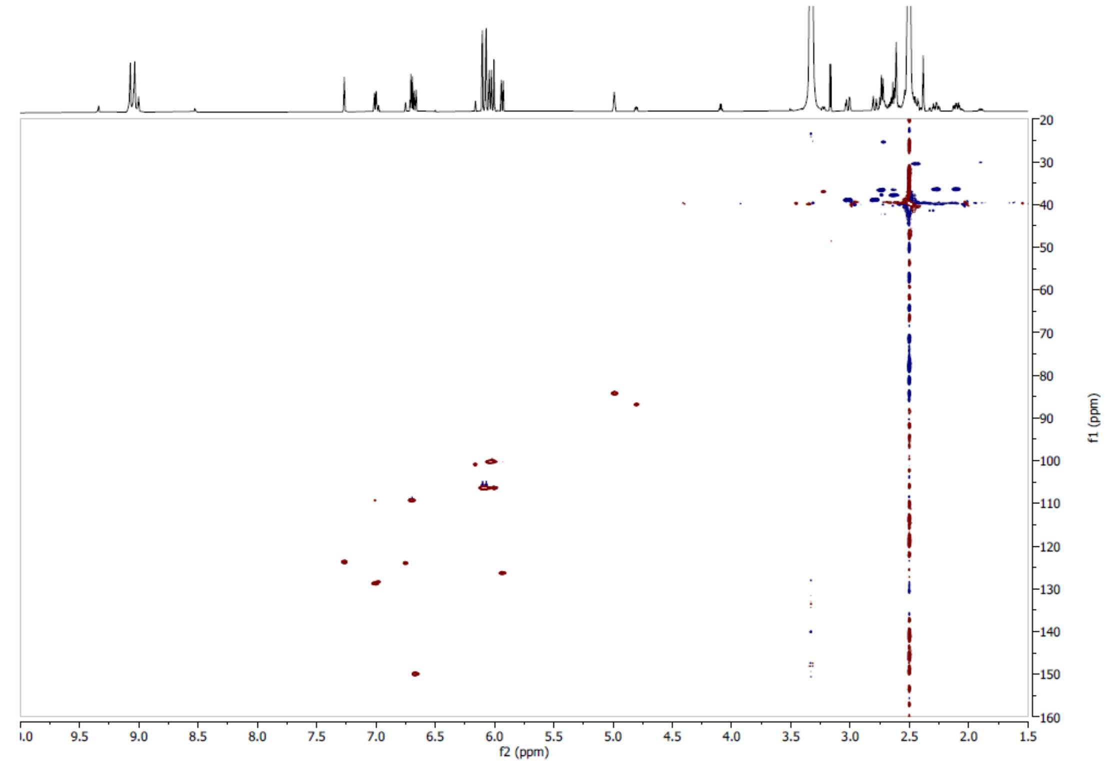

Figure S34. Edited HSQC NMR spectrum of compound **15** in DMSO-d_6

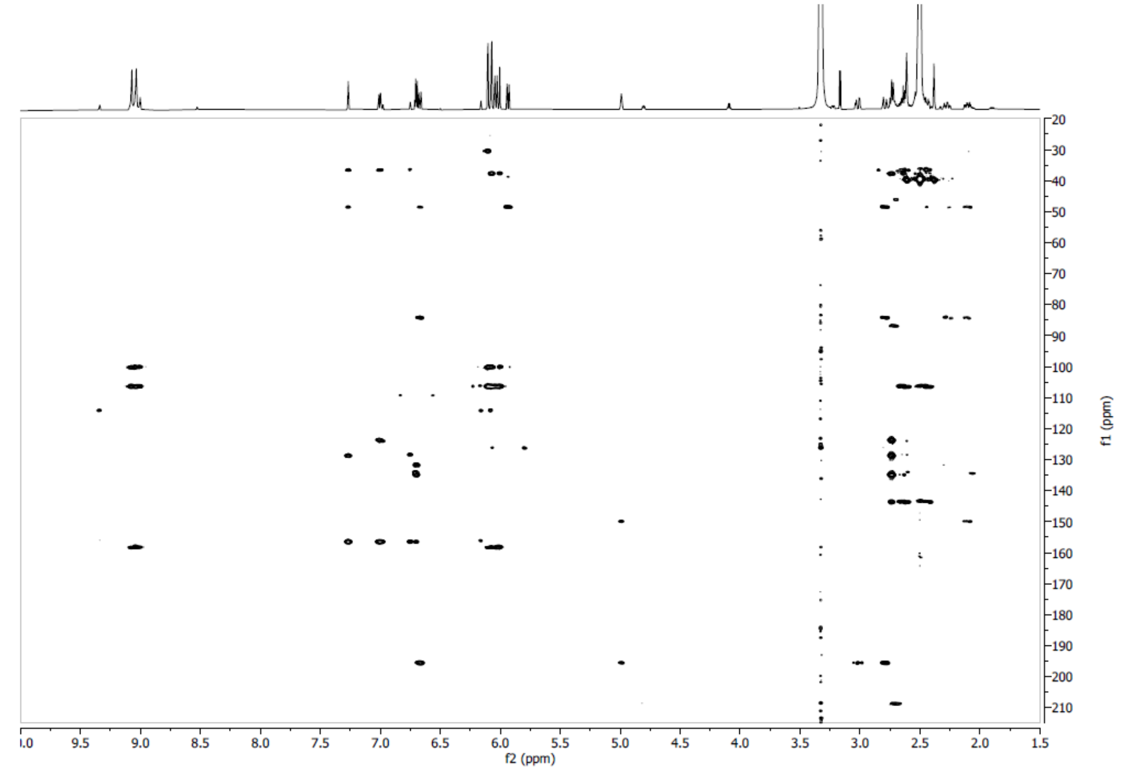

Figure S35. HMBC NMR spectrum of compound **15** in DMSO-d_6

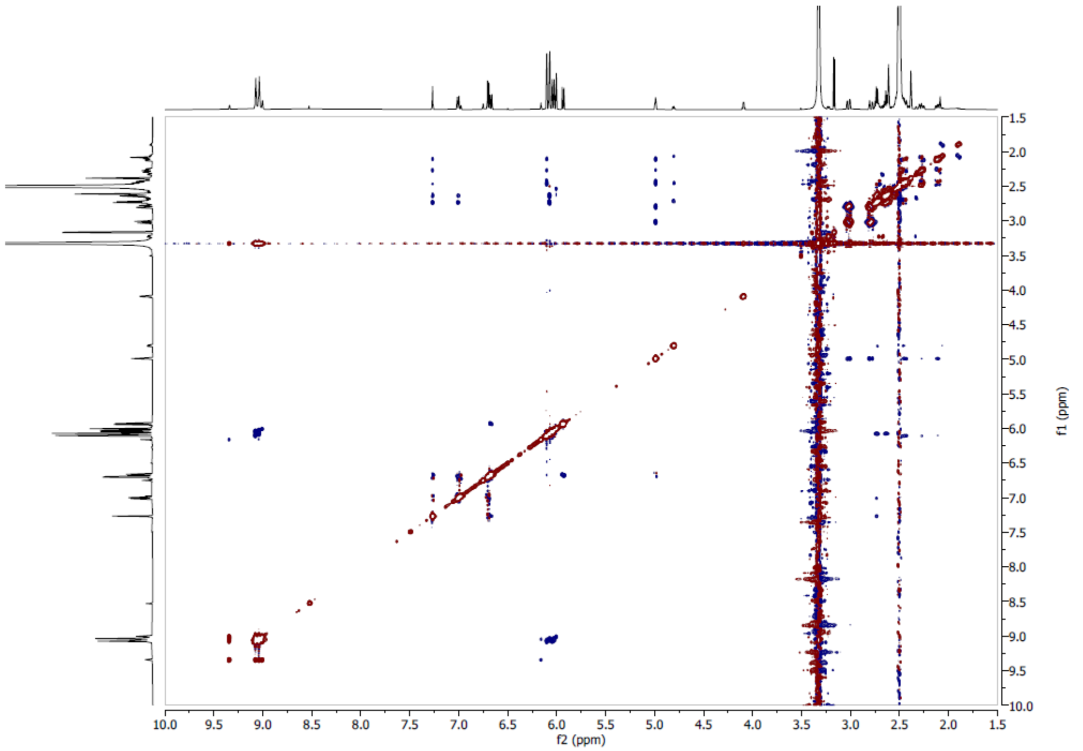

Figure S36. ROESY NMR spectrum of compound **15** in DMSO-d_6

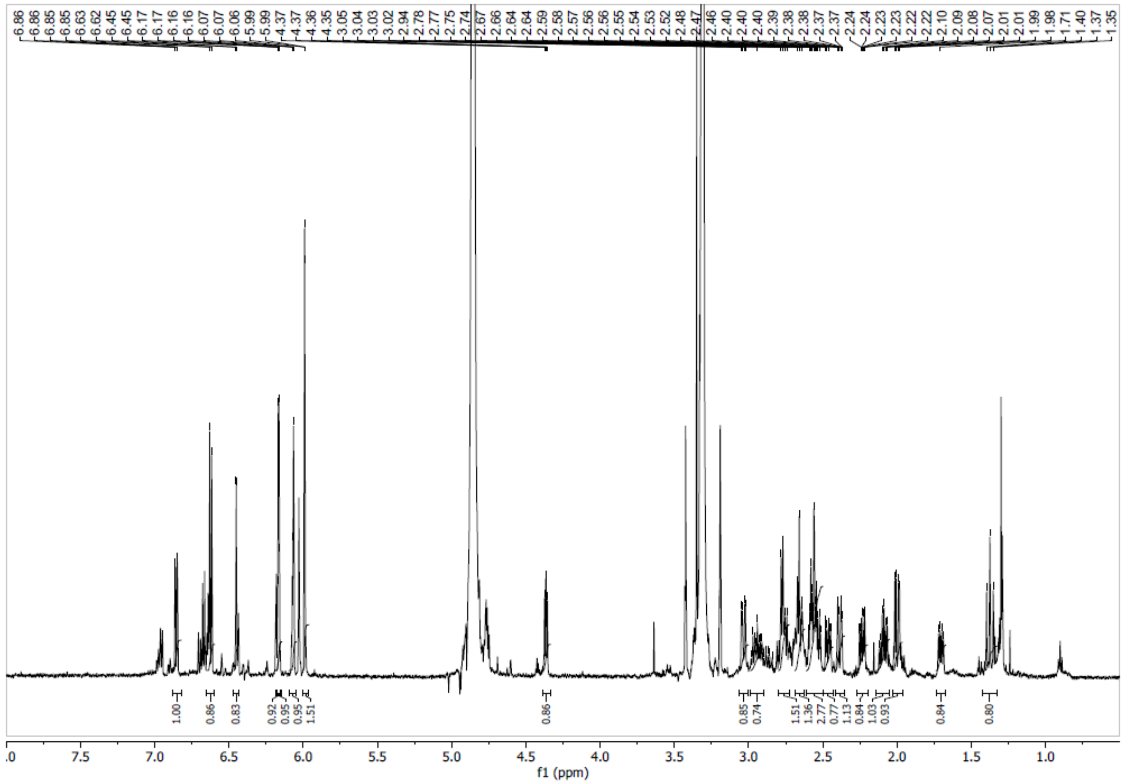

Figure S37. ^{1}H NMR spectrum of compound **16** in DMSO-d_6 at 600 MHz

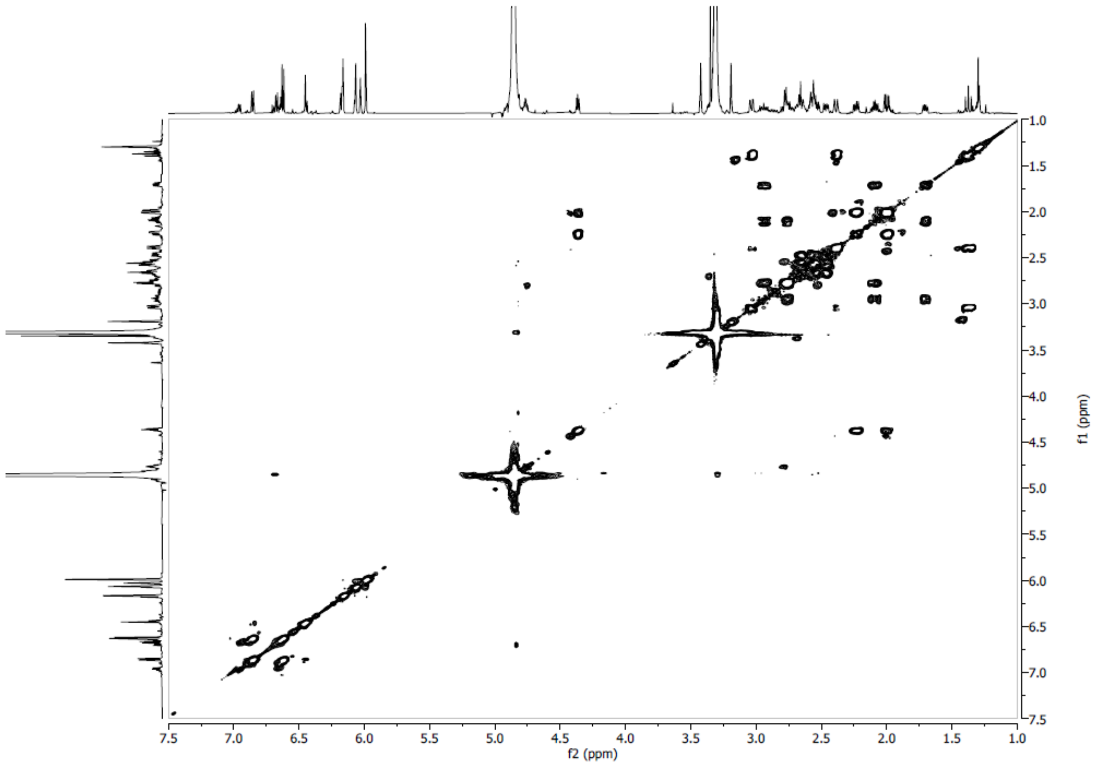

Figure S38. COSY NMR spectrum of compound **16** in DMSO-d_6

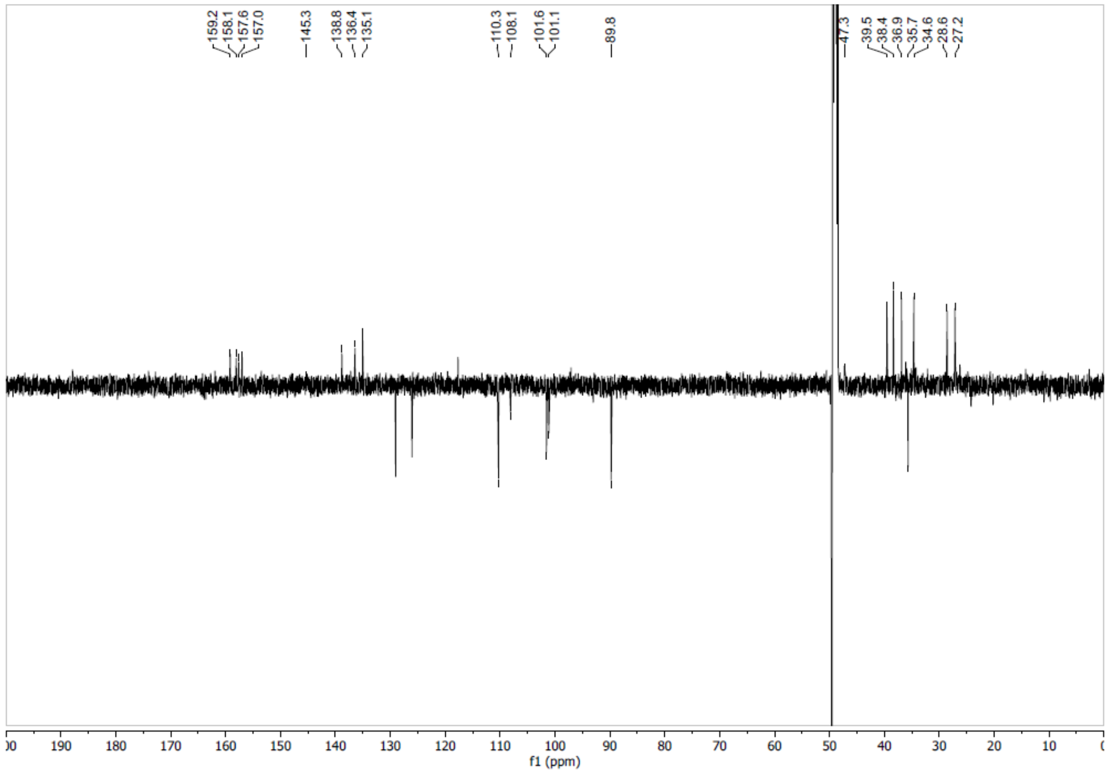

Figure S39. ^{13}C-DEPTQ NMR spectrum of compound **16** in DMSO-d_6 at 151 MHz

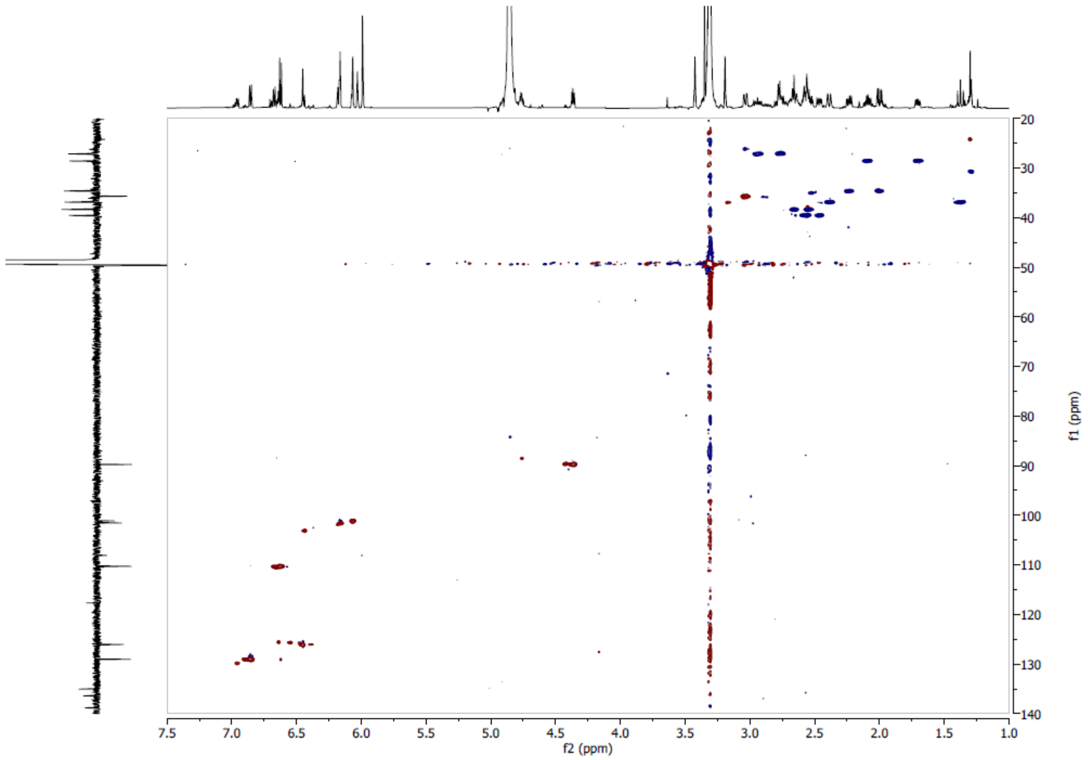

Figure S40. Edited HSQC NMR spectrum of compound **16** in DMSO-d_6

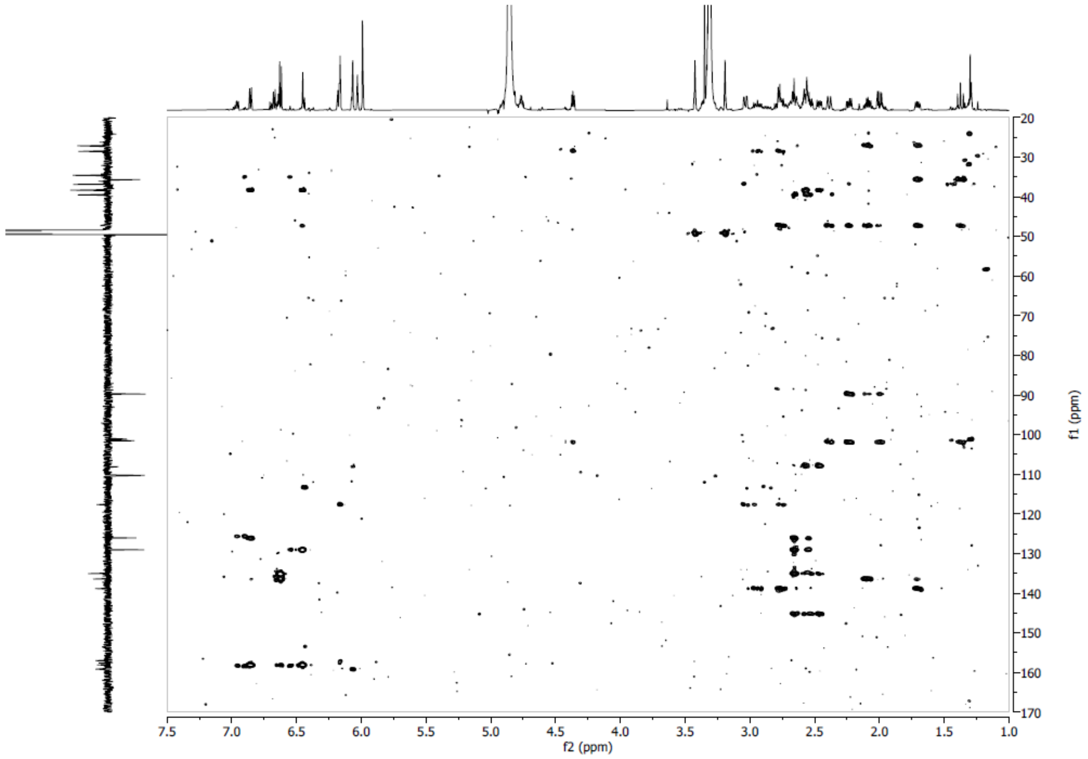

Figure S41. HMBC NMR spectrum of compound **16** in DMSO-d_6

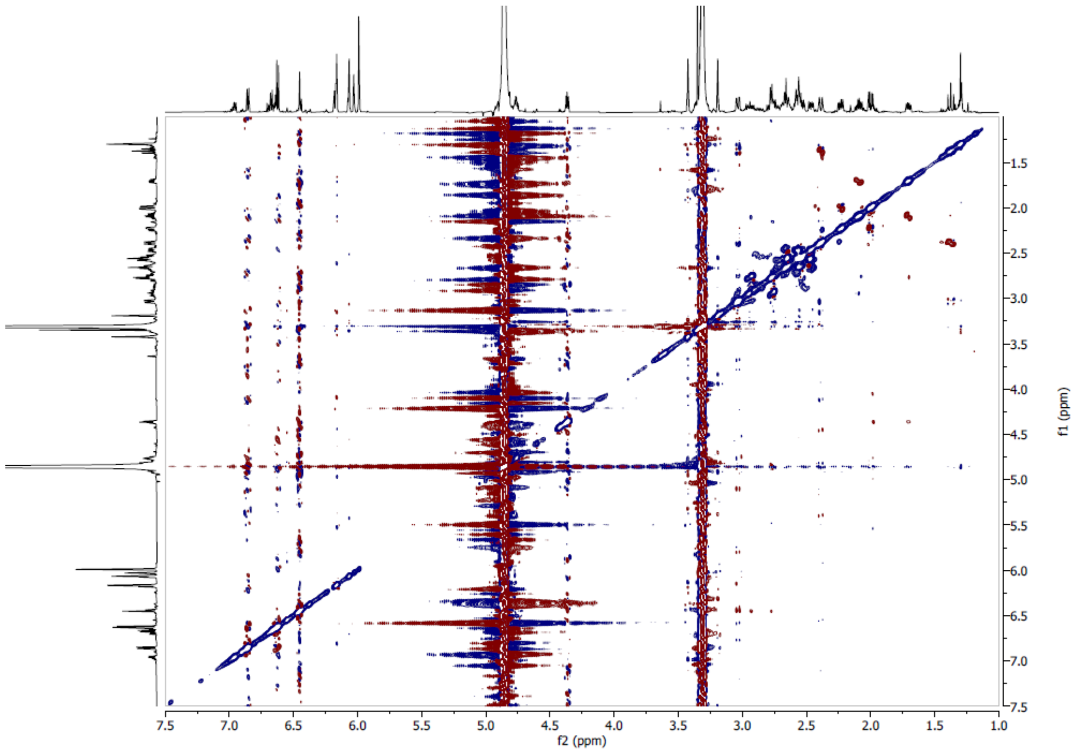

Figure S42. ROESY NMR spectrum of compound **16** in DMSO-d_6
